# Modulating the Electronic Properties of Conductive Conjugated Coordination Polymers with Atomic-Level Precision

**DOI:** 10.1021/jacs.6c06561

**Published:** 2026-09-15

**Authors:** Hyuk-Jun Noh, Doran L. Pennington, Mark Del Campo, Christopher H. Hendon, Katherine A. Mirica

**Affiliations:** † Department of Chemistry, Burke Laboratory, 3728 Dartmouth College, Hanover, New Hampshire 03755, United States; ‡ Department of Chemistry and Biochemistry, 3265 University of Oregon, Eugene, Oregon 97403, United States; § 298862 Rigaku Americas, The Woodlands, Texas 77381, United States

## Abstract

Conductive metal–organic frameworks and conjugated coordination polymers (CCPs) are promising platforms for low-power electronics and sustainable energy technologies, yet charge-transport design rules remain elusive. In diamine-based frameworks, Cu-coordinated motifs exhibit electrical conductivities several orders of magnitude lower than those of their Ni analogs, but the origin of this disparity has remained unclear, complicating rational selection of earth-abundant metal nodes for multifunctional materials. Here, we report the synthesis and atomic-resolution characterization of Cu-TABQ, a diamine-based CCP grown as single-crystals from tetraamino-*p*-benzoquinone and copper ions. Microcrystal electron diffraction reveals a distorted pseudosquare-planar Cu-coordination with asymmetric imine bonding, electron localization, and a structural model consistent with possible local protonation/hydrogen bonding. In contrast, Ni-TABQ adopts a planar, symmetric coordination environment that supports charge delocalization and near-metallic transport. Density functional theory suggests that protonation stabilizes the distorted Cu-TABQ structure and suppresses charge transport. Together, these results support an atomic-level explanation for metal-dependent transport in diamine-based frameworks and establish the metal–ligand coordination environment and protonation as tunable handles for designing conductive frameworks.

## Graphical abstract

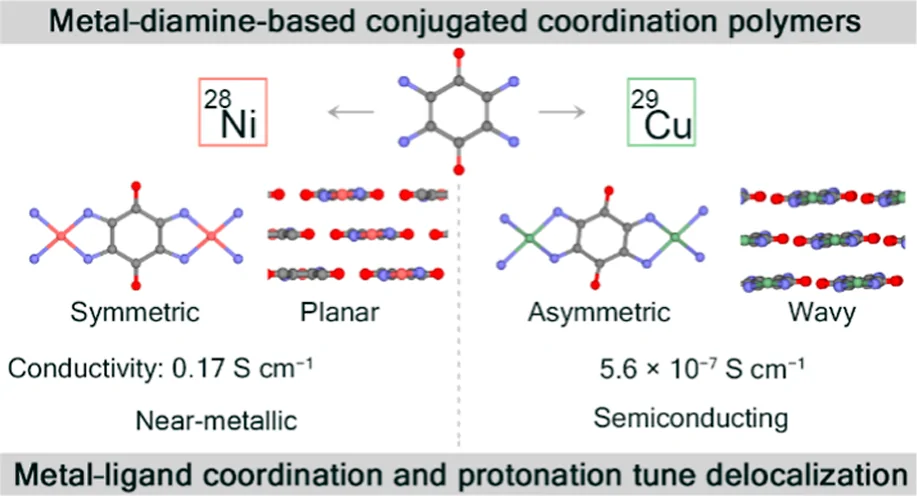

## Introduction

The emergence of electrically conductive metal–organic frameworks (cMOFs) and conjugated coordination polymers (CCPs) has offered opportunities for innovation in electronics,^1,2^ chemiresistive sensors,^3–5^ electrocatalysts,^6,7^ and energy storage.^8–12^ These framework materials are constructed from diverse combinations of organic ligands and bridging metal nodes, enabling in-plane extended π-d conjugation that facilitates “through-bond” charge transport.^13^ In addition, interlayer stacking in cMOFs and interchain stacking in CCPs can promote “through-space” charge transport.^14,15^ Understanding the dominant charge-transport pathways in these materials is essential for rationally engineering device-relevant performance in sustainability-focused applications, including low-power electronics, environmental monitoring, and clean energy conversion and storage.

Recent studies have highlighted the importance of “through-space” charge transport in conductive frameworks,^15–19^ motivating mechanistic structure–transport insight for device-relevant design. However, despite their outstanding electrical conductivity,^20,21^ the role of local metal–ligand coordination in governing electronic delocalization and charge transport in diamine-based cMOFs remains much less understood because their limited crystallinity has hindered atomic-level structure–transport correlations. This challenge stems, in part, from the electron-rich nature of phenylenediamine ligands, which complicates their isolation, handling, and incorporation into large, well-ordered crystallites.^22^ As a result, most diamine-based cMOFs are obtained only as polycrystalline powders, limiting direct interrogation of intrinsic structure–property relationships. In this context, CCPs provide a complementary and experimentally tractable model platform: they preserve the metal–diamine conjugation motifs that underpin conductivity in diamine-based frameworks, while their low-dimensional structures and tunable interchain interactions can yield microcrystalline or single-crystalline materials amenable to atomic-resolution characterization. Such model systems are particularly valuable for addressing a long-standing knowledge gap—why closely related Ni- and Cu-coordinated diamine-based frameworks reproducibly display orders-of-magnitude differences in electrical conductivity,^21,23,24^ and how metal identity governs electronic delocalization and charge transport.

Herein, we address this challenge by providing synthetic access to a crystalline diamine-based CCP, Cu-TABQ, via coordination of Cu ions with tetraamino-*p*-benzoquinone (TABQ) ligands. The resulting framework exhibits strong interchain hydrogen bonding between the quinone and amine functionality, which facilitates the formation of well-ordered single crystals. We resolved the crystal structure of Cu-TABQ using microcrystal electron diffraction (MicroED) with subangstrom precision (0.6 Å), enabling a detailed elucidation of the bonding environment in the Cu-bis­(iminobenzosemiquinoid) motif and providing critical insights into its electronic properties. Notably, Cu-bis­(iminobenzosemiquinoid) units in Cu-TABQ adopt a pseudosquare planar coordination geometry, characterized by two distinct imine bond lengths. MicroED further supports a structural model involving a possible local hydrogen-bonding/protonation motif at the nitrogen associated with the elongated C–N bond, which may contribute to the asymmetric and electronically localized structure of Cu-TABQ. This asymmetric bonding leads to a wavy, nonplanar intrachain structure, indicative of significant electron localization within the framework. In contrast, Ni-TABQ, whose structure was analyzed by refinement of PXRD data and supported by TEM characterization, exhibits a more symmetric coordination environment, consistent with previous reports and with a higher degree of electronic delocalization.^25^ This interpretation is further supported by the smaller optical band gap and red-shifted near-infrared absorption of Ni-TABQ compared with those of its Cu-analog, consistent with enhanced electronic delocalization. Consistent with these structural differences, Cu-TABQ and Ni-TABQ display a conductivity contrast of nearly six orders of magnitude at room temperature (5.6 × 10^–7^ vs 0.17 S cm^–1^), alongside a notably larger optical band gap for Cu-TABQ (1.05 eV) than for Ni-TABQ (0.67 eV). Density functional theory (DFT) further supports these experimental observations, revealing that protonation of the diamine ligand in Cu-TABQ stabilizes a distorted and asymmetric coordination environment that closely reproduces the MicroED-resolved crystal structure and the experimentally observed semiconducting electronic properties. In contrast, fully deprotonated Ni-TABQ converges to a planar structure with symmetric metal–ligand coordination and near-metallic charge transport. Together, these results provide direct atomic-level evidence linking the local metal–ligand coordination environment and protonation with electronic delocalization and charge-transport regimes in diamine-based metal-bis­(iminobenzosemiquinoid) frameworks and related materials—an aspect that has remained difficult to resolve in previous studies due to the lack of well-defined structural models. Our DFT-MicroED correlation further identifies protonation as a general “protonic dopant” handle that stabilizes symmetry-broken structures and switches transport from delocalized, near-metallic property to localized, semiconducting behavior, offering mechanistic insights and design considerations for multifunctional conductive frameworks to advance sustainable technologies in environmental monitoring, low-power electronics, and clean energy conversion and storage.

## Results and Discussion

### Design and Synthesis Control of M-TABQ CCPs

We selected TABQ as the ligand (Figures S1,S2) to synthesize M-TABQ CCPs (M: Cu, Ni). The quinone moieties in TABQ are capable of forming strong interchain hydrogen bonding within the CCP framework, promoting highly ordered structures. In addition, the electron-deficient quinone and electron-rich amine functionalities enable donor–acceptor (D–A) interactions upon metal coordination. Such D–A interactions are commonly employed to modulate the solid-state packing of conducting polymer chains, as they contribute to band gap narrowing and enhanced charge transport.^26–28^ Motivated by these features, we aimed to synthesize highly ordered TABQ-based CCPs by coordinating earth-abundant transition metals (Ni and Cu) through the bis­(iminobenzosemiquinoid) moiety of the organic ligand, thereby achieving microcrystalline- and single-crystalline materials (Figure 1). By holding the TABQ scaffold constant while varying only the metal node (Ni vs Cu), we test whether metal-dependent coordination symmetry controls electronic delocalization and thereby charge transport in diamine-based CCPs, providing an atomic-level basis for the long-observed conductivity disparity between Ni- and Cu-coordinated diamine frameworks.

**Figure 1:**
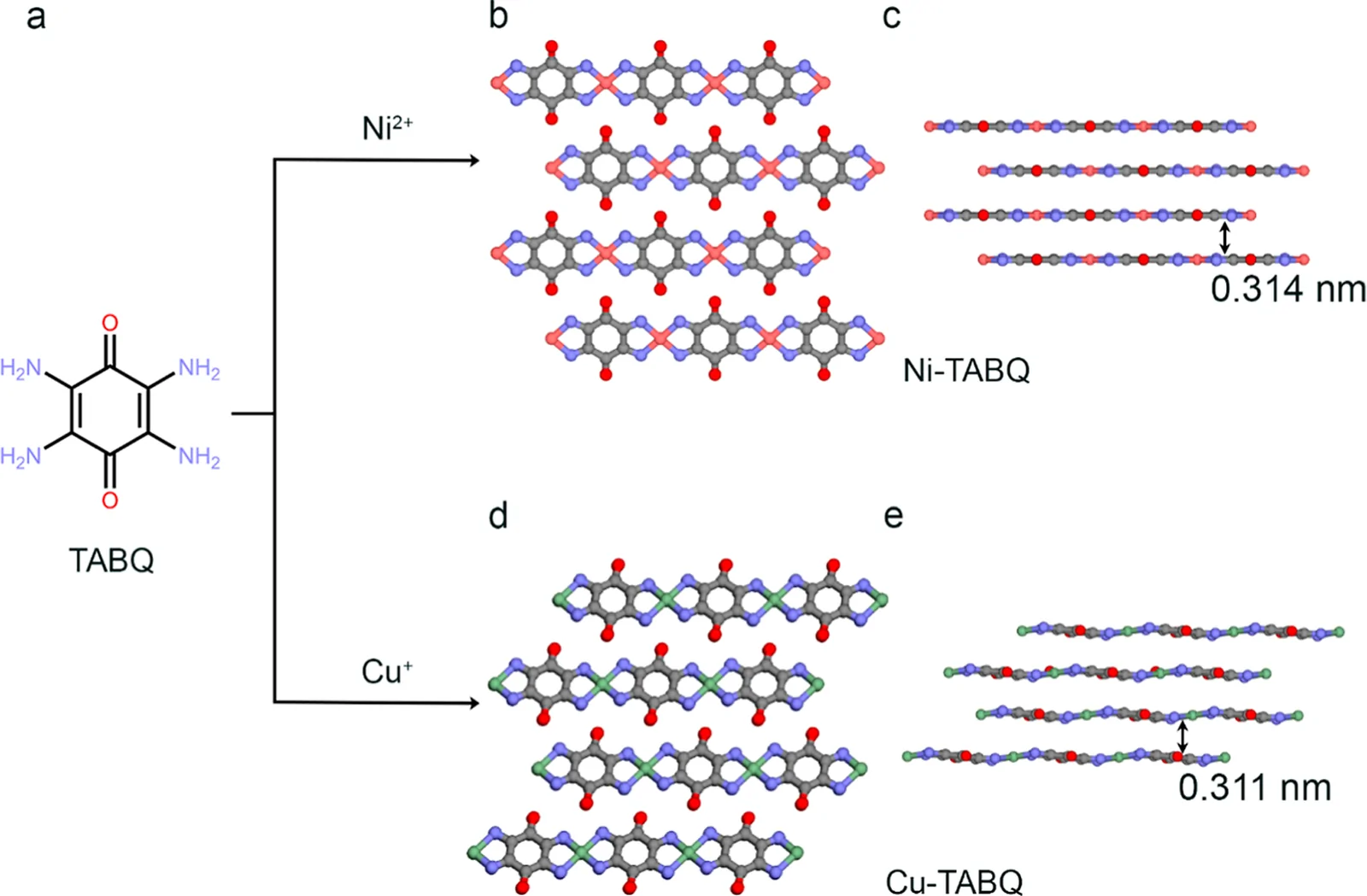
Schematic illustration of M-TABQ CCPs (M: Cu, Ni). (a) Synthetic conditions of M-TABQ CCPs. (b) Top and (c) side views of Ni-TABQ. (d) Top and (e) side views of Cu-TABQ. Hydrogen atoms are omitted for clarity. Color code: C, gray; N, blue; O, red; Ni, pink; Cu, green.

Through iterative optimization of the synthetic conditions, highly crystalline Ni-TABQ and Cu-TABQ CCPs were successfully obtained by solvothermal reaction between TABQ ligand and the corresponding metal precursor in the presence of concentrated aqueous ammonia under aerobic conditions (see Supporting Information for details). Ni-TABQ (NiC_6_H_4_N_4_O_2_) was synthesized following a previously reported procedure (Figure 1a–c).^25^ Notably, the use of Cu^+^ precursors, such as CuBr and CuI, instead of Cu^2+^ salts (e.g., Cu­(NO_3_)_2_) for the synthesis of most copper-based 2D cMOFs and CCPs, afforded Cu-TABQ (CuC_6_H_6_N_4_O_2_) with significantly higher crystallinity and larger crystallite size (Figures 1d–e, S3,S4 and Table S1, see Supporting Information for details). In both M-TABQ frameworks, the starting TABQ ligand undergoes metalation-induced conversion into a tetraimino-benzoquinone (TIBQ)-type coordination state, forming 1D chains composed of alternating metal-bis­(iminobenzosemiquinoid) units and square-planar-like metal centers. The distinct hydrogen contents in Ni-TABQ and Cu-TABQ reflect their different ligand protonation states, as determined from the crystallographic models.

### Structural Identification

To determine the crystal structure of materials, powder X-ray diffraction (PXRD) was employed. Rietveld refinement of the PXRD pattern of Ni-TABQ, using a previously reported structural model^25^ as the starting point, yielded the best fit in the space group *C*2/*m* with the unit cell of *a* = 12.1315 Å, *b* = 7.6425 Å, and *c* = 3.7671 Å (Figure 2a), in close agreement with the previously reported structure, although the unit–cell parameters are not identical to those of the previously reported Ni-1D material. Scanning electron microscopy (SEM) images exhibited the rectangular brick-shaped and short rod-like crystallites with lengths of approximately 200 nm (Figure 2b). A high-resolution transmission electron microscopy (HR-TEM) image of a Ni-TABQ crystallite oriented along the [100] direction displayed a well-defined square-type diffraction pattern (Figure 2c). The corresponding fast Fourier transform (FFT) pattern for this Ni-TABQ crystal showed two different reflections at 0.373 and 0.382 nm (Figure 2d), oriented nearly perpendicular to each other, consistent with the (001) and (020) planes identified in the PXRD pattern and in good agreement with the simulated FFT pattern (Figure 2e).

**Figure 2:**
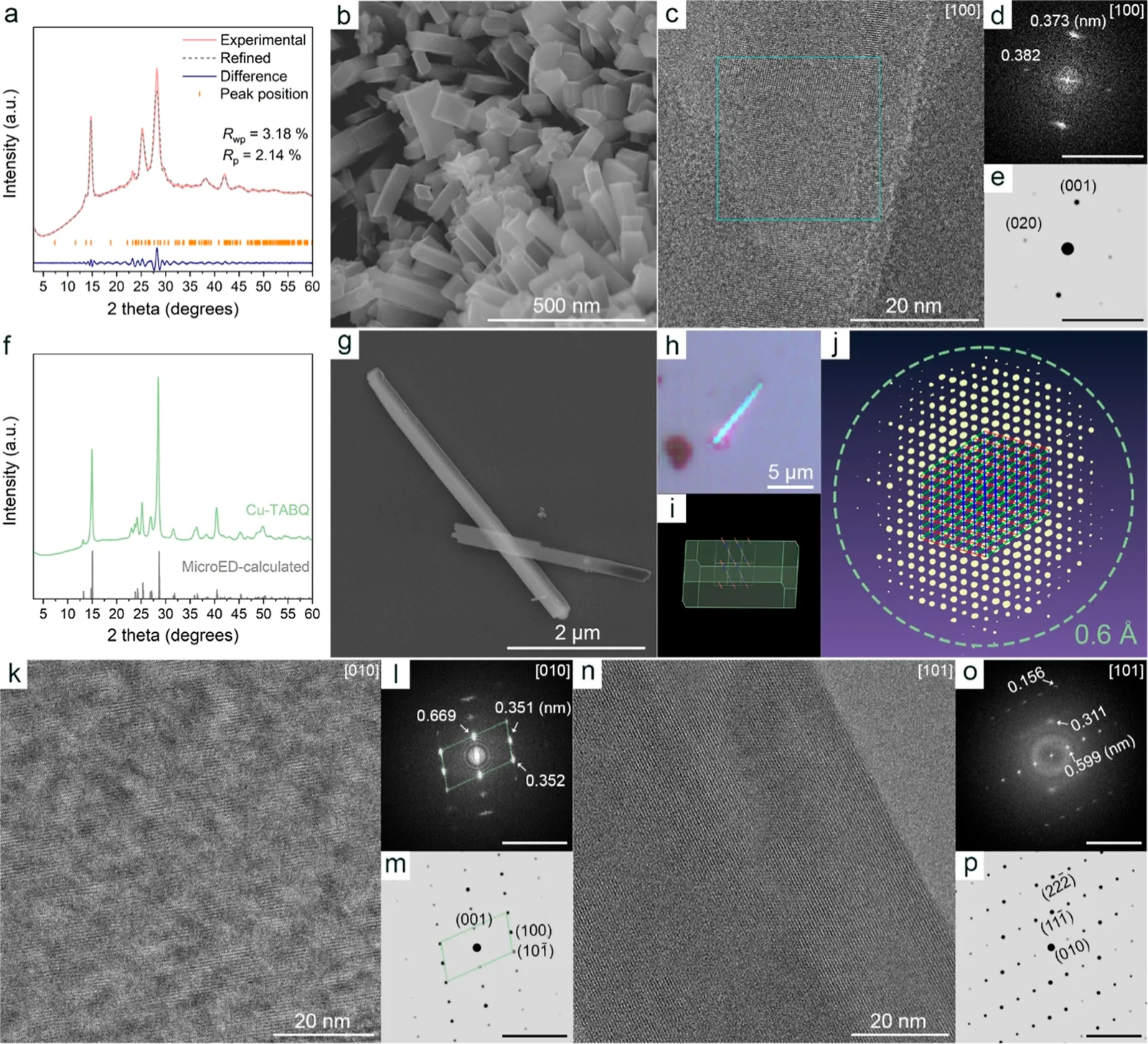
Structure and characterization of M-TABQ. (a) Experimental and Rietveld refined PXRD patterns of Ni-TABQ. (b) SEM image of Ni-TABQ microcrystals. (c) HR-TEM image of Ni-TABQ observed along the [100] direction. (d) Corresponding and (e) simulated FFT images. Scale bars: 5 nm^–1^. (f) Experimental and calculated PXRD patterns of Cu-TABQ. (g) SEM image of Cu-TABQ microcrystals. (h) Optical microscope image of material. (i) Calculated BFDH morphology of Cu-TABQ based on the single crystal structure. (j) 3D reciprocal lattices of a Cu-TABQ rod with a resolution down to 0.6 Å. (k) HR-TEM image of Cu-TABQ observed along the [010] direction. (l) Corresponding and (m) simulated FFT images. Scale bars: 5 nm^–1^. (n) HR-TEM image of Cu-TABQ observed along the [101] direction. (o) Corresponding and (p) simulated FFT images. Scale bars: 5 nm^–1^.

Cu-TABQ exhibited more numerous and sharper diffraction peaks in the PXRD pattern than Ni-TABQ, which may be attributed to its lower symmetry crystal structure and superior crystallinity, respectively (Figure 2f). Unlike the rectangular brick-shaped and short rod-like crystallites of Ni-TABQ, SEM and optical microscopy showed that Cu-TABQ forms well-defined, rod-like crystallites with lengths extending up to tens of micrometers (Figures 2g,h and S5), consistent with the theoretically predicted Bravais-Friedel-Donnay-Harker (BFDH) morphology of Cu-TABQ derived from the single-crystal structure (Figure 2i). Benefiting from the high quality and large crystallites of Cu-TABQ, the single-crystal structure of the material was revealed by using MicroED with atomic-level precision. The crystals are of sufficient size and crystallinity to allow structural refinement with a high resolution of up to 0.6 Å (Figures 2j, S6, S7, Tables S2 and S3), which revealed that Cu-TABQ crystallized in the triclinic system, *P*-*1* space group with the unit cell *a* = 3.6540(5) Å, *b* = 6.6455(12) Å, and *c* = 7.5913(8) Å. The experimental PXRD patterns matched well with the simulated patterns generated from its single-crystal structure, confirming phase-pure Cu-TABQ (Figure 2f).

The crystal structure of Cu-TABQ was further confirmed by HR-TEM. HR-TEM images revealed that Cu-TABQ crystallites display long-range ordering across the entire crystallite, accompanied by clear electron diffraction patterns (Figures 2k–p, S7 and S8). HR-TEM image acquired along the [010] direction (Figure 2k) and its corresponding fast Fourier transform (FFT) (Figure 2l) displayed a well-defined parallelogram-shaped lattice and diffraction pattern, consistent with the simulated FFT (Figure 2m). The analysis revealed an inclined interchain π–π stacking distance of 0.351 nm (Figure S9a) and the Cu–Cu plane spacing of 0.669 nm (Figure S9b), corresponding to (100) and (001) facets, respectively, as observed by simulated FFT, consistent with PXRD patterns. Imaging perpendicular to the long axis of the Cu-TABQ rod crystal enabled the visualization of highly ordered structures along the [101] direction (Figure 2n). The corresponding FFT showed the closest reflections at 1.67 nm^–1^ (Figure 2o), corresponding to a real-space periodicity of 0.599 nm, which matches the (010) plane observed in PXRD and agrees with the simulated FFT pattern (Figure 2p).

Based on the refinement of crystal structure of M-TABQ CCPs, Ni-TABQ exhibited an almost planar structure with a symmetric coordination between Ni^2+^ centers and the TIBQ ligands (Figure 3a). This structural symmetry, along with strong interchain hydrogen bonding (Figure 3b), accounts for the rectangular brick-/rod-shaped crystallites observed in SEM images (Figure 2b), as well as the square-type electron diffraction patterns observed in TEM analysis (Figure 2c). Notably, the TIBQ ligands in Ni-TABQ can be regarded as comprising two N–C–C–C–N 5-center, 6π (5c-6π) conjugated subsystems. The C–C and C–N lengths within these subsystems are remarkably similar, observed as 1.394 and 1.396 Å, respectively (Figures 3a and S11a). This delocalized 6π conjugation serves as significantly efficient electronic communication bridges between two Ni^2+^ centers, contributing to the observed planar intrachain structure of Ni-TABQ (Figure 3c). These structural features are in close agreement with those previously reported for Ni-TABQ,^25^ and are similarly observed in 2,5-diaminobenzoquinonediimine (DABQDI) metal complexes, such as Ni-TABQDI tapes.^29^

**Figure 3:**
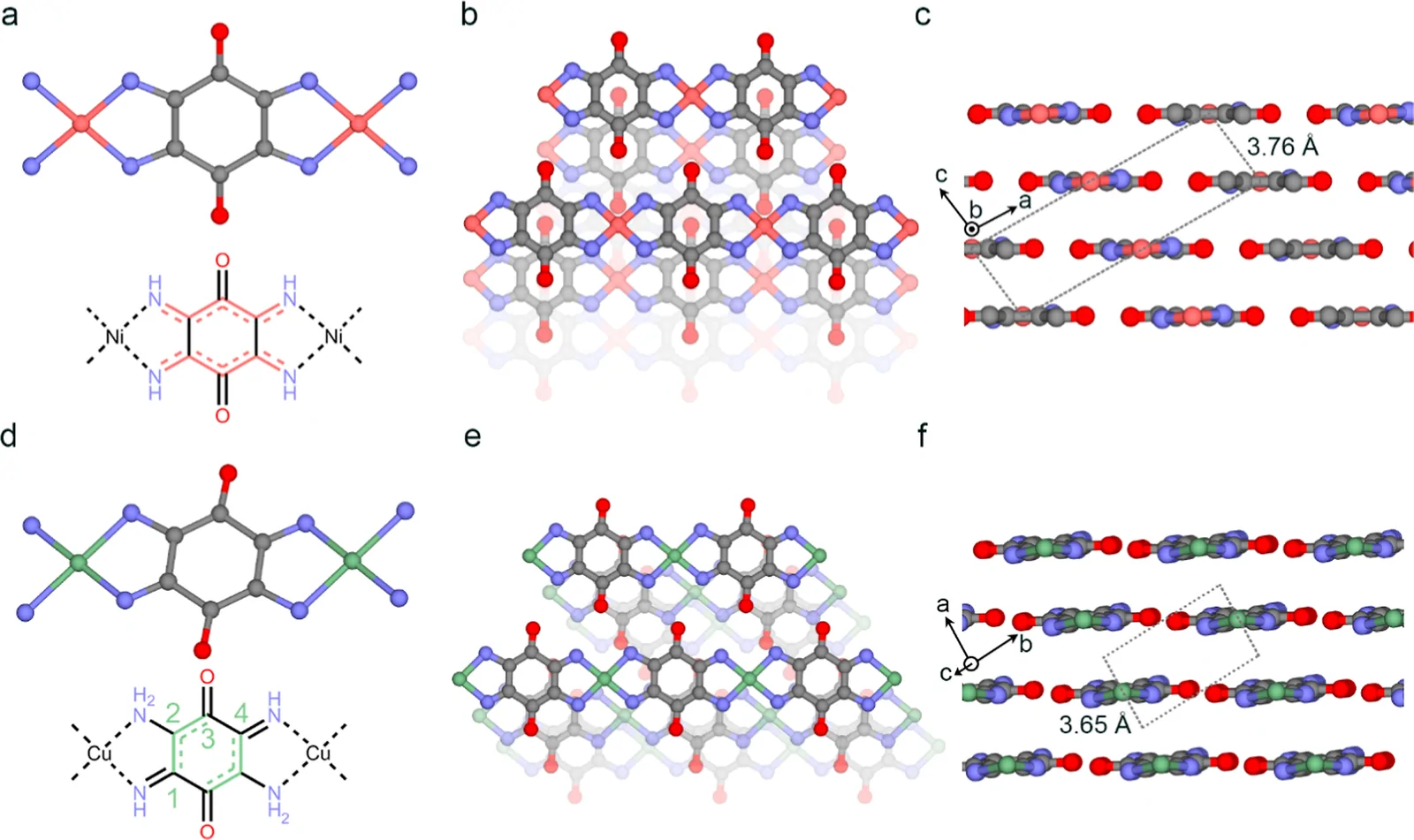
Structural comparison of Ni-TABQ and Cu-TABQ. (a) Structural unit of Ni-TABQ and its corresponding Lewis structure. (b) Top-down view of the interchain D–A π–π stacking in Ni-TABQ. (c) Interchain planar D–A π–π stacking of Ni-TABQ. (d) Structural unit of Cu-TABQ and the corresponding crystallographically derived connectivity/protonation model. The lower schematic illustrates a proposed N-bound protonation pattern supported by the structural and spectroscopic analyses, rather than a unique charge-localized Lewis structure. (e) Top-down view of the interchain D–A π–π stacking in Cu-TABQ. (f) Interchain nonplanar D–A π–π stacking of Cu-TABQ. Hydrogen atoms are omitted from the crystallographic and packing models for clarity.

In contrast to the symmetric coordination between Ni^2+^ ions and the TIBQ ligands in Ni-TABQ, Cu-bis­(iminobenzosemiquinoid) units in Cu-TABQ adopt a distorted square-planar coordination geometry, featuring two distinct imine bond lengths: a longer single-bond-like C–N distance of 1.440(7) Å, which exceeds that of the C–N bond in the free TABQ ligand (Figure S10),^30^ and a shorter double-bond-like C=N distance of 1.286(5) Å (Figures 3d and S11b). These pronounced differences in bond lengths indicate significant electron localization within the framework. Notably, during crystallographic refinement of the Cu-TABQ structure determined by MicroED, a positive Coulombic/difference-map peak was observed near the nitrogen atom associated with the elongated C–N bond. This peak could be modeled as an additional N-bound hydrogen atom forming a possible intermolecular N–H···O interaction with a neighboring carbonyl oxygen (Figures S12,S13, see the Supporting Information for details). This structural model is consistent with a local protonation/hydrogen-bonding motif that may contribute to the localized and asymmetric bonding environment in Cu-TABQ, in agreement with the electronic localization discussed below. Additionally, the C–C bond lengths within the TIBQ units in Cu-TABQ were found to be 1.394(6), 1.402(9), and 1.522(8) Å (Figure S11b), suggesting a partially conjugated system across the C1–C3 segment, interrupted by the elongated single C3–C4 bond, which is even longer than the C–C bond in the free TABQ ligand (Figures 3d and S10). This asymmetric coordination gives rise to inclined interchain hydrogen bonding (Figure 3e), contributing to the parallelogram-shaped lattice and the corresponding FFT pattern (Figure 2k–m). Notably, the intrachain 1D polymeric structure adopts a wavy, nonplanar structure, which is attributed to the asymmetric bonding environment (Figure 3f), in contrast to the nearly planar intrachain structure in Ni-TABQ.

This metal–ligand coordination in the M-TABQ CCPs was further verified by Raman spectroscopy (Figure S14). Ni-TABQ showed a single Ni–N stretching peak at 430 cm^–1^, whereas Cu-TABQ displayed two distinct Cu–N stretching peaks at 391 and 424 cm^–1^, consistent with the asymmetric coordination environment observed in the crystal structure. The geometry of metal–ligand coordination was further supported by electron paramagnetic resonance (EPR) spectroscopy. Ni-TABQ showed a weak EPR signal with a slight shift in *g*-value compared to that of the TABQ ligand, suggesting partial contribution from Ni *d* orbitals while remaining largely EPR silent for square-planar Ni^2+^ (Figure S15). In contrast, Cu-TABQ exhibited three distinct and highly anisotropic EPR signals at *g* = 2.027, 2.041, and 2.167, consistent with the pseudosquare planar coordination geometry of paramagnetic Cu^2+^ observed in the crystal structure (Figure S15, see Supporting Information for details).^31–33^ Temperature-dependent magnetic susceptibility measurements show a reduced χ_M_ *T* value (∼0.25 cm^3^ K mol^–1^ at 298 K) and a small negative Weiss temperature (θ = – 0.75 K), consistent with a reduced apparent spin population and weak antiferromagnetic interactions in Cu-TABQ (Figure S16, see Supporting Information for details).

Infrared spectroscopy revealed systematic shifts in the C=O and C–N stretching modes upon metal coordination, consistent with increased electronic delocalization in the framework, with Ni-TABQ exhibiting more pronounced red-shifts than Cu-TABQ (Figure S17).^25,34^ In addition, the more complex N–H stretching profile of Cu-TABQ compared with Ni-TABQ is consistent with the presence of distinct –NH and –NH_2_ environments (Figure S17c, see Supporting Information for details). Consistent with this trend, Ni-TABQ also displayed higher thermal stability than Cu-TABQ, as evidenced by thermogravimetric analysis (Figure S18), reflecting the greater degree of electronic delocalization associated with its more symmetric coordination environment.^35^ Both materials exhibited low BET surface areas, consistent with their dense, nonporous one-dimensional chain structures (Figure S19).^25,36,37^

X-ray photoelectron spectroscopy (XPS) measurements support the presence of bis­(iminobenzosemiquinoid) units in M-TABQ (Figures S20,S21). Notably, Cu-TABQ shows predominantly Cu^2+^ character despite using a Cu^+^ precursor, which may arise from rapid oxidation upon exposure to ambient conditions after synthesis, but is also consistent with partial oxidation occurring during the solvothermal reaction and potentially influencing coordination behavior, as reflected by the improved crystallinity compared to the use of Cu^2+^ salts (see Supporting Information for details). By contrast, Ni-TABQ exhibits exclusively Ni^2+^ features in the Ni 2p spectrum.

### Electronic Structure and Charge Transport Properties

The degree of charge delocalization and metal–ligand conjugation in the M-TABQ CCPs were assessed by ultraviolet (UV)-visible (vis) near-infrared (NIR) spectra (Figure 4a). Both materials exhibited nearly identical electronic absorption peaks centered around 500 nm, corresponding to ligand-centered (LC) π–π* intraquinone transitions.^29,38^ Broad metal-to-ligand charge transfer (MLCT) bands were observed at 617 nm for Cu-TABQ and 781 nm for Ni-TABQ.^29^ The significantly red-shifted MLCT band in Ni-TABQ suggests enhanced electronic delocalization between Ni^2+^ centers and the TIBQ orbitals. Additionally, two broad NIR bands, centered at 838 nm for Cu-TABQ and 1138 nm for Ni-TABQ, are attributed to extended intrachain metal–ligand *d*–π conjugation.^25,36,39^ The further red-shifted NIR band of Ni-TABQ indicates greater charge delocalization along the polymer chain and stronger electronic coupling between Ni^2+^ and the TABQ ligands. The optical band gap of the CCPs was extracted from a Tauc plot (Inset in Figure 4a), which was found to be 1.05 and 0.67 eV for Cu-TABQ and Ni-TABQ, respectively. These band gap values further support the significantly higher degree of charge delocalization in Ni-TABQ.

**Figure 4:**
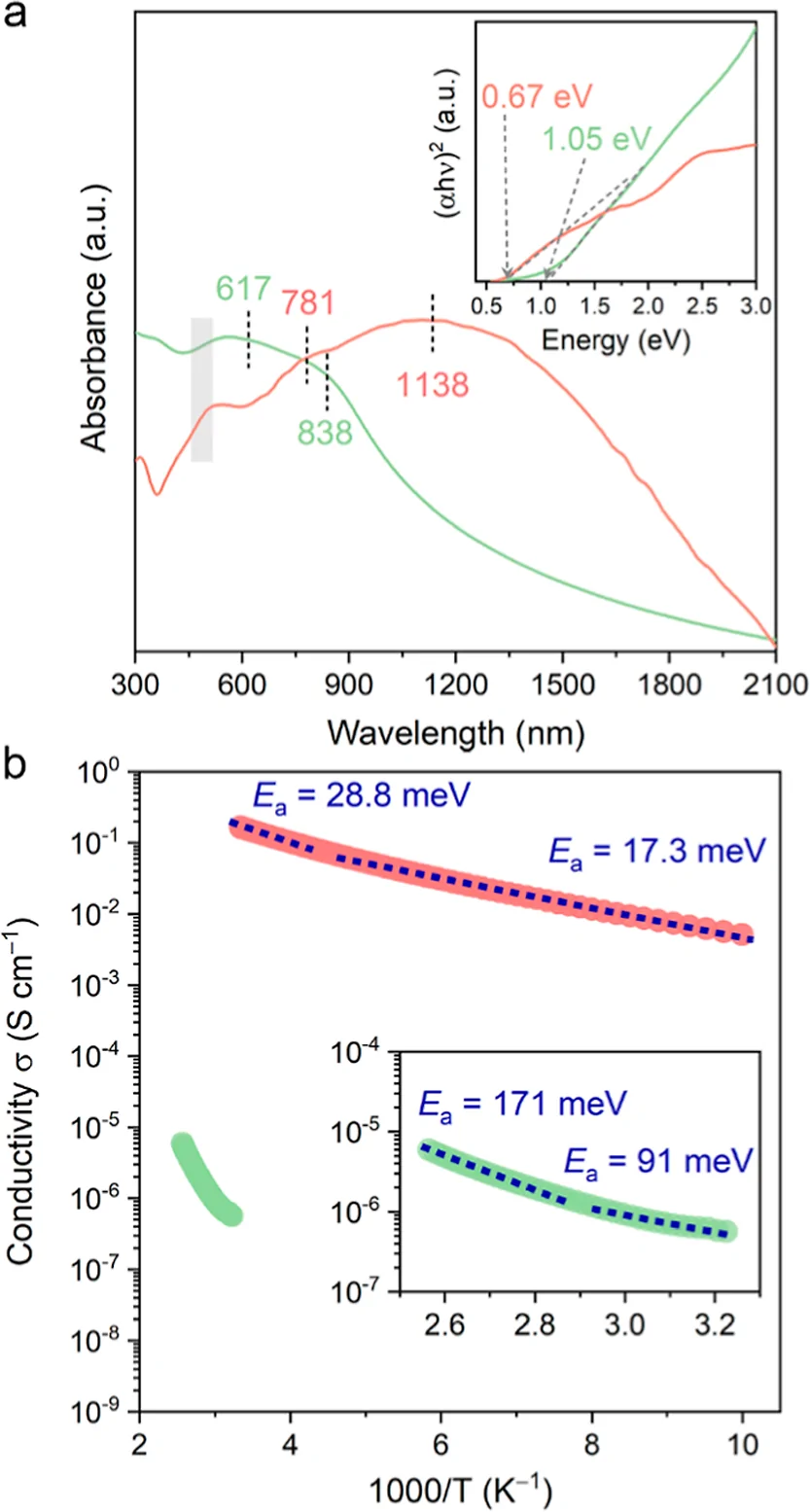
Electronic properties of M-TABQ CCPs. (a) UV–vis–NIR spectra of Cu-TABQ and Ni-TABQ deposited on quartz substrate. (b) Temperature-dependent electrical conductivity of Cu-TABQ (green line) and Ni-TABQ (pink line), plotted according to the Arrhenius equation.

The bulk electrical conductivities of the M-TABQ CCPs were measured using a four-contact probe method on pressed pellets (Figure S22). Notably, Cu-TABQ and Ni-TABQ exhibit a difference of nearly six orders of magnitude in electrical conductivity, with values of 5.6 × 10^–7^ and 0.17 S cm^–1^ at room temperature, respectively. This pronounced conductivity disparity between Cu- and Ni-coordinated diamine-based frameworks has also been consistently reported in prior studies.^20–24^ Such a trend indicates that the intrinsic difference in metal identity, particularly in terms of *d*–π orbital overlap and conjugation strength, can directly influence the resulting bonding environment. In this context, the relatively weaker and less effective *d*–π overlap in Cu centers leads to asymmetric Cu–diamine coordination, resulting in inefficient charge delocalization and suppressed conductivity. By contrast, the stronger and more effective orbital overlap associated with Ni centers favors a more symmetric bonding environment, enabling enhanced metal–diamine conjugation, efficient through-bond charge transport, and near-metallic conductivity. These results demonstrate that the extent of metal–diamine conjugation, governed by the local coordination environment of the metal node, is the dominant factor controlling charge delocalization and charge transport in diamine-based CCPs and related MOFs.

Variable-temperature conductivity measurements (see the Supporting Information for details) revealed semiconducting behavior in both M-TABQ CCPs (Figures 4b and S23). Both materials exhibit Arrhenius-type temperature dependence in their bulk electrical conductivities (see Supporting Information for details), with changes in activation energy at characteristic temperature regimes. In the Ni-TABQ system, such changes were correlated with reversible temperature-dependent structural variations, including increased interchain π–π stacking distance and larger intrachain dihedral angle at elevated temperature.^25^ For Cu-TABQ, in situ IR measurements show a reversible shift of the N–H stretching band from 3024 to 3040 cm^–1^ upon heating to 100 °C, followed by recovery upon cooling, indicating a reversible change in the local N–H···O hydrogen-bonding environment (Figure S24, see Supporting Information for details).^40,41^ This thermally responsive hydrogen-bonding motif may contribute to the observed change in activation energy. This observation contrasts with typical 2D layered cMOFs, which generally display a single *E*_a_ value owing to their more robust interlayer coupling. To further investigate the charge transport properties of M-TABQ CCPs, we performed Hall effect measurements on pressed pellets using the van der Pauw method at room temperature (Figure S25). Notably, Ni-TABQ exhibited an estimated electron mobility (μ_e_) of 0.050 (μ_e, average_ = 0.031 (±0.023)) cm^2^ V^–1^ s^–1^ and a carrier concentration (*n*_e_) of 1.24 × 10^20^ (*n*_e, average_ = 6.25 (±5.46) × 10^19^) cm^–3^, after antisymmetrization of the Hall data,^42,43^ comparable to those of other reported cMOFs (Figure S26).^44–47^ While the Ni-TABQ pellets generated a distinct Hall effect, we were unable to measure the Hall effect in pressed pellets of Cu-TABQ due to the intrinsically low mobility and insulator-like conductivity,^45,48^ which fall below the measurable range for Hall measurements. This observation is consistent with the significantly lower electrical conductivity of Cu-TABQ determined from four-contact probe measurements.

### Theoretical Calculations

The contrasting electronic properties of the M-TABQ materials were further assessed using DFT. The Ni-TABQ model converged to a planar layered structure with identical C–N bond lengths, recovering the symmetric coordination environment around the Ni^2+^ center observed by PXRD (Figure 5a). This structural symmetry indicates a high degree of electron delocalization.

**Figure 5:**
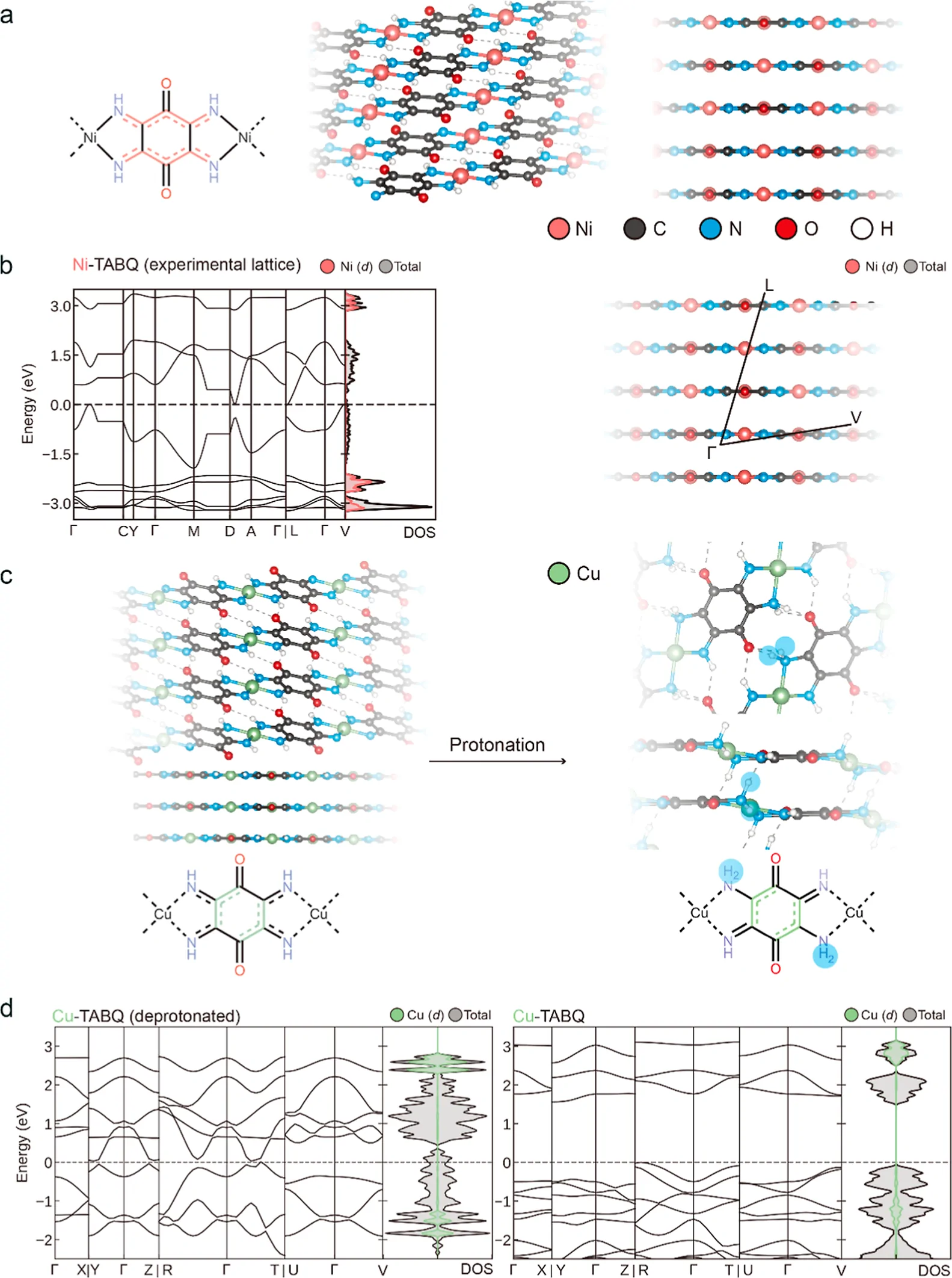
Electronic band structures of M-TABQ CCPs. (a) Optimized structure of Ni-TABQ, showing planar stacking and deprotonated TIBQ ligand. (b) Electronic band structure of Ni-TABQ, showing through-space (Γ–L, electron) and through-bond (Γ–Y, hole) contributions to the density of states at the Fermi level. (c) Optimized structure of Cu-TABQ, showing planar stacking (left) which buckles upon protonation of the ligand (right). (d) Electronic band structure of Cu-TABQ, showing metallic character when the ligand is deprotonated (left) and semiconducting property when the ligand is protonated (right).

Full relaxation of the unit cell, however, compressed the interlayer spacing by 11% relative to the Rietveld value (3.34 Å vs 3.77 Å) and yielded a metallic band structure (Figure S27). Prior DFT studies of 2D coordination polymers have established a strong dependence of metallicity and band gap on interlayer spacing.^18^ Given that the observed transport properties are more characteristic of a near-metallic semiconductor rather than a true metal, we then evaluated the electronic structure at the experimentally determined lattice parameters, relaxing only the internal coordinates.

When the experimental lattice parameters were fixed, a nearly metallic band structure was recovered, featuring a 0.33 eV direct gap at L (Figure 5b). Because the extrapolated Tauc onset of 0.67 eV is near the long-wavelength limit of the measured spectral window (0.59 eV), the Tauc onset therefore represents an upper bound on the optical gap. Integration of the first conduction band gives a carrier density of 5.9 × 10^19^ cm^–3^ at 300 K, in excellent agreement with the experimental average carrier density of 6.25 × 10^19^ cm^–3^. The near-metallic band structure includes contributions to the density of states at the Fermi level from smearing of the eigenvalues at the L and V high-symmetry points. The carriers at L are electrons and are predominantly transported through-space along the interlayer stacking direction, and the carriers at V are predominantly transported through-bond along the covalent and hydrogen-bonded axes. Electron and hole transport are thus predicted to be anisotropic and spatially segregated in Ni-TABQ.

When Cu-TABQ was modeled similarly, DFT yielded a planar material with a symmetric coordination environment, inconsistent with the experimental diffraction data. Protonation of two *p*-imino sites in the TIBQ ligand to the corresponding amine groups resulted in a distorted, wavy structure with significant electron localization featuring interchain hydrogen bonding (Figure 5c). In this model, the C=NH bond distance of 1.315 Å and the C–NH_2_ bond distance of 1.428 Å are each within 0.03 Å of the experimental bond distances measured by MicroED. The Cu centers adopt a *d* ^9^ electron configuration indicative of Cu^2+^ stemming from localized anionic imino-N sites rather than distributed and delocalized charges. Comparing the two Cu-TABQ models shows that this structural change alone governs the transport properties. The deprotonated model displays metallic character, whereas the distorted, protonated structure is decidedly semiconducting, with a 1.56 eV indirect band gap and no bands crossing the Fermi level (Figure 5d). Parabolic fitting of the band edges in the protonated model yields effective masses of −1.6 m_h_ along the R–Γ wavevector and 4.3 m_e_ along Y–Γ (Figure S28).

These models are consistent with the variable temperature conductivity measurements (Figure 4b), although the measurements were performed on pressed pellets, where interparticle transport across grain boundaries can dominate and give rise to thermally activated hopping. Nevertheless, despite its smaller crystallite size, Ni-TABQ exhibits activation energies of 28.8 meV (300–240 K) and 17.3 meV (220–100 K)–comparable to *k*_*B*_ *T* at room temperature and supporting transport governed by an intrinsically near-metallic electronic structure.^15,16,49^ In contrast, Cu-TABQ shows a substantially larger thermally activated response expected for a semiconducting material. Importantly, this contrast persists despite the larger crystallite size of Cu-TABQ, reinforcing that the observed transport disparity is dominated by intrinsic electronic structure. Complementary vibrational, frontier-band charge-density, and control band-structure calculations are also consistent with this interpretation and further support the important roles of local coordination geometry and protonation in governing the electronic properties of M-TABQ (Figures S29–S32). Together, these results suggest that proton-induced electron localization suppresses through-bond charge transport, while the distorted stacking geometry in Cu-TABQ disrupts through-space transport, ultimately rendering the material semiconducting.

## Conclusions

Despite significant interest in the electronic properties of cMOFs and CCPs, the scarcity of well-defined single-crystalline model systems has hindered a mechanistic understanding of how metal–ligand coordination governs charge transport. Here, we show that coordination of the TABQ ligand with transition metals (Ni or Cu) enables microcrystalline and single-crystalline bis­(iminobenzosemiquinoid)-based CCPs (M-TABQ, *M*: Ni, or Cu), supported by strong interchain hydrogen bonding. Using subangstrom MicroED (0.6 Å), we resolve the Cu-TABQ structure at atomic resolution, revealing a pseudosquare-planar Cu-bis­(iminobenzosemiquinoid) motif with pronounced structural asymmetry and a structural model consistent with possible local protonation near the elongated C–N site. By directly comparing structural analogs, we provide an atomic-level explanation for the long-observed conductivity disparity between Ni- and Cu-coordinated diamine frameworks: Ni-TABQ adopts a planar, symmetric coordination environment that supports delocalized, near-metallic transport, whereas Cu-TABQ exhibits a distorted structure with localized electronic states, yielding semiconducting property and a conductivity suppressed by nearly 6 orders of magnitude. DFT further reveals that protonation stabilizes the distorted Cu-TABQ structure and reproduces its semiconducting electronic properties, whereas Ni-TABQ favors near-metallic charge transport. Overall, this work provides the first atomic-level structural insight into metal-bis­(iminobenzosemiquinoid) motifs in diamine-based frameworks, directly linking coordination symmetry and protonation to charge-transport behavior. More broadly, these results identify metal–ligand conjugation and protonation state as key, tunable parameters for controlling transport regimes in conductive framework materials, providing insight into design strategies for materials with targeted electronic functions in sensing, electronics, and energy-related applications.

## Supplementary Material



## Notes

Details for synthetic conditions, SEM, PXRD test, MicroED analysis, TEM characterizations, crystal information on TABQ ligand, crystal structures and bonding analysis of M-TABQ, FT-IR and Raman spectroscopy, EPR spectroscopy, BET analysis, TGA, XPS, conductivity measurement, magnetic measurement and Hall effect measurements, and computational studies are in Supporting Information (PDF). Crystallographic information for Cu-TABQ determined by MicroED (CIF file) (PDF)
