# Supplementary material for: Modulating the Electronic Properties of Conductive Conjugated Coordination Polymers with Atomic-Level Precision
Source: J Am Chem Soc. 2026 Sep 15;148(38):40733–42. doi: 10.1021/jacs.6c06561 (PMC13632550; doi:10.1021/jacs.6c06561)
Supplement: Supplementary file 1 [file ja6c06561_si_001.pdf]

Supporting Information for

Modulating the electronic properties of conductive conjugated coordination polymers with atomic-level precision

Hyuk-Jun Noh[1], Doran L. Pennington[2], Mark Del Campo[3], Christopher H. Hendon[2]*, and Katherine A. Mirica[1]**

[1] Department of Chemistry, Burke Laboratory, Dartmouth College, Hanover, New Hampshire 03755, United States

[2] Department of Chemistry and Biochemistry, University of Oregon, Eugene, Oregon 97403, United States

[3] Rigaku Americas, The Woodlands, Texas 77381, United States

* Correspondence: hyukjunnoh@gmail.com, chendon@uoregon.edu, katherine.a.mirica@dartmouth.edu;

Content

1. Materials and Methods 3
2. Synthetic procedures 7
3. Scanning electron microscopy 15
4. MicroED for structural characterization of Cu-TABQ 16
5. High-resolution transmission electron microscopy 20
6. Crystal structure of TABQ ligand 22
7. Crystal structures and bonding analysis of M-TABQ 23
8. Raman spectroscopy 26
9. Electron paramagnetic resonance (EPR) spectroscopy 27
10. Magnetic property 28
11. Fourier-transform infrared (FT-IR) spectroscopy 30
12. Thermal gravimetric analyses (TGA) 32
13. Brunauer-Emmett-Teller (BET) analyses 33
14. X-ray photoelectron spectroscopy (XPS) study 35
15. Measurements of electrical conductivity 37
16. Hall effect measurement 41
17. Density functional theory (DFT) 43

1. Materials and Methods

Concentrated ammonium hydroxide (NH_4OH, CAS number 1336-21-6) was purchased from Ward's Science (St. Catharine's, Ontario, Canada). Copper (Ⅱ) acetate anhydrous ($Cu(OAc)_2$, CAS number 142-71-2) and dimethyl sulfoxide (DMSO, CAS number 67-68-5) were purchased from Thermo Fisher Scientific. Tetrachloro-*p*-benzoquinone (CAS number 118-75-2), potassium phthalimide (CAS number 1074-82-4), *N,N*-dimethylformamide (DMF, CAS number 68-12-2), copper (Ⅰ) iodide (7681-65-4), copper powder (CAS number 7440-50-8), copper (II) nitrate hemipentahydrate ($Cu(NO_3)_2 \cdot 2.5H_2O$, CAS number 19004-19-4), hydrazine monohydrate (CAS number 7803-57-8), acetonitrile (CAS number 75-05-8) and sodium acetate (CAS number 127-09-3) were purchased from MilliporeSigma.

Nuclear magnetic resonance (NMR) spectroscopy

NMR spectra were recorded on a 600 MHz NMR spectrometer from Bruker (Billerica, MA).

Powder X-ray (PXRD) measurements and Rietveld refinement

Powder X-ray (PXRD) measurements were carried out using a Rigaku sixth generation MiniFlex X-ray diffractometer with a 600 W (40 kV, 15 mA) $CuK_{\alpha 1}$ (λ = 1.5406 Å) radiation source. The scan rate was 1° min^{-1} and data points were collected every 0.02° in the range of 2θ = 3 to 60°.

The crystal structure of Ni-TABQ was refined against PXRD data using the Reflex module implemented in Materials Studio 2025, employing a Pseudo-Voigt profile function. The refinement was performed using a previously reported structural model as the starting point.[1] The refined lattice parameters of Ni-TABQ at 298 K are a = 12.1315(16) Å, b = 7.6425(31) Å, and c = 3.7671(8) Å, β = 97.323° in the space group C2/m with R_{wp} = 3.18% and R_p = 2.14%.

MicroED/3DED analysis of Cu-TABQ

Some Cu-TABQ powder was crushed between two glass slides to make crystals small enough for microED/3DED. A 3 mm diameter lacey carbon TEM grid was swept through the material. The grid

was then secured in a JEOL high tilt room temperature specimen holder. The specimen holder was inserted into a Rigaku XtaLAB Synergy-ED electron diffractometer equipped with a HyPix-ED detector for MicroED/3DED data collection. The electron optics of the XtaLAB Synergy-ED are based on a 200 kV electron gun with a lanthanum hexaboride crystal as the electron emitter.[2, 3] Each diffraction data set was collected using either a 100- or 50-micron selected area aperture. All instrument operation and data processing used Rigaku CrysAlis^{Pro}.[4] Data sets were collected on multiple crystals and then the data sets were merged to increase completeness (4 data sets). All structures were solved via intrinsic phasing methods using ShelXT[5] and refined with ShelXL[6] within the Olex2 graphical user interface.[7] All non-hydrogen atoms were refined anisotropically. Hydrogen atoms were placed on positive Coulombic difference map peaks near the nitrogen atoms and freely refined to avoid any bias from relying on the structure solution program to place the hydrogens at their idealized positions.

Diffuse reflectance UV-vis-NIR spectra

To measure the UV-vis-NIR spectra of samples, the materials dispersed in H_2O was dropcasted onto the quartz slides. UV-vis-NIR between 300 and 2100 nm were collected on a JASCO V-570 spectrophotometer at the scan rate of 400 nm min^{-1} under ambient conditions. A blank quartz slide baseline and a zero background correction were conducted prior to the sample measurement.

Scanning electron microscopy (SEM)

Scanning electron microscopy images of materials were obtained using a Thermo Scientific Helios 5 CX Dual Beam (Waltham, MA) equipped for X-ray microanalysis with a Bruker Edax light element Si(Li) detector (Billerica, MA). The images were taken at a 5 mm working distance using a 10 kV beam line in a 10^{-6} torr vacuum chamber.

High-resolution transmission electron microscopy (HR-TEM)

HR-TEM was measured using a Thermo Scientific Talos F200i with a Cold Field Emission Gun (X-CFEG) instrument. The sample was prepared by sonicating the CCP samples in acetone for 1 min. The

sample was suspended in acetone and drop-casted onto a 200 copper mesh, lacey carbon grid (Ted Pella) and dried in vacuum oven.

Thermal gravimetric analyses (TGA)

TGA was conducted on a TGA Q5000 V3.17 Build 265 instrument with a 10 °C min^{-1} ramping rate from 30 °C to 800 °C under air and N_2 atmosphere.

Brunauer-Emmett-Teller (BET) analyses

To assess the porosity of sample, nitrogen adsorption isotherm was measured on 3Flex (Micromeritics, Norcross, Georgia) at 77 K. The samples were degassed under vacuum at 100 °C for 18 h to remove trapped moisture and/or solvents.

X-ray photoelectron spectroscopy (XPS)

X-ray photoelectron spectroscopy (XPS) measurements were performed on a Kratos Analytical AXIS Supra X-ray Photoelectron Spectrometer under ultrahigh vacuum (base pressure 10^{-7} Torr) equipped with a monochromatic Al (Kα) X-ray source. Both survey and high-resolution spectra were obtained using a beam diameter of 200 μm. The measurement chamber was equipped with a monochromic Al (Kα) X-ray source. The XPS data were processed with ESCape.

Fourier-transform infrared (FT-IR) spectroscopy

Infrared spectra were collected using a Thermo Scientific Nicolet iS50 FT-IR spectrometer with an attenuated total reflection (ATR) mode.

Raman spectroscopy

The CCPs powder was deposited on a gold substrate for Raman measurement. Raman spectrum was collected at room temperature in LabRAM HR Evolution Raman microscope with laser in the wavelength of 532 nm was used for excitation.

Electron paramagnetic resonance (EPR) spectroscopy

EPR spectra were measured on a Bruker BioSpin Gmbh spectrometer equipped with a standard mode cavity. For each sample, about 2-3 mg of material was used. The samples were purged with N_2 for 2 h and then sealed in the EPR tube under N_2 atmosphere. The EPR spectra were collected under room temperature (298 K) and liquid N_2 temperature (77 K).

2. Synthetic procedures

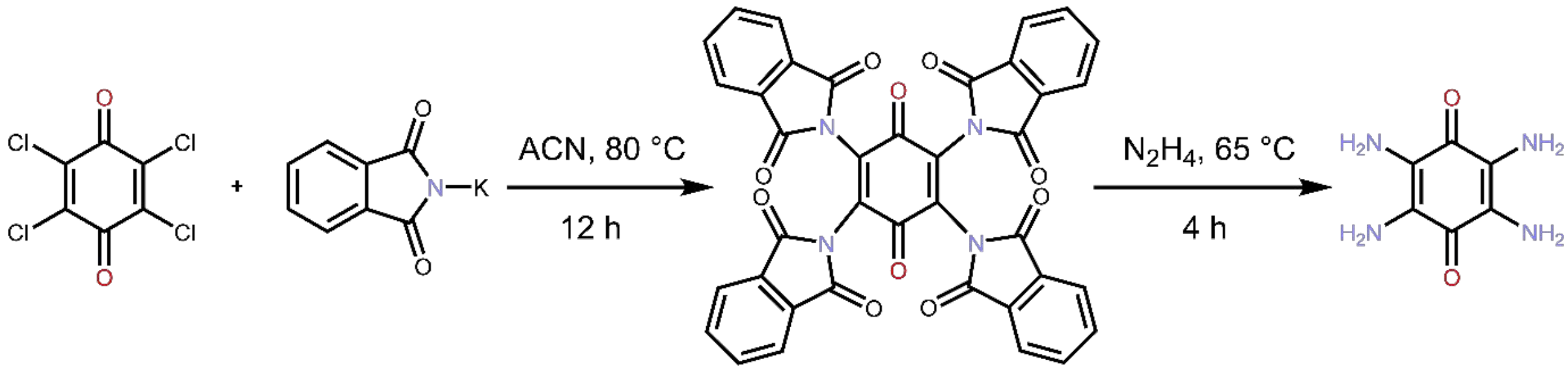

1 2 3 4

Scheme S1. The synthesis procedure of TABQ ligand.

Tetra(phthalimido)-*p*-benzoquinone (TPBQ, 3). To a stirred solution of tetrachloro-*p*-benzoquinone (**1**, 25 g, 101.68 mmol) in 250 mL acetonitrile, potassium phthalimide (**2**, 75 g, 404.92 mmol) was added. The reaction mixture was heated at 80 °C and refluxed for 12 hours under N_2 atmosphere. After cooling to room temperature, the mixture was filtered, and the resulting solid was washed with 300 mL of acetonitrile. The crude solid was then suspended in 100 mL of dimethylformamide (DMF) and heated to 100 °C with stirring for 30 min. The hot suspension was filtered to afford a brown solid, which was subsequently suspended in a DMF/ethanol (800 mL, v/v = 1:1), and sonicated for 1 hour. The mixture was filtered, and the solid was washed thoroughly with DMF, DI water, and hot EtOH to afford a light brown solid in quantitative yield (69.0 g, 98.5 %). Due to the limited solubility of TPBQ, NMR spectroscopy could not be performed.

Tetraamino-*p*-benzoquinone (TABQ, 4). To a 250 mL round bottom flask, 10 g of TPBQ (**3**, 14.523 mmol) was added, followed by the addition of 40 mL of hydrazine hydrate (79 wt%). The reaction mixture was heated to 65 °C and stirred vigorously for 4 hours. After cooling to room temperature, the mixture was filtered, and the resulting solid was washed thoroughly with DI water until the filtrate became colorless and pH-neutral. The resulting deep purple crystalline solid was dried at 70 °C under reduced pressure, affording 66.3 % (1.62 g) of isolated yield. Anal. Cald. For $C_6H_8N_4O_2$: ^{1}H NMR (600 MHz, DMSO-d_6); δ (ppm) 4.54 (s, 8H, amine *H*). ^{13}C NMR (100 MHz, DMSO-d_6); δ (ppm) 178.85, 121.37.

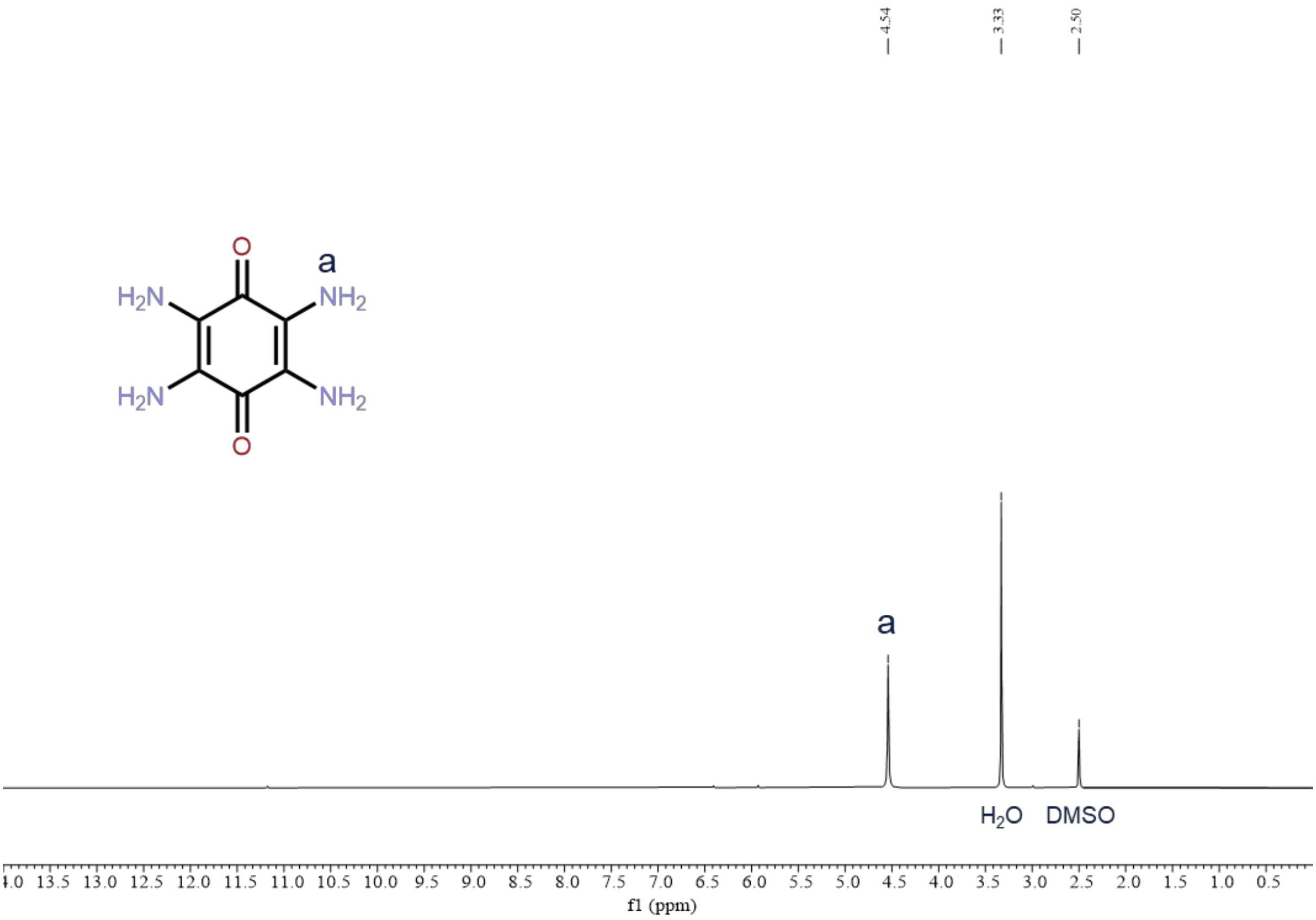

Figure S1. ^{1}H NMR spectrum of compound **4**.

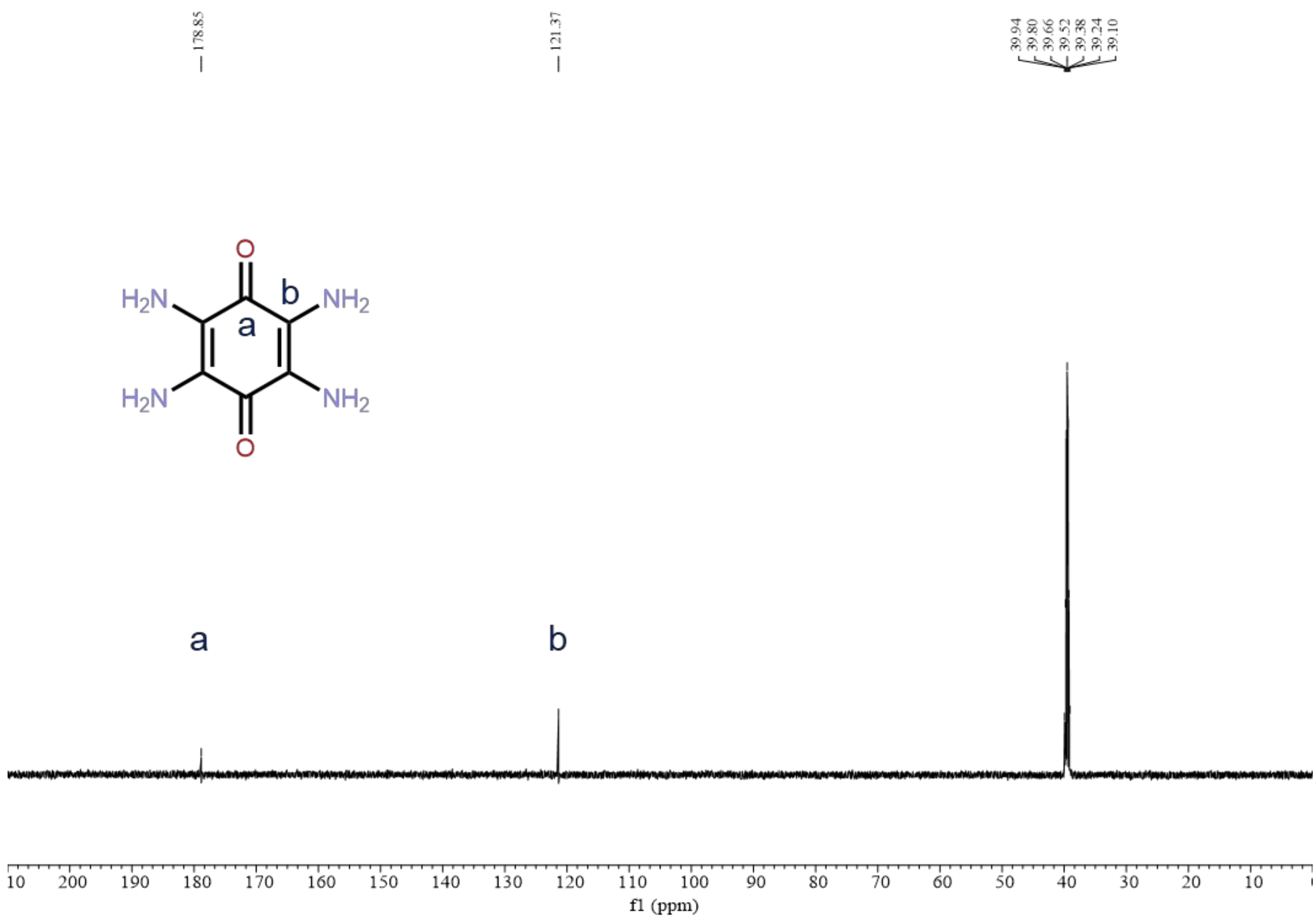

Figure S2. ^{13}C NMR spectrum of compound **4**.

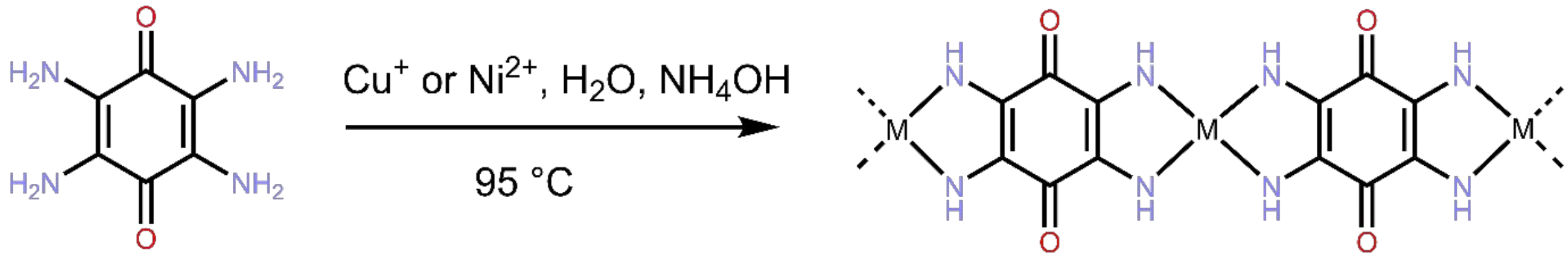

Figure S3. Schematic presentation of the M-TABQ (M: Cu and Ni).

Synthesis of Cu-TABQ (*N.O.* 1, optimized synthetic condition). Under ambient condition, TABQ (16.8 mg, 0.1 mmol) and CuI (38.1 mg, 0.2 mmol) were transferred into a 15 mL thick-wall pressure tube, followed by the addition of degassed DI water (0.4 mL) and concentrated ammonium hydroxide solution (4.6 mL). The pressure tube, sealed under air atmosphere, was heated at 95 °C in an isothermal oven for 24 h. After cooling down to room temperature, the resulting dark navy crystalline precipitate was collected by filtration under ambient conditions and thoroughly washed with DI water, methanol, and acetone. The powder was then dried in a vacuum oven at 70 °C overnight.

Our findings through the synthetic optimizations for Cu-TABQ could be summarized as follows. (1) The use of Cu^{+} precursor, such as CuI, resulted in higher crystallinity compared to Cu^{2+} precursors, including $Cu(NO_3)_2$ and $Cu(OAc)_2$, as well as Cu^0 precursors (**N.O. 1, 2, 3, 4** in **Table S1**). This enhanced crystallinity is likely due to partial oxidation of Cu^{+} to Cu^{2+} during the solvothermal reaction, which may slow the coordination kinetics and promote the growth of larger crystallites relative to those obtained from direct Cu^{2+} sources. In contrast, Cu^0 powder exhibited very poor solubility in the reaction solvent systems, making its oxidation to Cu^{2+} less favorable and thus further limiting its effectiveness as a precursor. (2) The use of a high ratio (9:1) mixture of conc. ammonium hydroxide (NH_4OH) and water was crucial for obtaining crystalline materials. (**N.O. 1, 5, 7, 8, 9, 10** in **Table S1**). This is because conc. NH_4OH effectively solubilizes the Cu ion sources, and while water improves the solubility of the TABQ ligand. (3) Likewise, using water alone without any base, or using conc. NH_4OH without water (**N.O. 1, 6, 11** in **Table S1**), resulted in the crystallization of the free TABQ ligand or the formation of

a different crystal structure, as previously reported.[8] These outcomes are attributed to the differential solubility of the Cu precursors and TABQ ligands in conc. NH_4OH and water, respectively.

Synthesis of Ni-TABQ.[1] Under ambient condition, TABQ (16.8 mg, 0.1 mmol) and $Ni(OAc)_2 \cdot 4H_2O$ (49.8 mg, 0.2 mmol) were transferred into a 15 mL thick-wall pressure tube, followed by the addition of degassed DI water (0.4 mL) and concentrated ammonium hydroxide solution (2.0 mL). The pressure tube, sealed under air atmosphere, was heated at 95 °C in an isothermal oven for 2 days. After cooling down to room temperature, the resulting black crystalline precipitate was collected by filtration under ambient conditions and thoroughly washed with DI water, methanol, and acetone. The powder was then dried in a vacuum oven at 70 °C overnight.

Table S1. Summary of the synthetic conditions of Cu-TABQ

No.	Solvents (v/v)	Ligand Conc.	Additive (e.q.)	CuL_2 (e.q.)	Time (h) /Temp. (°C)
**1**	**H_2O/NH_4OH (1:9)**	**0.1 mmol/5 mL**	**Conc. NH_4OH**	**CuI (2.0)**	**24/95**
2	H_2O/NH_4OH (1:9)	0.1 mmol/5 mL	Conc. NH_4OH	$Cu(NO_3)_2$ (2.0)	24/95
3	H_2O/NH_4OH (1:9)	0.1 mmol/5 mL	Conc. NH_4OH	Cu, powder (2.0)	24/95
4	H_2O/NH_4OH (1:9)	0.1 mmol/5 mL	Conc. NH_4OH	$Cu(OAc)_2$ (2.0)	24/95
5	H_2O	0.1 mmol/5 mL	NaOH (20)	CuI (2.0)	24/95
6	NH_4OH	0.1 mmol/5 mL	Conc. NH_4OH	CuI (2.0)	24/95
7	H_2O	0.1 mmol/5 mL	CH_3COONH_4 (20)	$Cu(NO_3)_2$ (2.0)	24/95
8	H_2O	0.1 mmol/5 mL	CH_3COOK (20)	$Cu(NO_3)_2$ (2.0)	24/95
9	H_2O/NH_4OH (4:1)	0.1 mmol/5 mL	Conc. NH_4OH	$Cu(NO_3)_2$ (2.0)	24/95
10	$DMSO/NH_4OH$ (3:7)	0.1 mmol/5 mL	Conc. NH_4OH	$Cu(NO_3)_2$ (2.0)	24/95
11	H_2O	0.1 mmol/5 mL	-	$Cu(NO_3)_2$ (2.0)	24/95

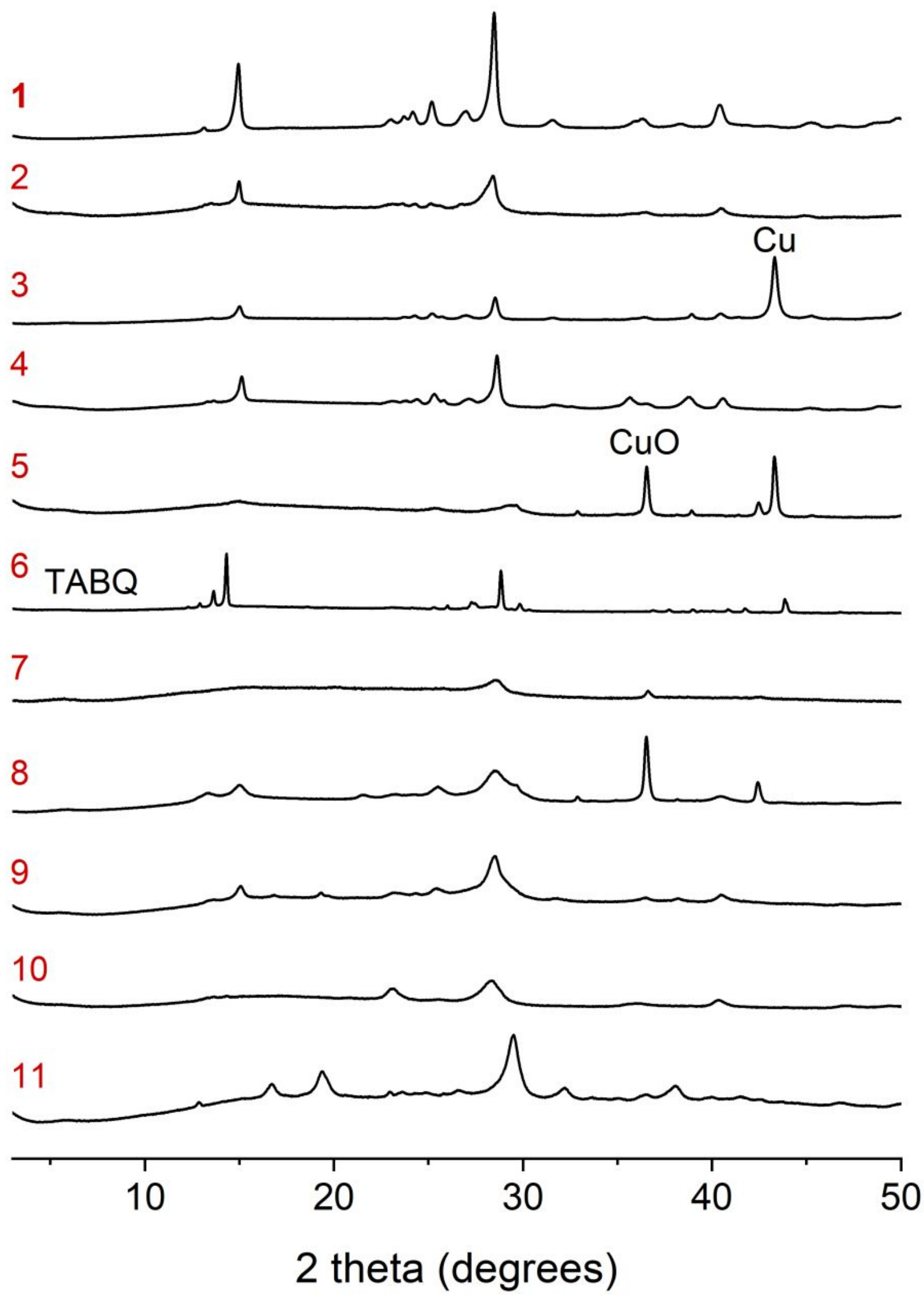

Figure S4. Comparison of PXRD of Cu-TABQ synthesized from conditions in **Table S1**.

3. Scanning electron microscopy

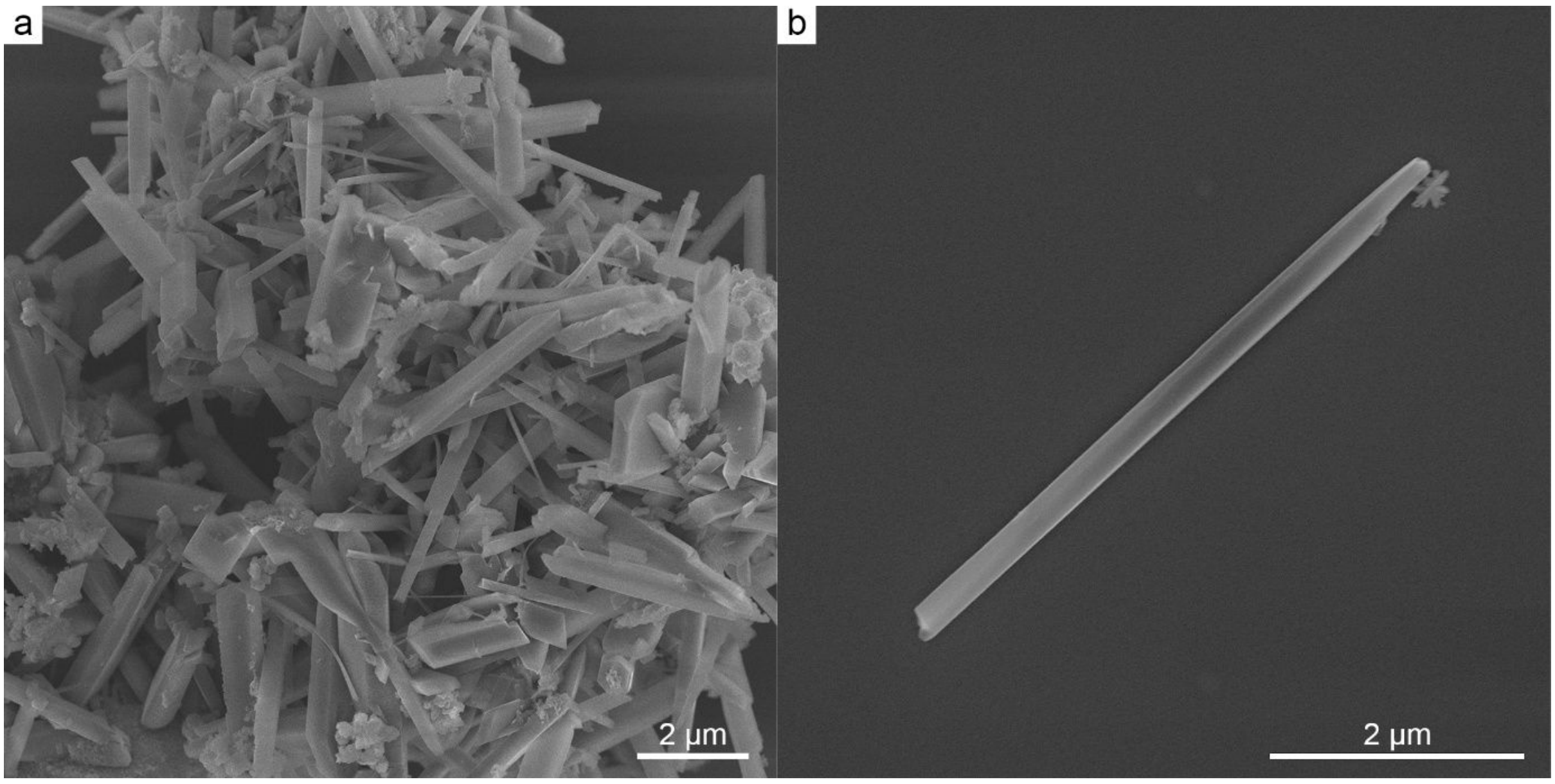

Figure S5. Scanning electron microscope (SEM) images of (**a**) microcrystals and (**b**) dropcasted single crystal of Cu-TABQ, synthesized using the optimized condition (**entry 1** in **Table S1**) in this work.

4. MicroED for structural characterization of Cu-TABQ

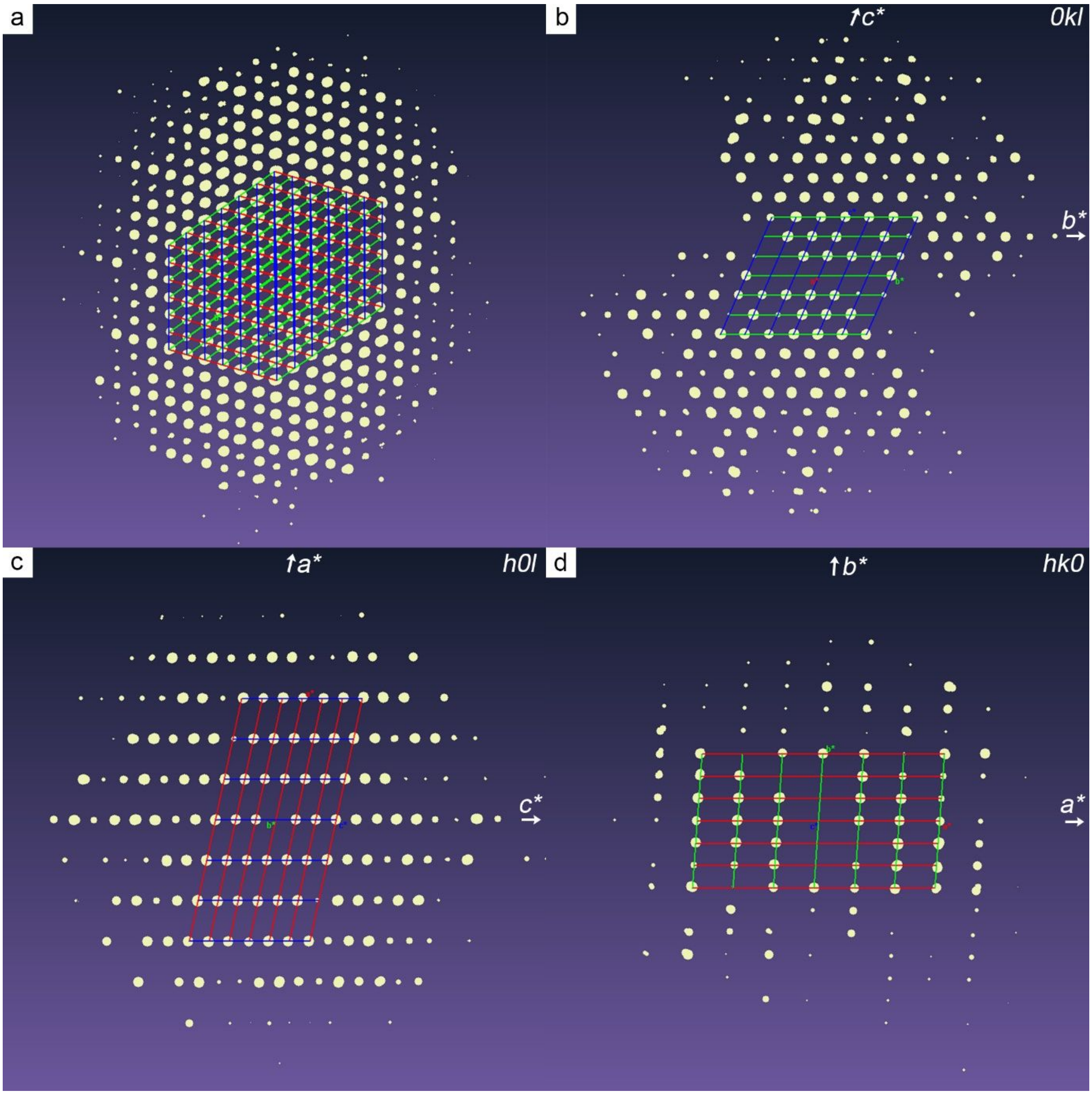

Figure S6. Reconstructed 3D reciprocal lattice of Cu-TABQ with a maximum resolution of 0.6Å: (**a**) overview (*hkl*), and selected planes in the reciprocal lattice corresponding to (**b**) *0kl*, (**c**) *h0l*, and (**d**) *hk0*.

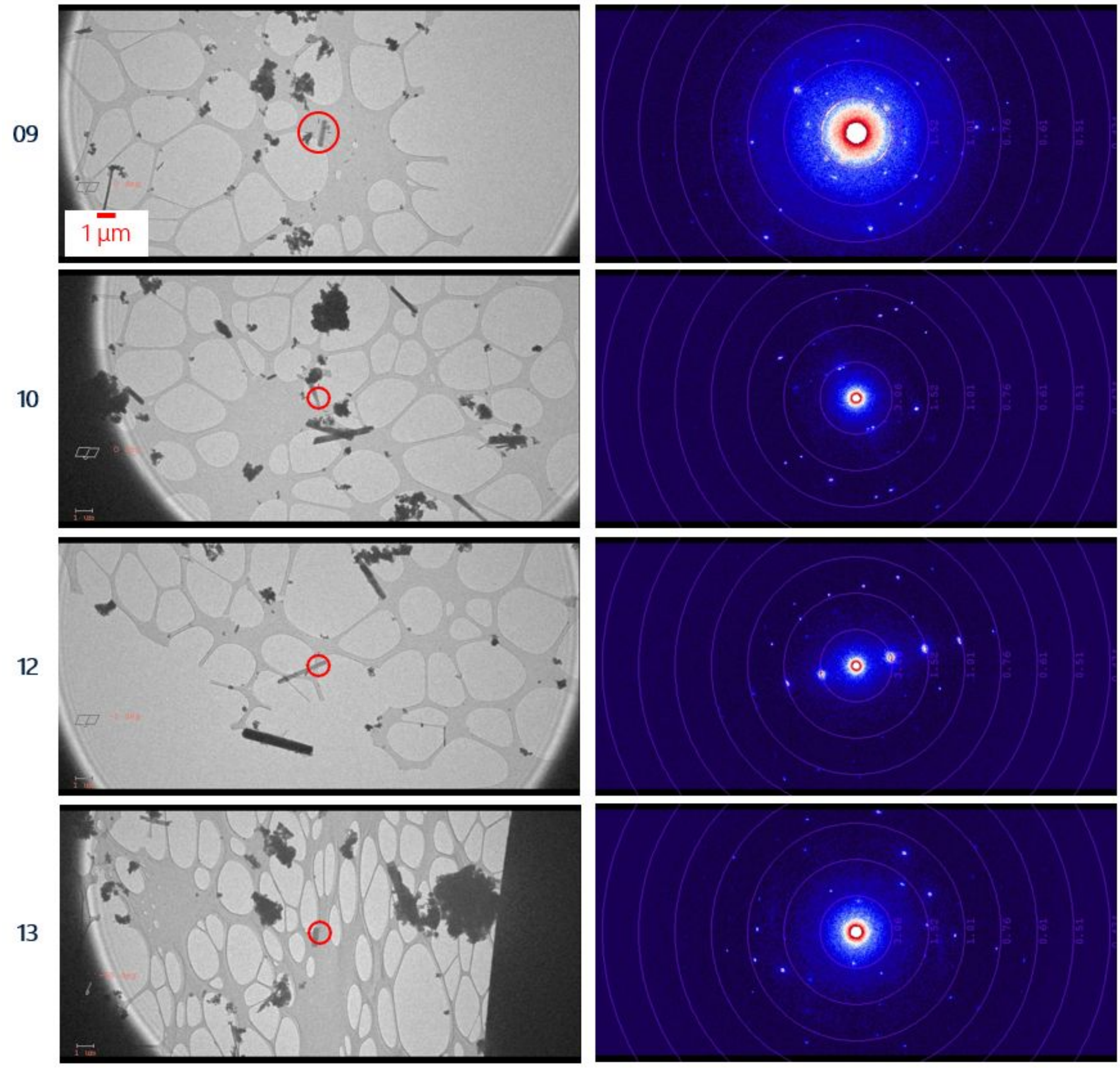

Figure S7. MicroED images from the XtaLAB Synergy-ED. Left, defocused diffraction images of Cu-TABQ crystals. A 2 or 1 µm circle is shown around the centered crystal to indicate the position of the selected area aperture (100 or 50 µm, respectively) used in diffraction mode to only collect data from that crystal. Right, representative diffraction images from crystals on left with resolution rings indicated.

Table S2. Crystal data collection and crystallographic data for Cu-TABQ

Cu-TABQ	
Empirical formula	$C_6H_6CuN_4O_2$
Formula weight	229.69
Temperature/K	298
Crystal system	Triclinic
Space group	*P-1*
a/Å	3.6540(5)
b/Å	6.6455(12)
c/Å	7.5913(8)
α/°	113.749(14)
β/°	102.798(11)
γ/°	93.659(13)
Volume/Å^3	162.10(5)
Z	1
ρ_{calc}g/cm^3	2.353
F(000)	37.0
Radiation	Electron (λ = 0.0251 Å)
Completeness	92.5%
Resolution	0.6 Å
Dataset (*h*, *k*, *l*)	$-6 \leq h \leq 6, -11 \leq k \leq \sim 11, -12 \leq l \leq \sim 12$
Reflections collected	5345
Independent reflections	1437 [R_{int} = 0.0819]
Data/restraints/parameters	1437/0/74
Goodness-of-fit on F^2	1.923
Final R indexes [I>=2σ (I)]	R_1 = 0.1679, wR_2 = 0.4534
Final R indexes [all data]	R_1 = 0.1925, wR_2 = 0.4805
Largest diff. peak/hole	0.32/-0.47 e^- Å^{-3}

Table S3. Information on individual microED/3DED data sets

Data set name	09	10	12	13
Exposure time per image	0.4 s	0.4 s	0.4 s	0.4 s
Rotation per image	0.2°	0.2°	0.2°	0.2°
Total rotation range	98°	100°	120°	125°
Total dose time	3 m 16 s	3 m 20 s	4 m 0 s	4 m 10 s
Crystal-to-detector distance	652.5 mm	652.5 mm	652.5 mm	652.5 mm
Resolution	0.6 Å	0.6 Å	0.6 Å	0.6 Å
Completeness[a]	47.3%	48.2%	58.9%	59.1%
I/σI (highest shell)	17.8 (5.3)	16.8 (3.3)	11.6 (2.0)	12.8 (3.1)
R_{int} (highest shell)	4.1% (18.6%)	3.3% (18.5%)	3.5% (34.8%)	7.0% (39.1%)

Highest shell is 0.63 to 0.6 Å

[a] When all 4 data sets were merged, the completeness increased to 93%

5. High-resolution transmission electron microscopy

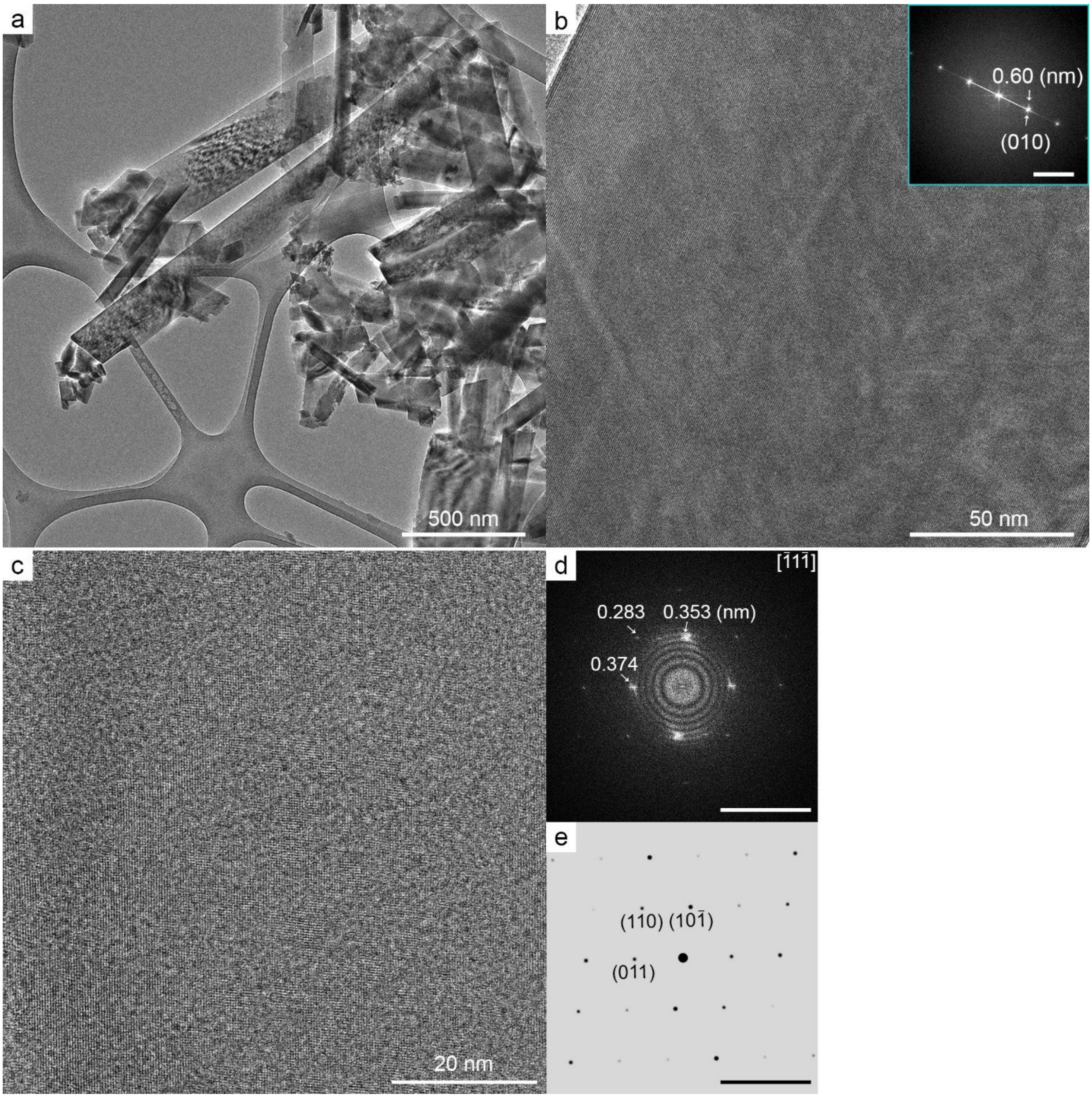

Figure S8. (**a**) Low-magnification transmission electron microscope (TEM) image showing rod-shaped Cu-TABQ crystallites with lengths of several micrometer. (**b**) High-magnification TEM image of Cu-TABQ. Inset: corresponding FFT image, displaying the (010) Bragg reflection. Scale bar: 2 nm^{-1}. (**c**) HR-TEM image of Cu-TABQ observed along the $[\bar{1}1\bar{1}]$ direction. (**d**) Corresponding and (**e**) simulated FFT images, displaying well-defined parallelogram-shaped lattice and diffraction patterns. Scale bars: 5 nm^{-1}.

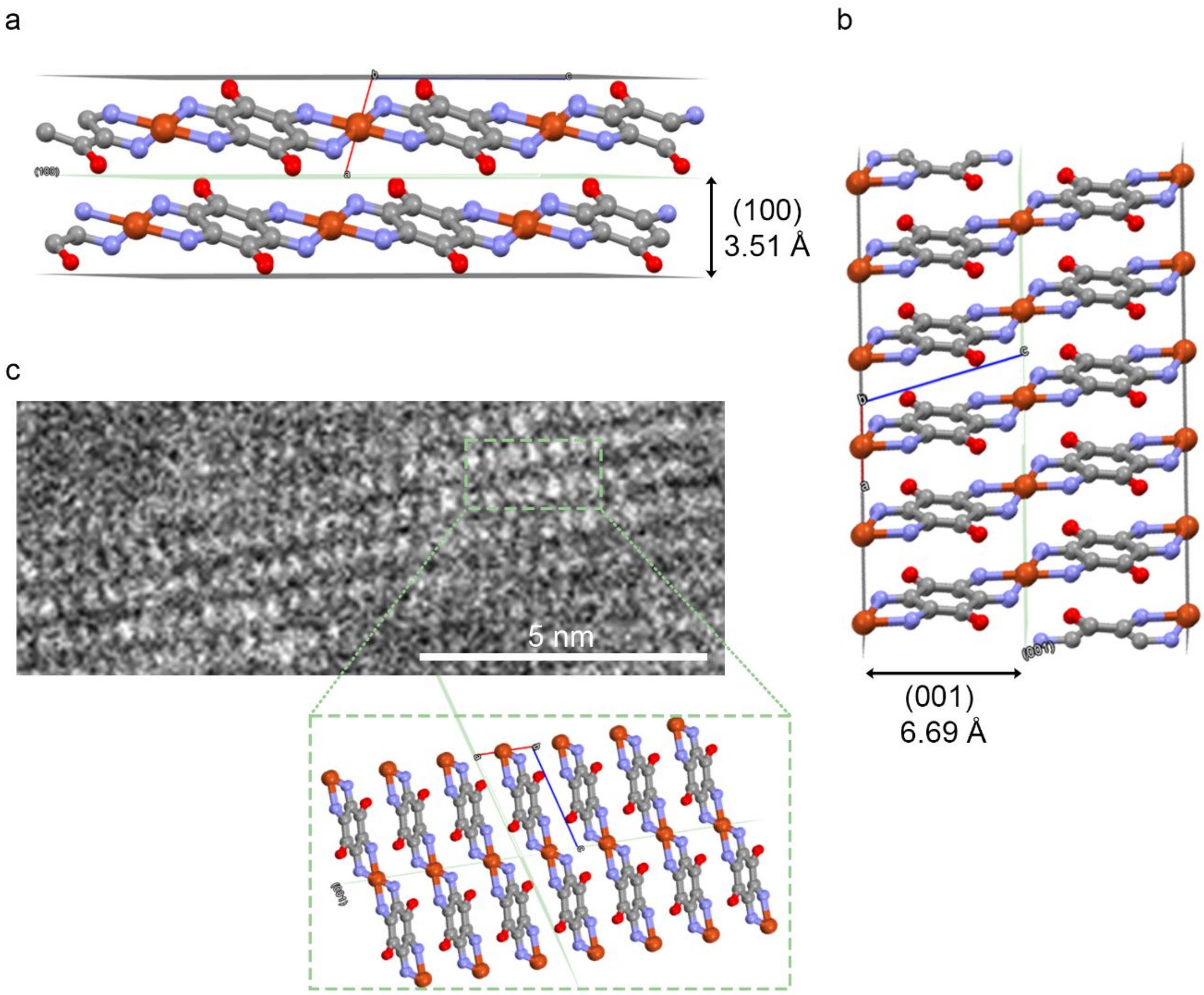

Figure S9. Structural visualizations of the (**a**) (100) and (**b**) (001) crystal planes with indicated interatomic distances. (**c**) Magnified HR-TEM image from **Figure 2f** with the corresponding crystallographic orientation of the Cu-TABQ structure.

6. Crystal structure of TABQ ligand

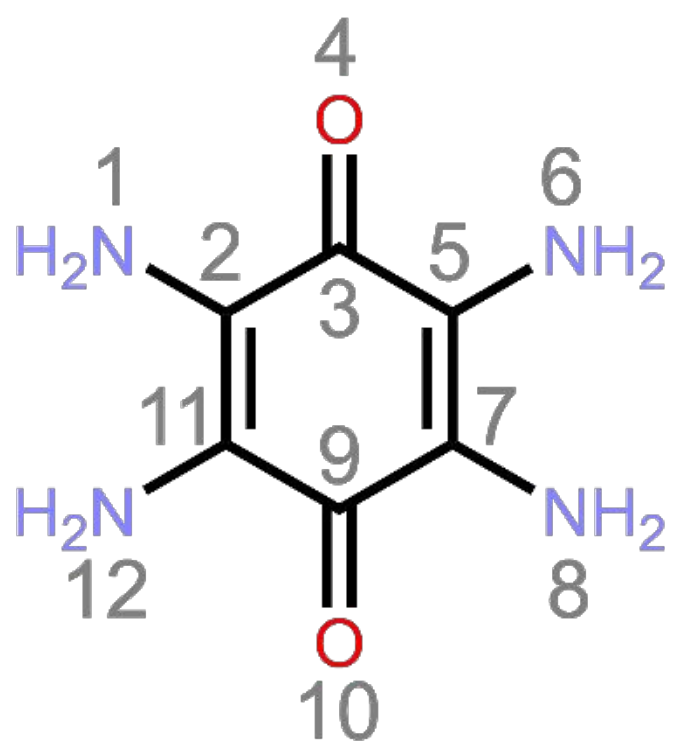

Figure S10. Molecular structure and atom numbering of TABQ.

Table S4 Crystal information and bond lengths of TABQ

Bond length (Å)		Bond order
N1-C2	1.416	1.08
C2-C3	1.442	1.11
C3-O4	1.237	1.87
C3-C5	1.507	0.99
C5-N6	1.351	1.19
C5-C7	1.360	1.47
C7-N8	1.416	1.08
C7-C9	1.442	1.11
C9-O10	1.237	1.87
C9-C11	1.507	0.99
C11-N12	1.351	1.19
C2-C11	1.360	1.47

The bond lengths are derived from the TABQ single-crystal structure (CCDC 1409346),[9] and the bond orders are referenced from a previous report.[1]

7. Crystal structures and bonding analysis of M-TABQ

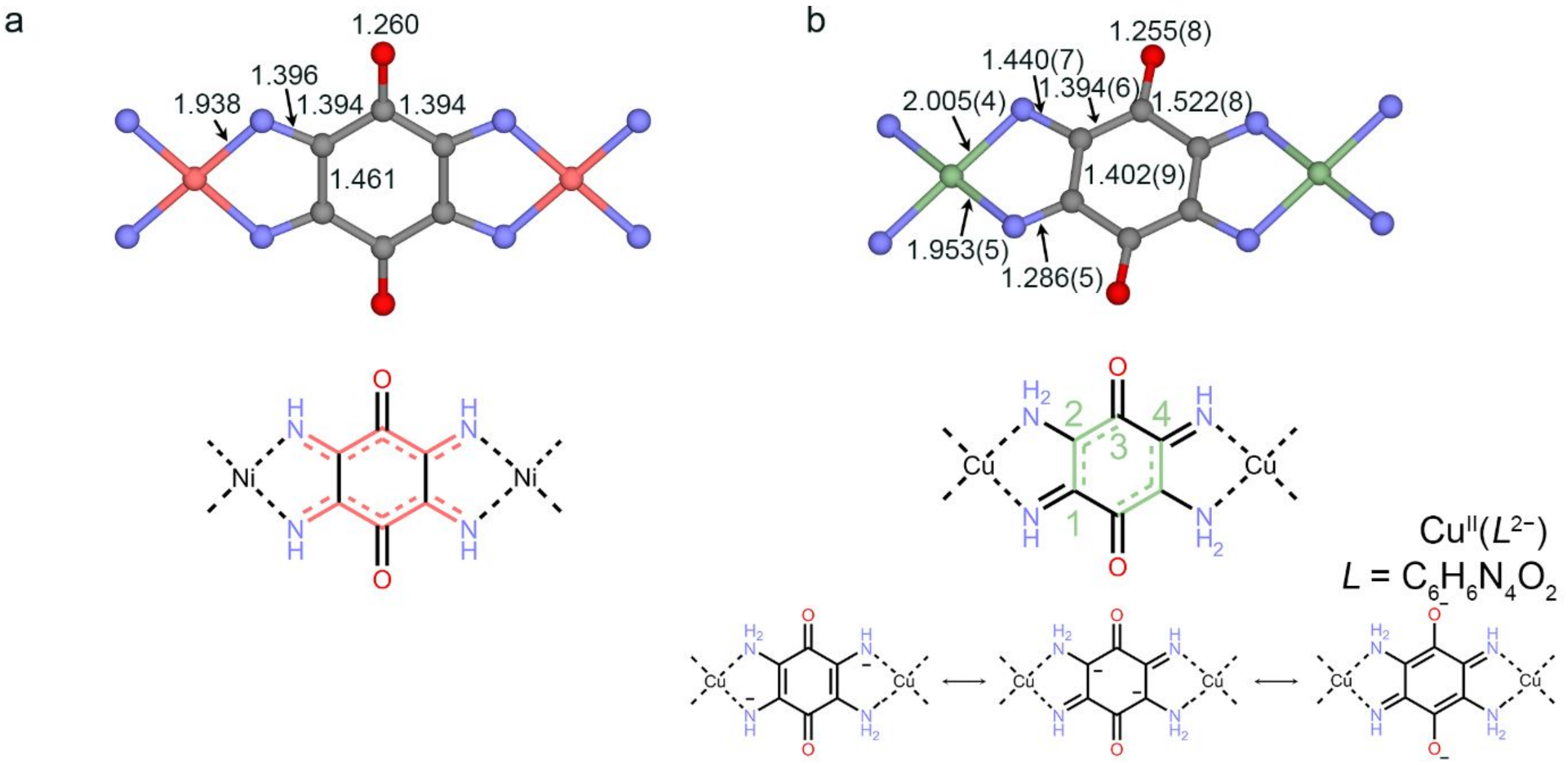

Figure S11. Selected experimental bond lengths (Å) and corresponding structural representations of (**a**) Ni-TABQ and (**b**) Cu-TABQ. The lower Cu-TABQ schematics illustrate the proposed N-bound protonation pattern and representative localized resonance contributors of L^{2-}, with the formal negative charges localized at the N, ring C, or O sites ($L = C_6H_6N_4O_2$). $Cu^{II}(L^{2-})$ is shown as a charge-balanced limiting formulation rather than a unique charge-localized Lewis structure. Hydrogen atoms are omitted from the upper structural models for clarity.

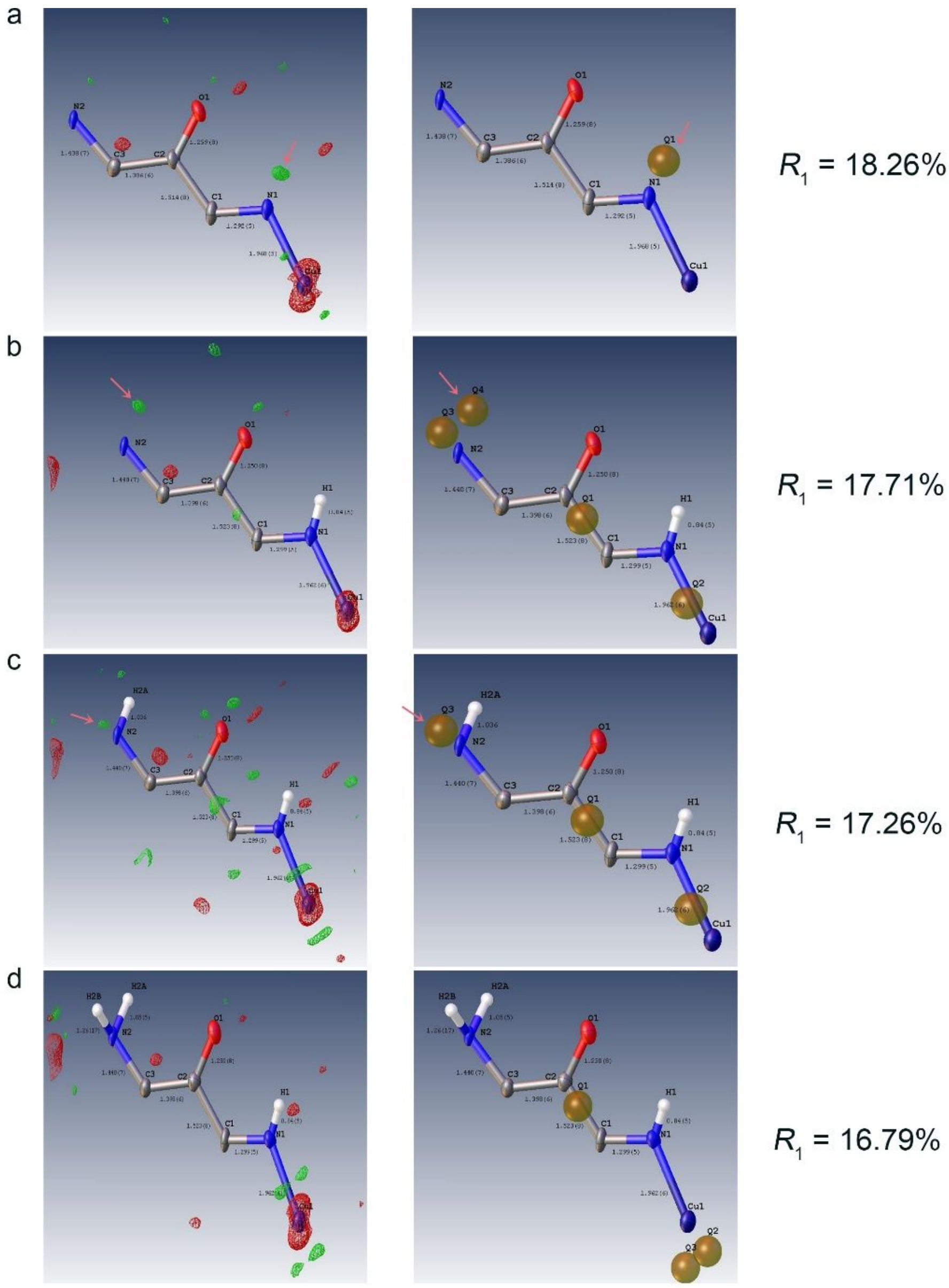

Figure S12. Coulombic/difference map analysis supporting the placement of N-bound H atoms in Cu-TABQ. In each panel, the left image shows the Coulombic/difference map and the right image shows the corresponding q peaks before addition of the indicated H atom. H atoms were sequentially placed at the positive q peaks indicated by arrows and refined. The refinement improved upon sequential H-atom addition, with R_1 decreasing from (**a**) 18.26% for the initial model without H atoms to (**b**) 17.71% after addition of H1, (**c**) 17.26% after addition of H1 and H2A, and (**d**) 16.79% after addition of H1, H2A, and H2B.

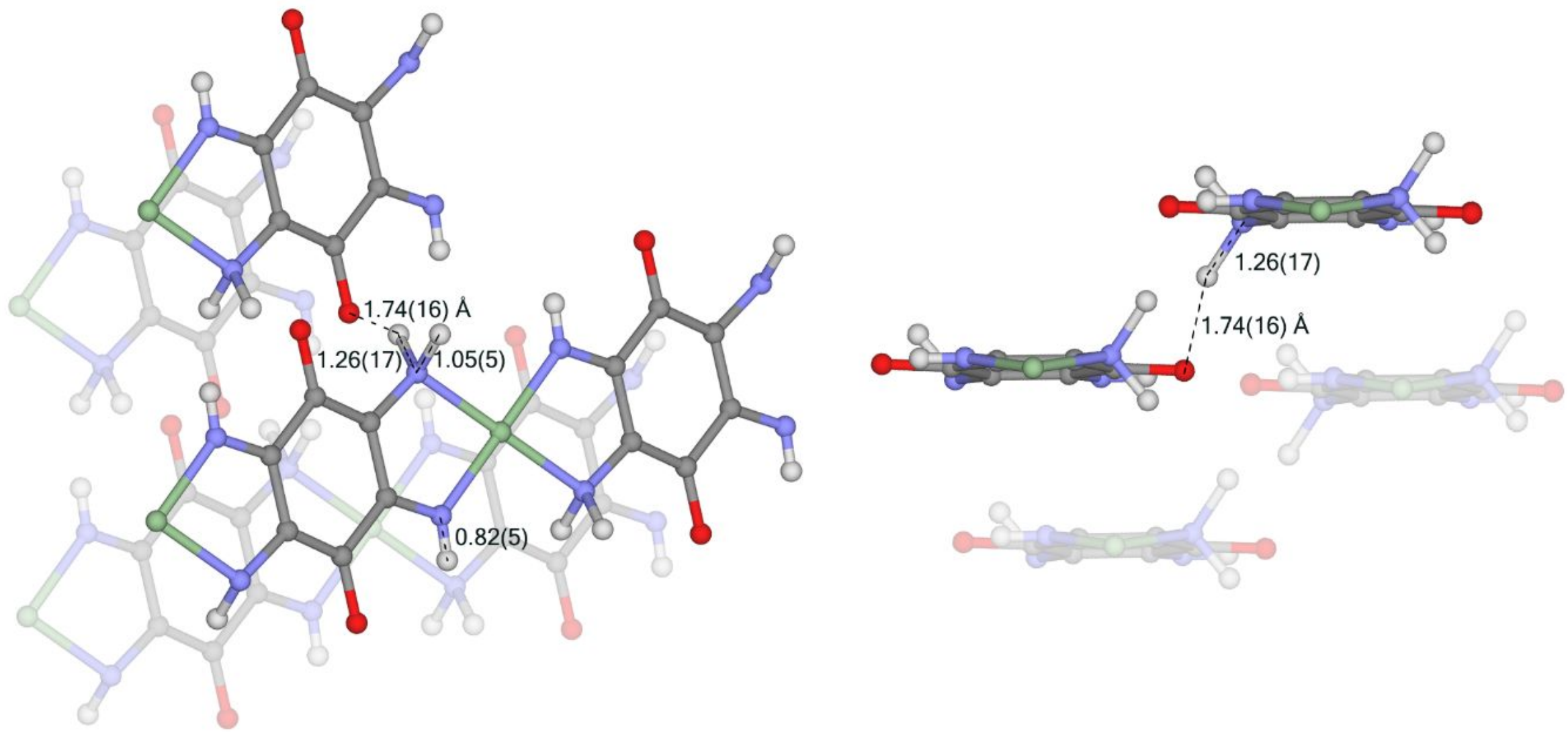

Figure S13. Hydrogen-bonding environment in the MicroED-resolved structure of Cu-TABQ.

The MicroED structure of Cu-TABQ shows an additional hydrogen atom on the nitrogen associated with the elongated C-N bond, forming a local hydrogen-bonding/protonation motif at the asymmetric C-N site. This hydrogen atom is oriented toward a neighboring carbonyl oxygen and participates in an intermolecular N-H···O hydrogen-bonding interaction, with N-H and H···O distances of 1.26(17) and 1.74(16) Å, respectively. The N-H distance associated with this hydrogen-bonding interaction is longer than the other N-H bond of 1.05(5) Å, consistent with elongation of the N-H bond upon involvement in intermolecular hydrogen bonding. Although hydrogen positions determined by MicroED should be interpreted with appropriate caution due to their relatively large uncertainties, particularly in hydrogen-bonded environments, the observed geometry supports a localized N-H···O hydrogen-bonding interaction at the asymmetric C–N site.

As shown in the expanded and side-view representations, this hydrogen-bonding interaction links neighboring Cu-TABQ chains in an inclined geometry. This local hydrogen-bonding/protonation motif is consistent with the asymmetric bonding environment of the Cu-bis(iminobenzosemiquinoid) units and likely contributes to the wavy, non-planar arrangement of the 1D polymeric chains.

8. Raman spectroscopy

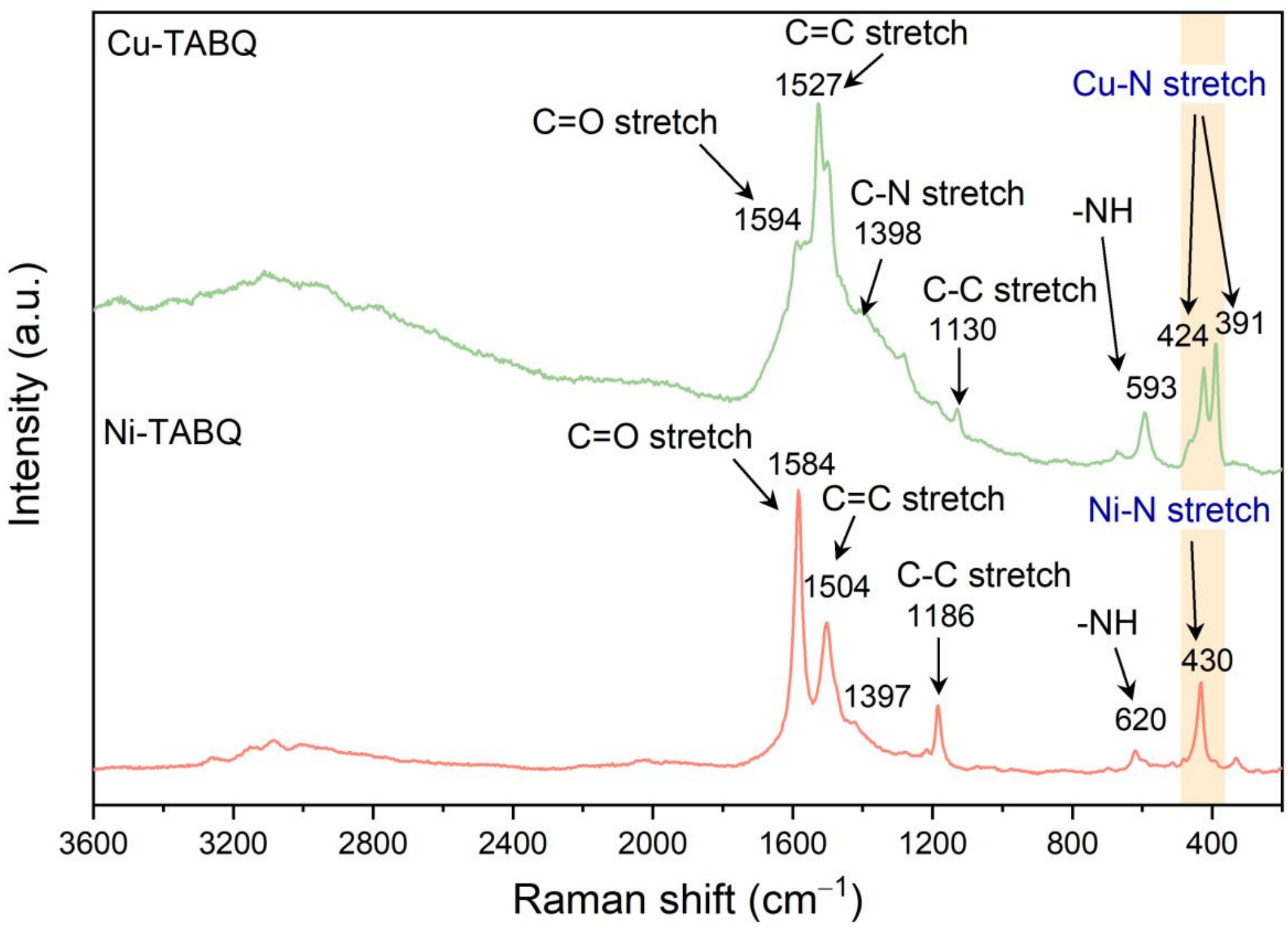

Figure S14. Raman spectra of Cu-TABQ and Ni-TABQ in the range of 200-3600 cm^{-1} using 532 nm laser excitation.

9. Electron paramagnetic resonance (EPR) spectroscopy

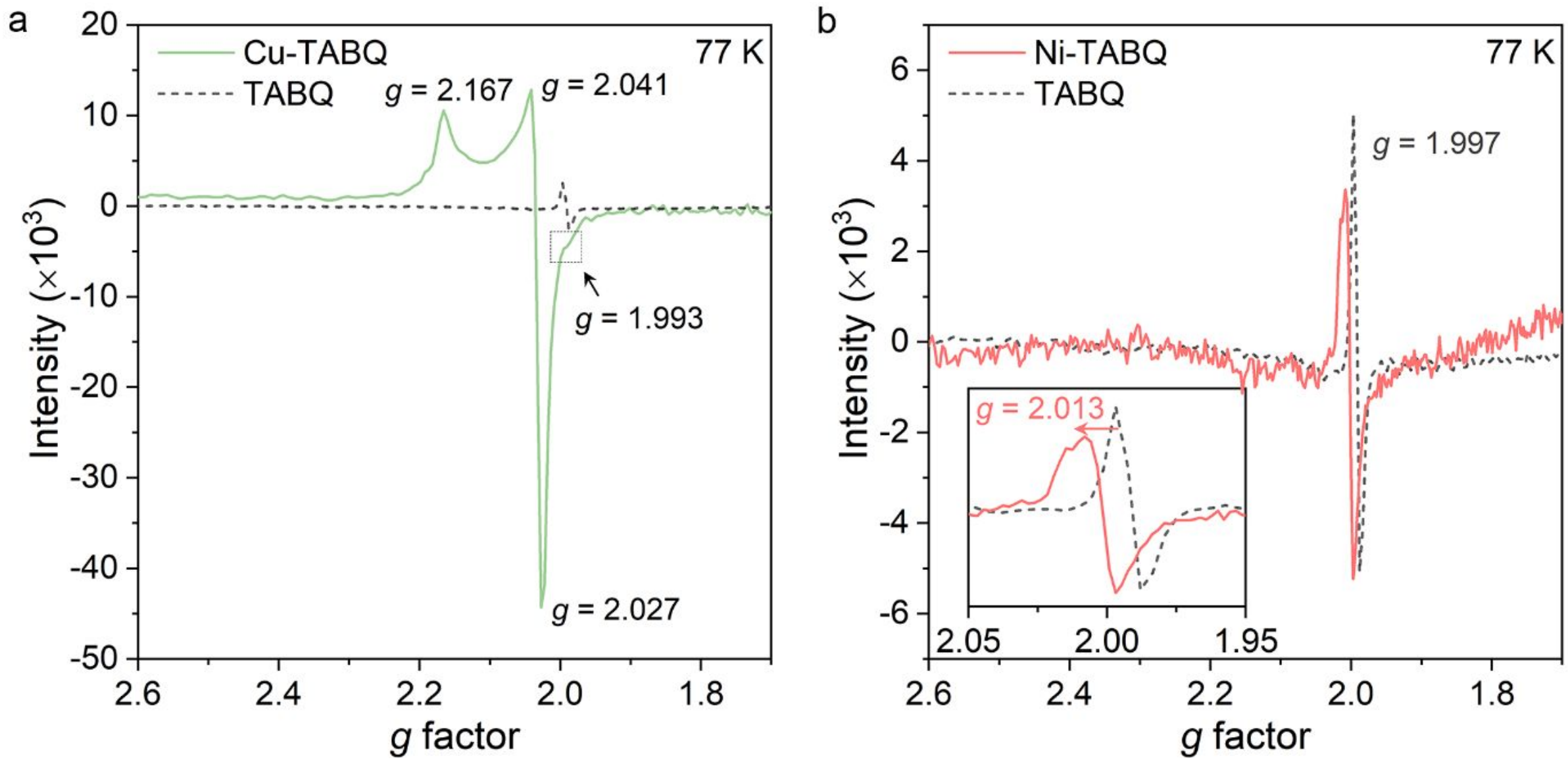

Figure S15. EPR spectra of (**a**) Cu-TABQ and (**b**) Ni-TABQ measured at 77 K, with the TABQ spectrum (gray dashed line) shown as a reference. Cu-TABQ shows an anisotropic EPR response consistent with Cu^{2+}-associated paramagnetic centers, together with a very weak feature near $g = 1.993$ that may correspond to a minor ligand-associated radical contribution. The weak and slightly shifted EPR signal of Ni-TABQ ($g = 2.013$), compared to that of the TABQ ligand ($g \sim 1.997$), indicates that the unpaired spin is not fully localized on the ligand but involves partial contribution from Ni *d* orbitals, consistent with strong metal–ligand orbital coupling.

10. Magnetic property

The magnetic properties of Cu-TABQ were collected on microcrystalline samples with a liquid He cryostat of a Quantum Design PPMS Dynacool magnetometer (Physical Property Measuring System). DC susceptibility measurements were performed under 500 Oe external field in temperature range of 2–300 K. Experimental data were corrected for diamagnetic contribution based on blank sample holder measurements.

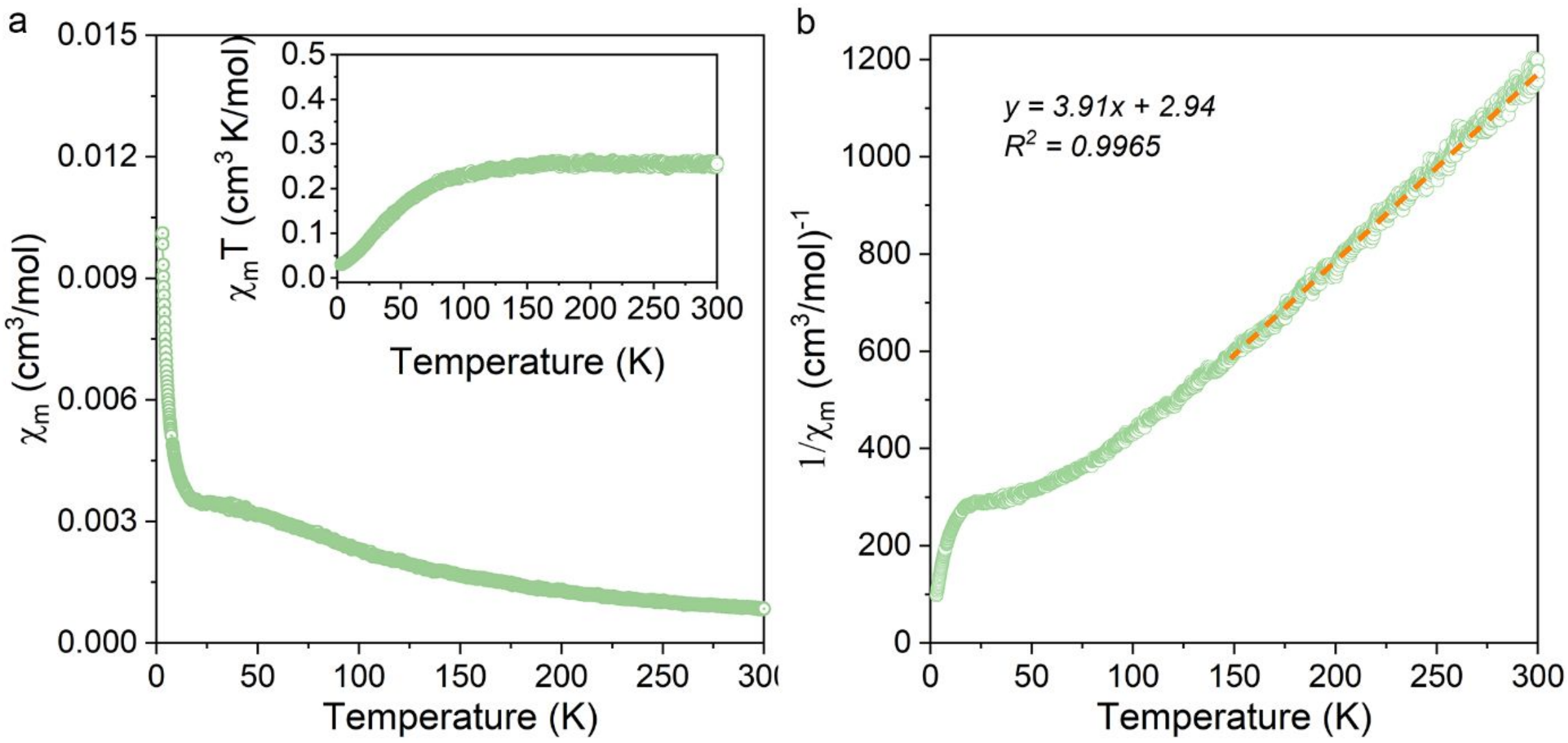

Figure S16. Temperature dependence of (**a**) χ_M, $\chi_M T$, and (**b**) χ_M^{-1} (H = 500 Oe) for Cu-TABQ.

Temperature-dependent magnetic susceptibility measurements were performed on Cu-TABQ to further probe its spin state and magnetic interactions (**Figure S16**). The χₘT value increases with temperature and reaches ~0.25 cm^3 K mol^{-1} at 298 K, which is significantly lower than the spin-only value expected for isolated Cu^{2+} centers (S = 1/2, ~0.375 cm^3 K mol^{-1}).[10] This reduced value indicates that the apparent spin population in Cu-TABQ is smaller than that expected for fully independent Cu^{2+} centers.

Curie-Weiss fitting of the high-temperature region (150–300 K) yields a Curie constant of C = 0.255 cm^3 K mol^{-1} and a Weiss temperature of $\theta = -0.75$ K (**Figure S16b**). The small negative Weiss temperature indicates the presence of antiferromagnetic interactions,[11] while its small magnitude

suggests weak exchange coupling. However, this weak interaction alone is not sufficient to account for the reduced Curie constant. Based on the Cu^{2+}/Cu^{+} ratio estimated from XPS (78:22), approximately 22% of the Cu sites are diamagnetic Cu^{+}. This Cu^{+} fraction lowers the expected Curie constant from 0.375 to approximately 0.293 $cm^3\ K\ mol^{-1}$, accounting for a substantial portion of the experimentally observed reduction. The remaining difference between this Cu^{2+}/Cu^{+}-corrected value and the fitted Curie constant of 0.255 $cm^3\ K\ mol^{-1}$ may arise from a combination of uncertainty in the Cu^{2+}/Cu^{+} ratio, trapped guest molecules or defects, diamagnetic correction, and partial spin quenching through weak antiferromagnetic interactions or Cu–ligand exchange coupling. Because an additional population of free, noninteracting ligand radicals would be expected to increase the Curie constant, the reduced Curie constant is more consistent with a smaller apparent Cu^{2+} spin population and partial spin quenching than with a substantial contribution from independent ligand radicals. This interpretation is consistent with the EPR spectrum of Cu-TABQ (**Figure S15**), which shows only a very weak feature near $g \approx 1.993$ and does not support a substantial population of free, noninteracting ligand-radical spins. If ligand-associated spin density is present, its contribution is therefore likely minor and/or partially exchange-coupled to Cu centers rather than behaving as independent ligand-radical spins. For comparison, prior studies on structurally analogous Ni–diamine systems have shown that square-planar Ni^{2+} adopts a closed-shell $S = 0$ configuration, with magnetic responses arising from ligand-centered radicals coupled through the metal center.[1, 12] Taken together, these results suggest that Cu-TABQ exhibits weak antiferromagnetic coupling with partially quenched paramagnetic Cu^{2+} centers. In contrast, Ni-TABQ is more appropriately described as a ligand-centered, electronically delocalized system, where the magnetic behavior is dominated by ligand-centered radicals.

11. Fourier-transform infrared (FT-IR) spectroscopy

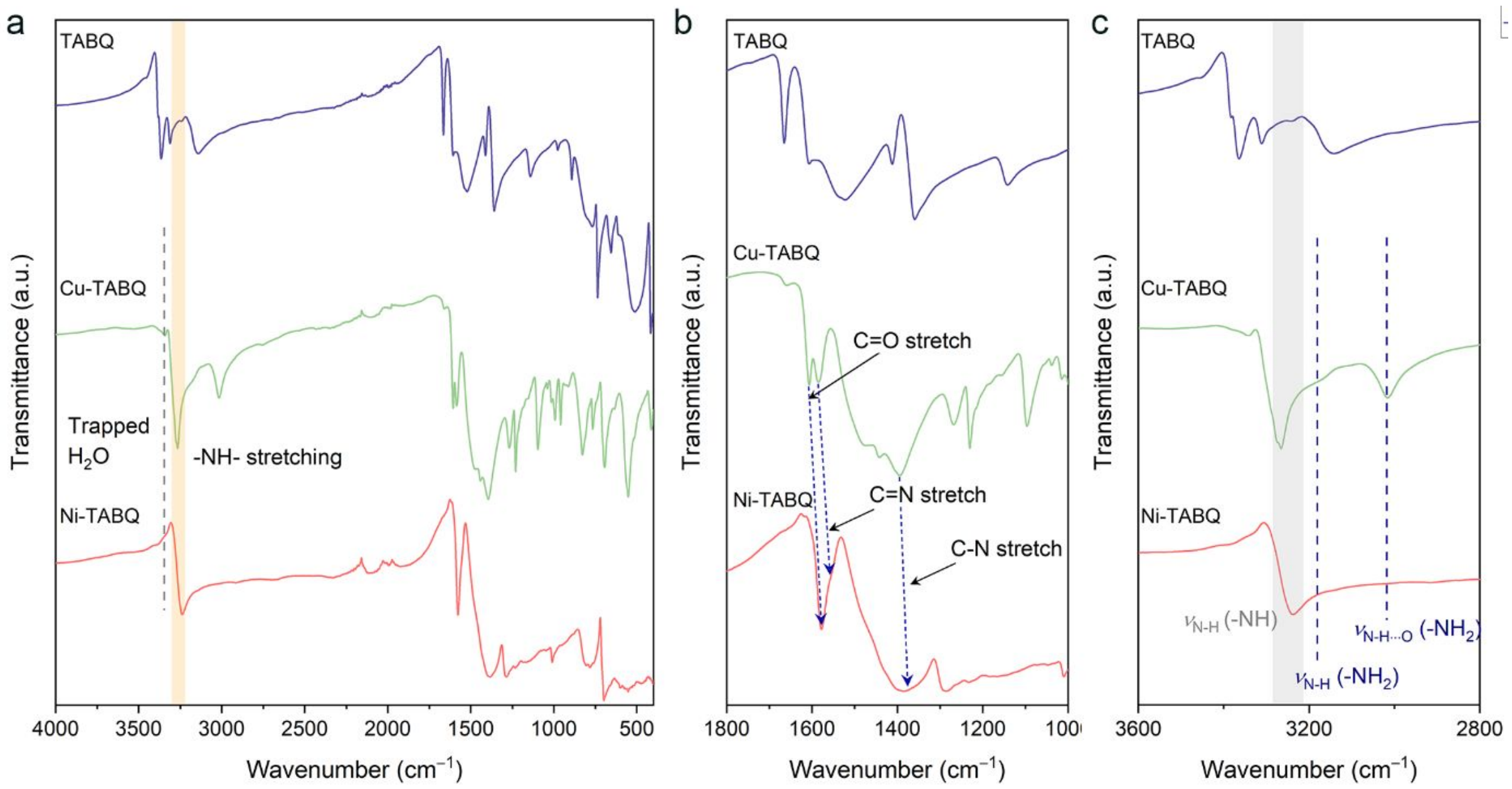

Figure S17. (**a**) ATR FT-IR spectra of TABQ (navy line), Cu-TABQ (light green), and Ni-TABQ (pink). (**b**) Magnified ATR FT-IR spectra between 1800 and 1000 cm^{-1}, confirming the presence of bis(iminobenzosemiquinoid) functionality. Ni-TABQ shows red-shifted C=O, C=N, and C-N stretching peaks compared to Cu-TABQ, indicating the higher degree of delocalization of the structure. (**c**) Magnified ATR FT-IR spectra in the N-H stretching region between 3600 and 2800 cm^{-1}, showing a single dominant N-H stretching feature in Ni-TABQ and multiple N-H stretching features in Cu-TABQ.

The TABQ ligand exhibited a characteristic C=O stretching vibration at 1665 cm^{-1}. Upon coordination with metal ions, this peak was red-shifted to 1578 cm^{-1} for Ni-TABQ and 1607 cm^{-1} for Cu-TABQ, indicating enhanced electronic delocalization within the polymer backbone, with Ni-TABQ displaying a significantly greater degree of delocalization (**Figure S17**).[1, 13] Furthermore, the C=N and C–N stretching bands in Ni-TABQ appeared at lower wavenumbers compared to those in Cu-TABQ, further supporting that Ni-TABQ possesses a more delocalized electronic structure. In the N-H stretching region, Ni-TABQ showed a single N-H stretching feature, whereas Cu-TABQ exhibited a more complex profile with multiple N-H stretching features, including a broad hydrogen-bonded N-H

envelope. This difference is consistent with the presence of distinct -NH and $-NH_2$ environments in Cu-TABQ and supports the local protonation/hydrogen-bonding motif proposed from the structural model.

12. Thermal gravimetric analyses (TGA)

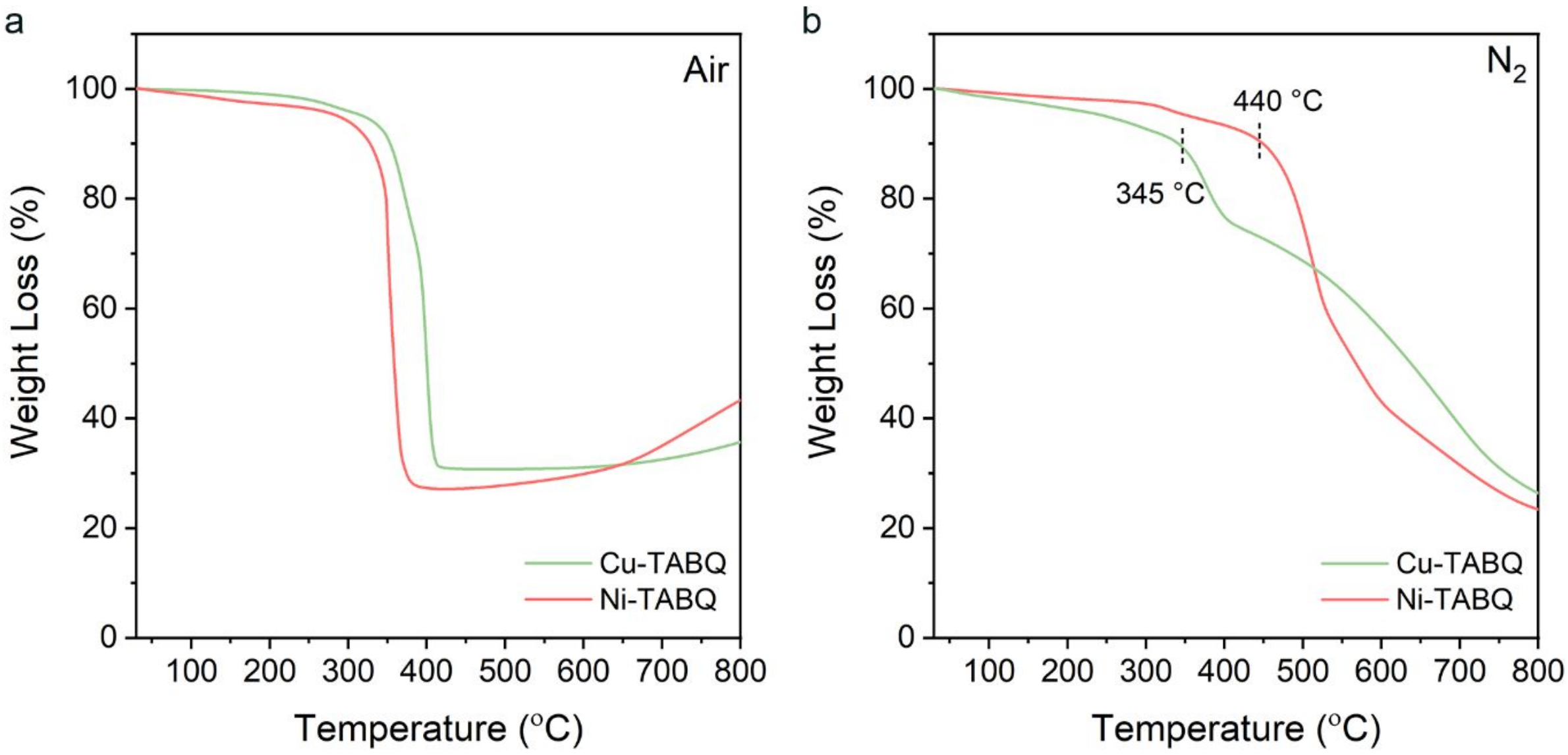

Figure S18. Thermal gravimetric analysis (TGA) thermogram of Cu-TABQ and Ni-TABQ under (**a**) air and (**b**) N_2 atmosphere.

The thermal stability of M-TABQ CCPs was evaluated by thermogravimetric analysis (TGA) under both air and N_2 atmosphere (**Figure S18**). Both Ni-TABQ and Cu-TABQ exhibited initial weight loss in the range of 310-325 °C under air. The subsequent mass increase observed after the decomposition of the CCP structures is attributed to the formation of copper oxide and nickel oxide under oxidative conditions.[14, 15] However, Ni-TABQ demonstrated significantly higher thermal stability, with major decomposition occurring at 440 °C, compared to 345 °C for Cu-TABQ. This enhanced thermal stability is attributed to the greater degree of electron delocalization in Ni-TABQ,[16] as evidenced by its distinct metal-ligand bonding environment.

13. Brunauer-Emmett-Teller (BET) analyses

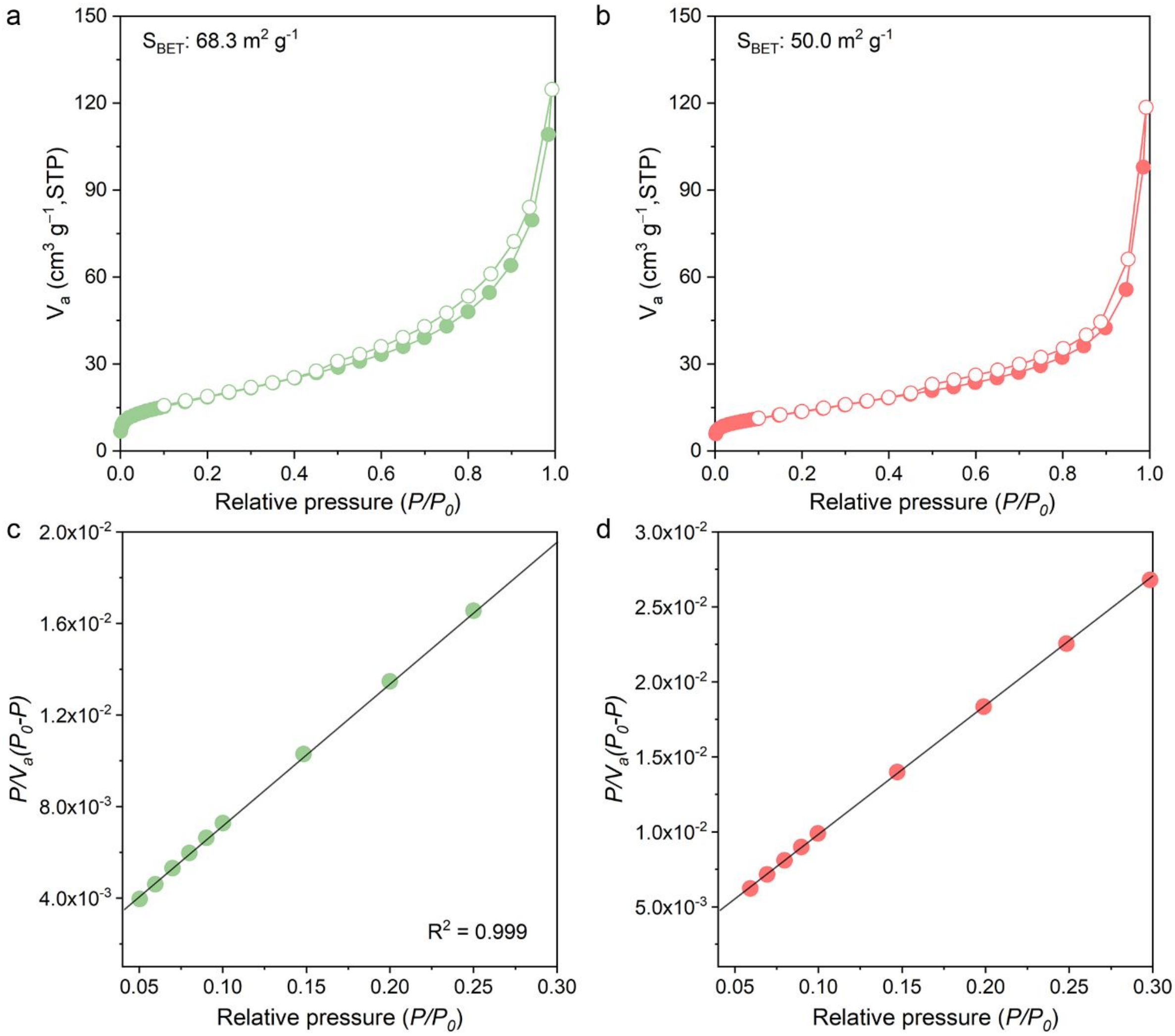

Figure S19. N_2 isotherms measured at 77 K of (**a**) Cu-TABQ, and (**b**) Ni-TABQ. (**c-d**) Corresponding BET plots.

The high crystallinity and dense packing of the 1D coordination polymer chains resulted in relatively low Brunauer-Emmett-Teller (BET) surface areas of 50.0 $m^2 g^{-1}$ and 68.3 $m^2 g^{-1}$ for Ni-TABQ and Cu-TABQ, respectively (**Figure S19**), which are comparable to those of previously reported other CCPs.[1, 8, 17] A small but non-negligible N_2 uptake observed in the low-pressure region is attributed to adsorption

at external surfaces, such as edges and defects, as well as possible interstitial voids arising from the packing and alignment of the linear CCP chains.[18]

14. X-ray photoelectron spectroscopy (XPS) study

XPS measurements confirmed the presence of bis(iminobenzosemiquinoid) units in M-TABQ, as evidenced by the deconvoluted N 1s spectra (**Figure S20**). Interestingly, high-resolution Cu 2p XPS spectrum exhibited a dominant Cu^{2+} peak at the binding energy of 934.9 eV and minor Cu^{+} peak at 932.8 eV, with a Cu^{2+}/Cu^{+} ratio of 3.5, despite the use of Cu^{+} precursor in the synthesis. This result suggests that partial oxidation of Cu^{+} to Cu^{2+} occurs during the solvothermal reaction, potentially slowing the coordination process and thereby facilitating the formation of larger crystallites compared to those obtained using Cu^{2+} salts. In contrast, the high-resolution Ni 2p XPS spectrum of Ni-TABQ confirmed the presence of exclusively divalent Ni^{2+} species (**Figure S21**).

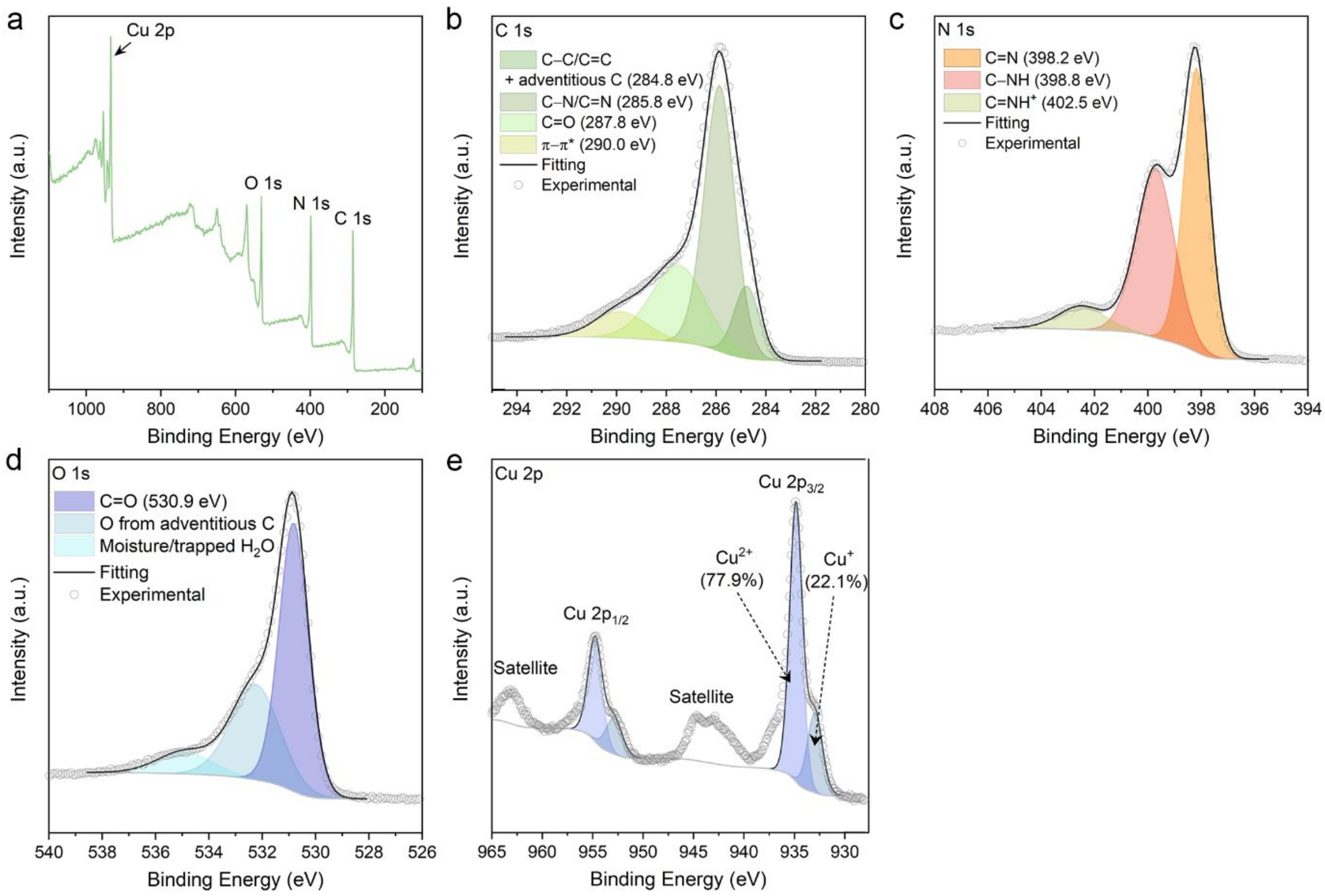

Figure S20. (**a**) XPS survey spectrum of Cu-TABQ. High-resolution spectra of (**b**) C 1s, (**c**) N 1s, (**d**) O 1s, and (**e**) Cu 2p.

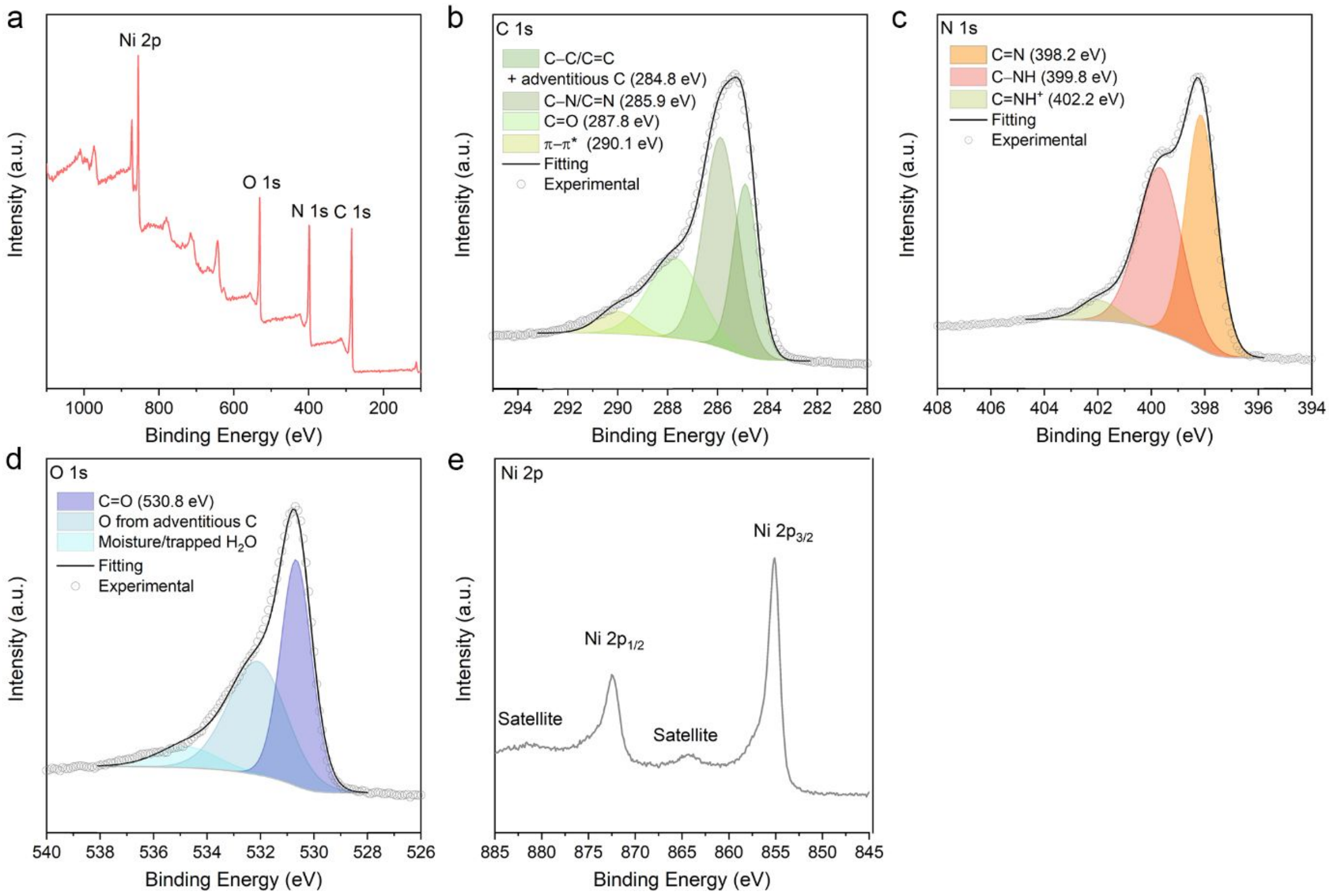

Figure S21. (**a**) XPS survey spectrum of Ni-TABQ. High-resolution spectra of (**b**) C 1s, (**c**) N 1s, (**d**) O 1s, and (**e**) Ni 2p.

15. Measurements of electrical conductivity

To measure the conductivity of prepared CCPs, the samples (25 - 30 mg) were pressed using a 6 × 6 mm square dry pellet pressing die. The materials were pressed for 10 min under a pressure of approximately 800 psi, producing pellets with thicknesses ranging from 390 to 450 μm. Pressed pellets of CCPs were cut into a cuboid by a blade. Four parallel gold (25 μm in diameter, Thermo Fisher Scientific) wire contacts were soldered to the pellet by carbon paste (Ted Pella, Inc.) diluted by diethyl succinate (**Figure S22**). The other ends of gold wires were attached to the sample puck. Conductivity measurements for each M-TABQ CCPs were repeated across three different synthetic batches to ensure reliability.

The temperature dependent electrical conductivity was measured in a liquid He cryostat of a Quantum Design PPMS Dynacool (Physical Property Measuring System) by using the four-contact probe method in direct current (DC) mode. The temperature was varied from 390 K to 310 K at 2.5 K min^{-1} for Cu-TABQ and 300 K to 100 K for Ni-TABQ, measuring the resistance in DC mode every 2.5 K.

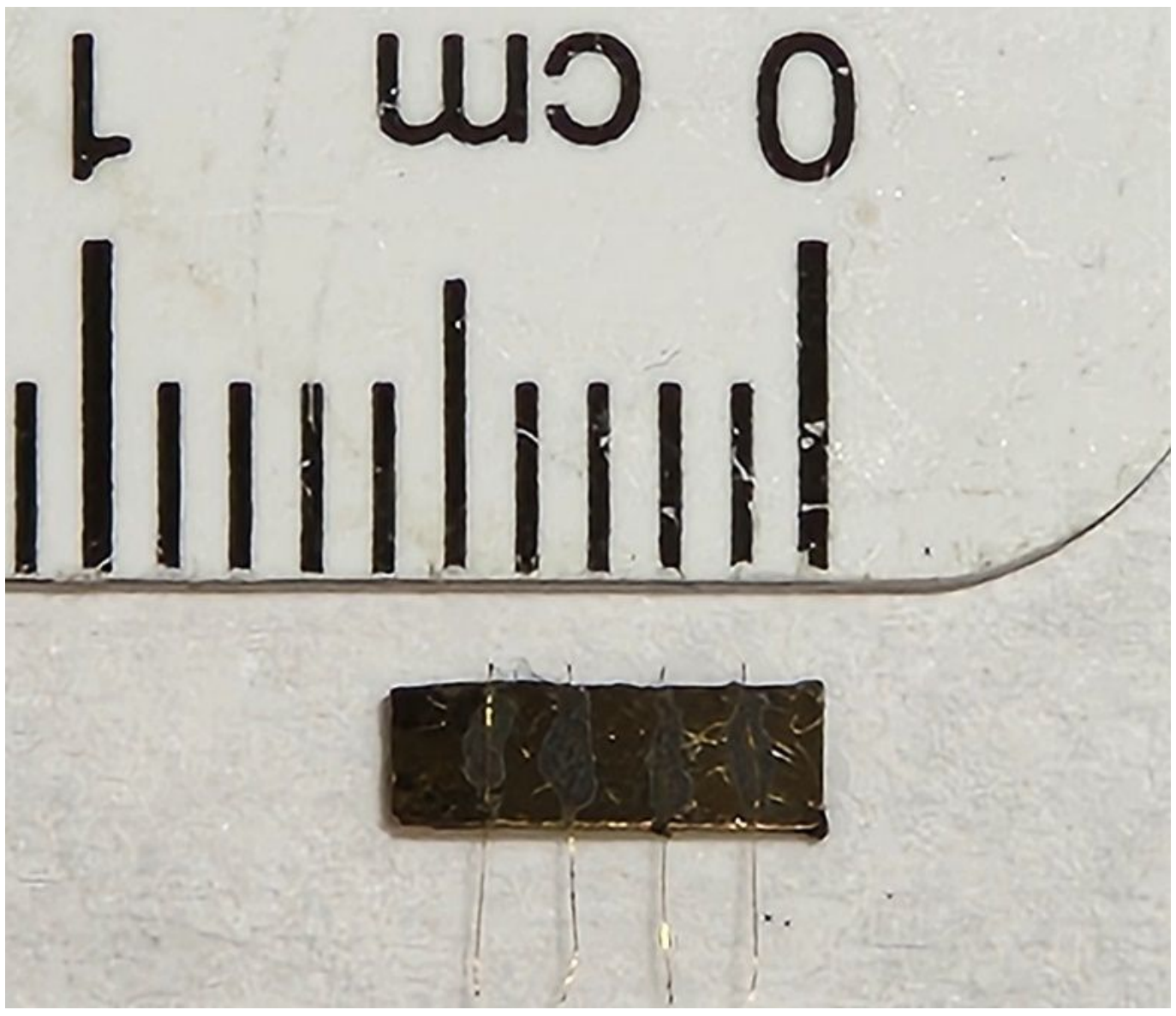

Figure S22. Image of four-contact probe device for conductivity measurement.

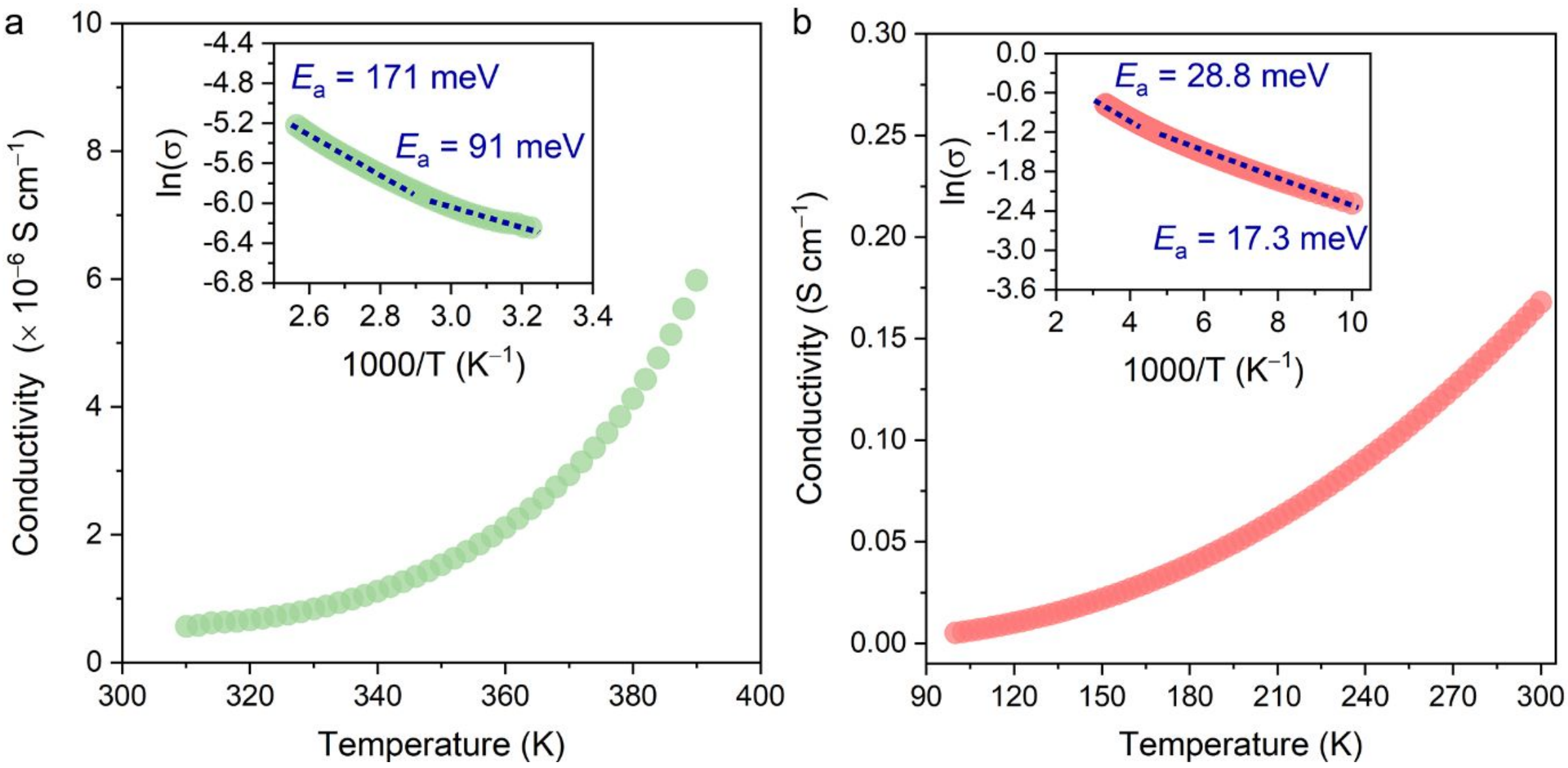

Figure S23. Electrical conductivity measurements of (**a**) Cu-TABQ and (**b**) Ni-TABQ as a function of temperature ranging from 310 to 390 K for Cu-TABQ and 100 to 300 K for Ni-TABQ, measured by four-contact probe conductivities. Insets: temperature dependence of the electrical conductivities of M-TABQ CCPs plotted according to the Arrhenius equation.

M-TABQ CCPs exhibited the Arrhenius-type dependence of the bulk electrical conductivities as a function of temperature, by fitting the Arrhenius equation, $\sigma = \sigma_0 \exp(-E_a/(k_B T))$, where σ_0 is a prefactor, k_B is the Boltzmann constant, and T is the absolute temperature. Ni-TABQ shows a slight increase in activation energy (E_a), from 28.8 meV above ~240 K to 17.3 meV below ~220 K. A similar change in E_a was previously reported for a related Ni-TABQ/Ni-1D system, where variable-temperature PXRD/Rietveld analysis revealed reversible structural variations, including changes in the interchain π-π stacking distance and intrachain dihedral angle.[1] Therefore, the change in E_a for Ni-TABQ is likely associated with temperature-dependent variations in interchain/intrachain electronic coupling.

In situ IR measurements of Cu-TABQ were performed using a Nicolet 6700 FT-IR spectrometer equipped with an environmental chamber. The Cu-TABQ sample was homogeneously mixed with dry KBr and loaded into the sample cup. Spectra were collected during heating and subsequent cooling under continuous N_2 flow.

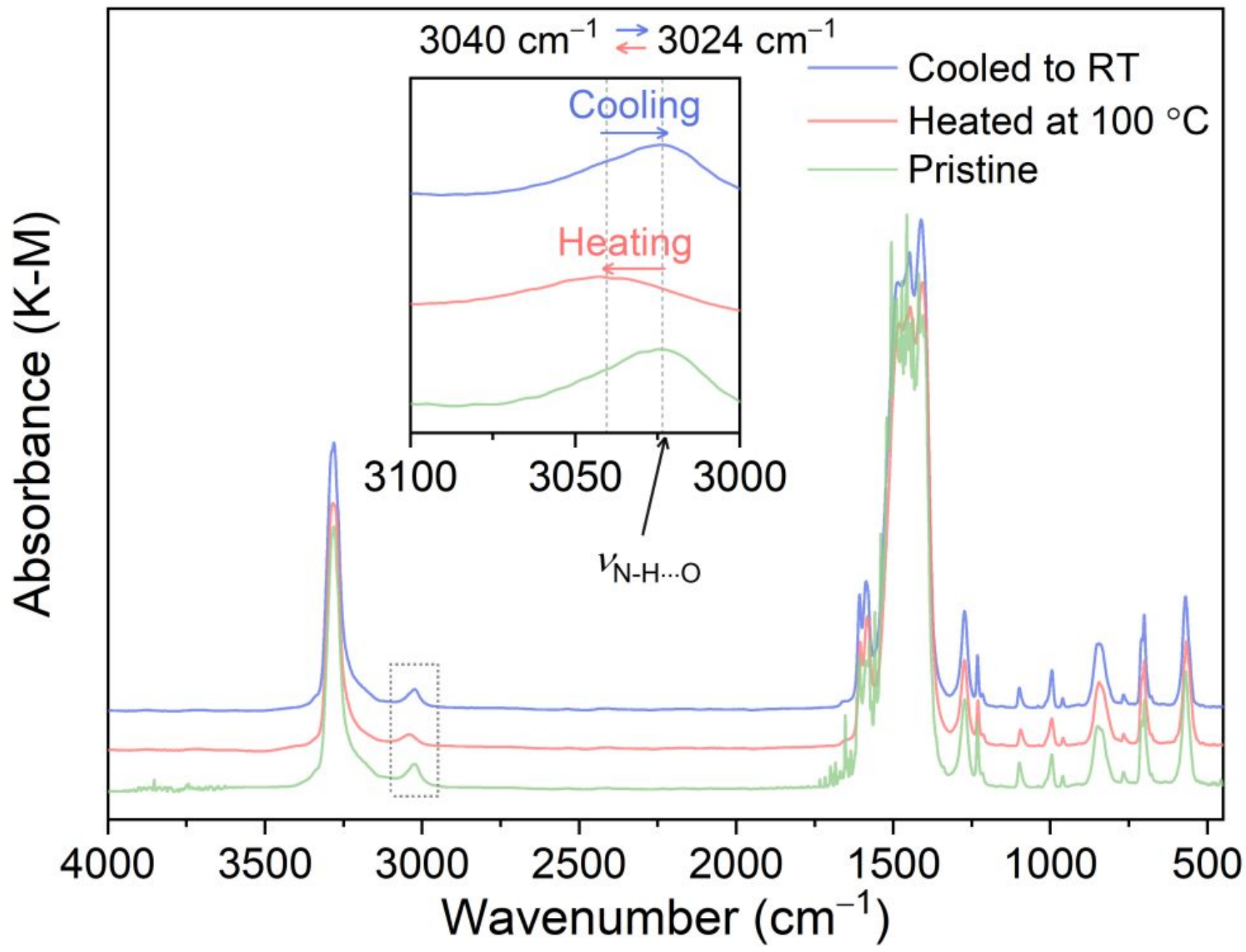

Figure S24. In situ IR spectra of Cu-TABQ under heating and cooling. The hydrogen-bonded N-H···O stretching band shifts from 3024 to 3040 cm^{-1} upon heating to 100 °C and returns toward its original position after cooling to room temperature, indicating a reversible change in the local hydrogen-bonding environment.

Cu-TABQ also exhibited two distinct E_a values of 171 meV above ~345 K and 91 meV below ~345 K. Given the local N-H···O hydrogen-bonding motif observed in the MicroED structure of Cu-TABQ, this change in activation energy may be related to thermally responsive hydrogen bonding. To probe this possibility, in situ IR measurements were performed under heating and cooling conditions. The band at 3024 cm^{-1},[19] assigned to a strongly hydrogen-bonded/protonated N-H stretching mode, shifts to 3040 cm^{-1} upon heating to 100 °C and returns toward its original position after cooling to room temperature. This reversible blue shift suggests weakening of the local N-H···O hydrogen-bonding interaction at

elevated temperature and recovery upon cooling. The shift to higher wavenumber upon heating is therefore consistent with a reduction in N-H···O hydrogen-bond strength, which decreases the perturbation of the N-H bond by the neighboring carbonyl oxygen.[20]

These results suggest that the change in E_a for Cu-TABQ is more appropriately described as being associated with reversible changes in the local hydrogen-bonding environment, rather than as direct evidence for an abrupt structural phase transition or well-defined interchain displacement. Such thermally responsive hydrogen bonding may affect the local framework arrangement and charge-transport pathway, contributing to the observed temperature-dependent activation energies.

16. Hall effect measurement

A DC field Hall effect measurement was performed on pressed pellets of M-TABQ CCPs using a Design PPMS Dynacool equipped with a 9 T superconducting magnet. The magnetic field was swept from −3 to +3 T during the measurements. To prepare samples, approximately 25 - 30 mg of each CCP powder was pressed into pellets using a 6 × 6 mm square dry pellet pressing die. The materials were pressed for 10 min under a pressure of approximately 800 psi, yielding pellets with thicknesses ranging from 390 to 450 µm. The pressed pellets were then cut into square shapes using a blade. Four gold wire (25 µm in diameter, Thermo Fisher Scientific) contacts were soldered to the four corners of pellet within carbon paste (Ted Pella, Inc.) diluted by diethyl succinate (**Figure S25**). The other end of gold wires were pasted onto the sample puck.

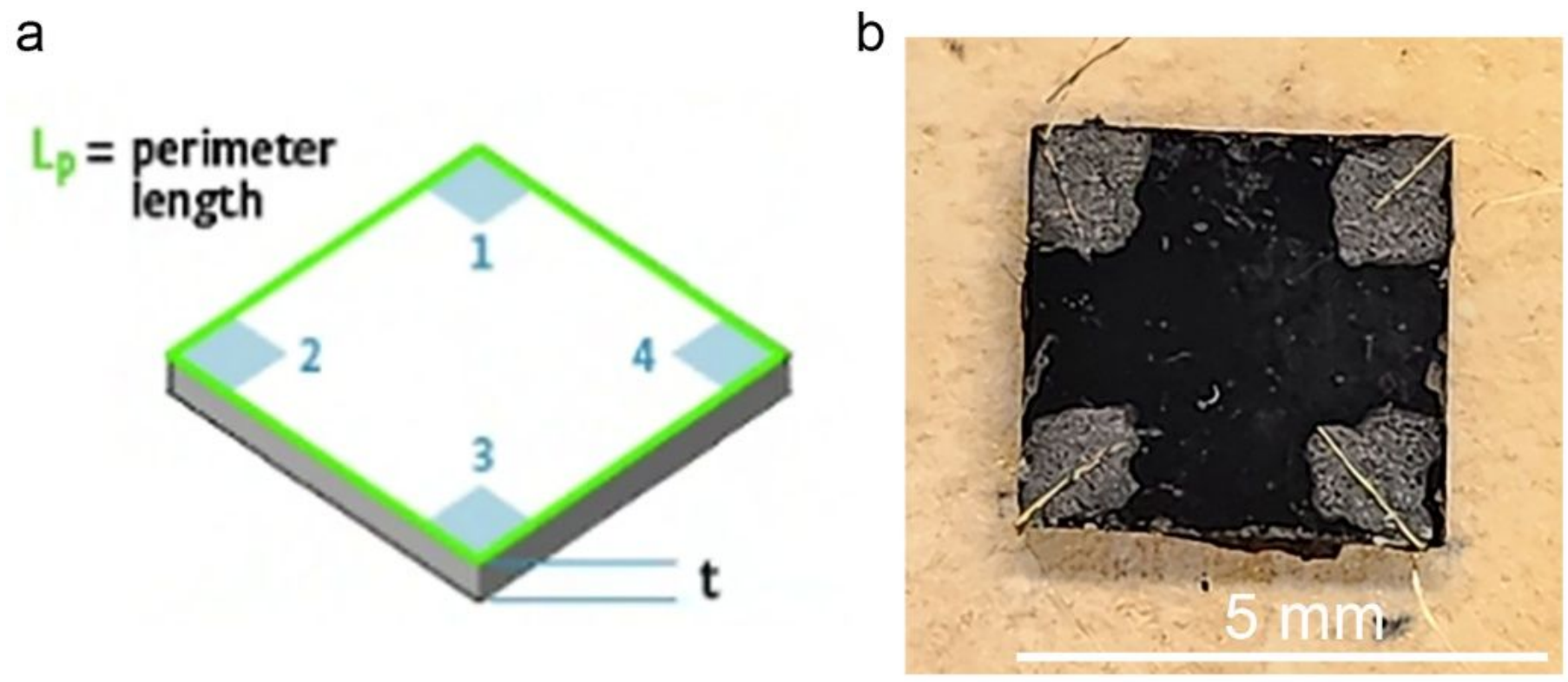

Figure S25. Image of four-contact probe device for conductivity measurement.

In Hall effect measurements, the Hall coefficient (R_H) is determined by **equation S1**

$$R_H = \frac{R_{xy}\, t}{B} \qquad \textbf{(S1)}$$

Here, R_{xy} is the Hall resistance, B is the applied magnetic field, and t is the sample thickness. The Hall resistance was obtained by antisymmetrizing the raw resistance data (**equation S2**) according to:

$$R_{xy}(B) = \frac{R(B) - R(-B)}{2} \qquad \textbf{(S2)}$$

in order to remove contributions from longitudinal resistance arising from contact misalignment and sample inhomogeneity. This antisymmetrization procedure is widely used to extract the intrinsic Hall response in polycrystalline samples.[21, 22] Multiple independently prepared pellet devices exhibited consistent linear Hall responses after antisymmetrization, supporting the reproducibility of the extracted Hall behavior despite minor variations arising from sample inhomogeneity.

According to the semiconducting carrier type, $R_H < 0$ indicates *n*-type carriers. The carrier concentration and carrier mobility are determined by **equation S3** and **equation S4**, respectively.

$$n = \frac{1}{eR_H} \qquad \textbf{(S3)}$$

Here, $e = 1.602 \times 10^{-19}$ C is the elementary charge.

$$\mu = R_H \times \sigma \qquad \textbf{(S4)}$$

σ represents the electrical conductivity of the material.

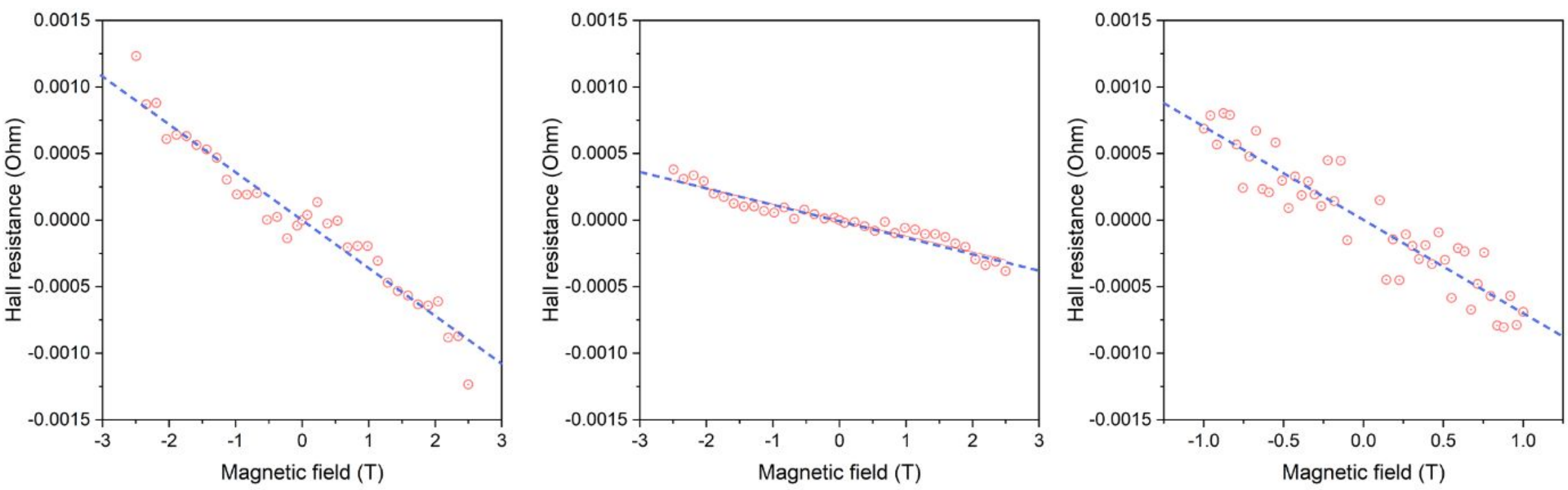

Figure S26. Magnetic field dependence of the Hall resistance for three independently prepared Ni-TABQ pellet devices measured at 300 K after antisymmetrization of the Hall data. The dashed lines represent linear fits to the extracted Hall resistance, demonstrating reproducible Hall responses with consistent linear magnetic-field dependence across independently measured samples.

17. Density functional theory (DFT)

All calculations were performed using density functional theory as implemented in the Vienna Ab Initio Simulations Package (VASP 6.5.1)[23] using a plane wave basis set.[24] The periodic frameworks were geometrically optimized using a cutoff energy of 520 eV using the PBEsol functional. The structural optimization with fixed lattice parameters for Ni-TABQ was performed using the r^2SCAN meta-GGA functional. The ionic and electronic convergence criteria were 5 meV/Å and 10^{-6} eV, respectively. Transition metals with partially-occupied *d* orbitals were treated with the DFT + U formalism to model antiferromagnetic coupling in the scheme introduced by Dudarev et al.,[25] The effective Hubbard U values (Ni: 6.4 eV, Cu: 4.0 eV) were taken from Wang et al. as fit to experimental oxidation values for transition metal oxides.[26] Dispersion corrections were included to model the van der Waals interactions between the sheets of the material using Grimme's D3 scheme.[27] After structural relaxation, the electronic band structures were computed using the HSEsol functional with an electronic convergence criteria of 10^{-6} eV. The carrier density in the first conduction band of near-metallic Ni-TABQ was computed using BoltzTraP2,[28] with the chemical potential determined by charge neutrality under Fermi-Dirac occupations at 300 K. Effective masses for the semiconducting Cu-TABQ band structure were obtained by parabolic fitting of the band edges using sumo.[29]

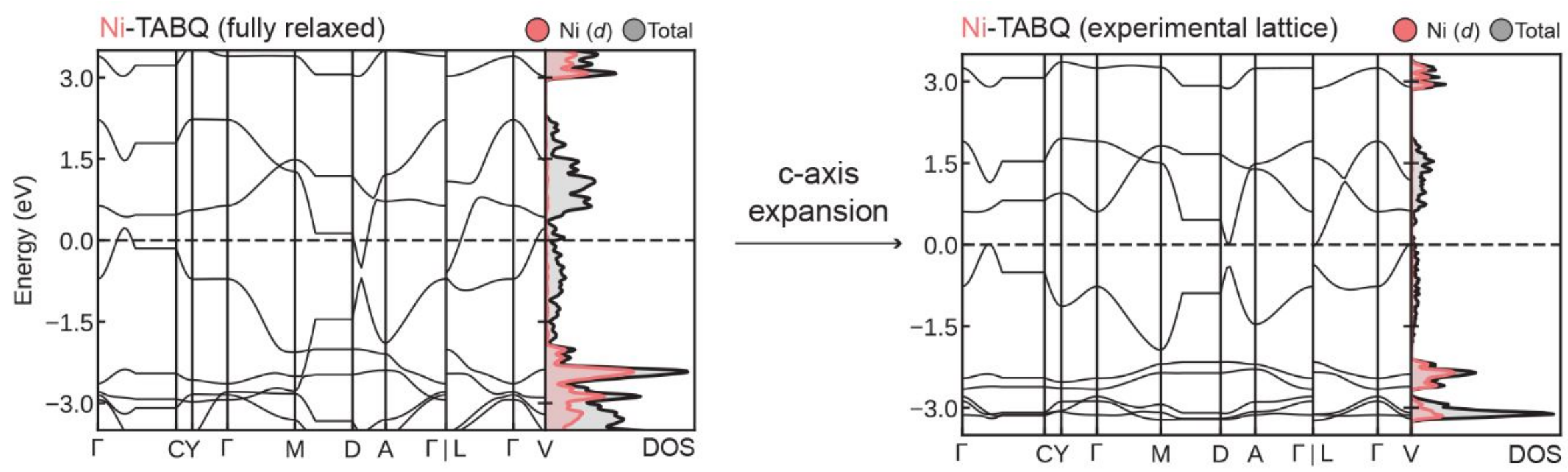

Figure S27. Interlayer compression in the fully-relaxed Ni-TABQ unit cell results in a metallic band structure. Fixing the lattice parameters to the Rietveld-refined values recovers a near-metallic band structure.

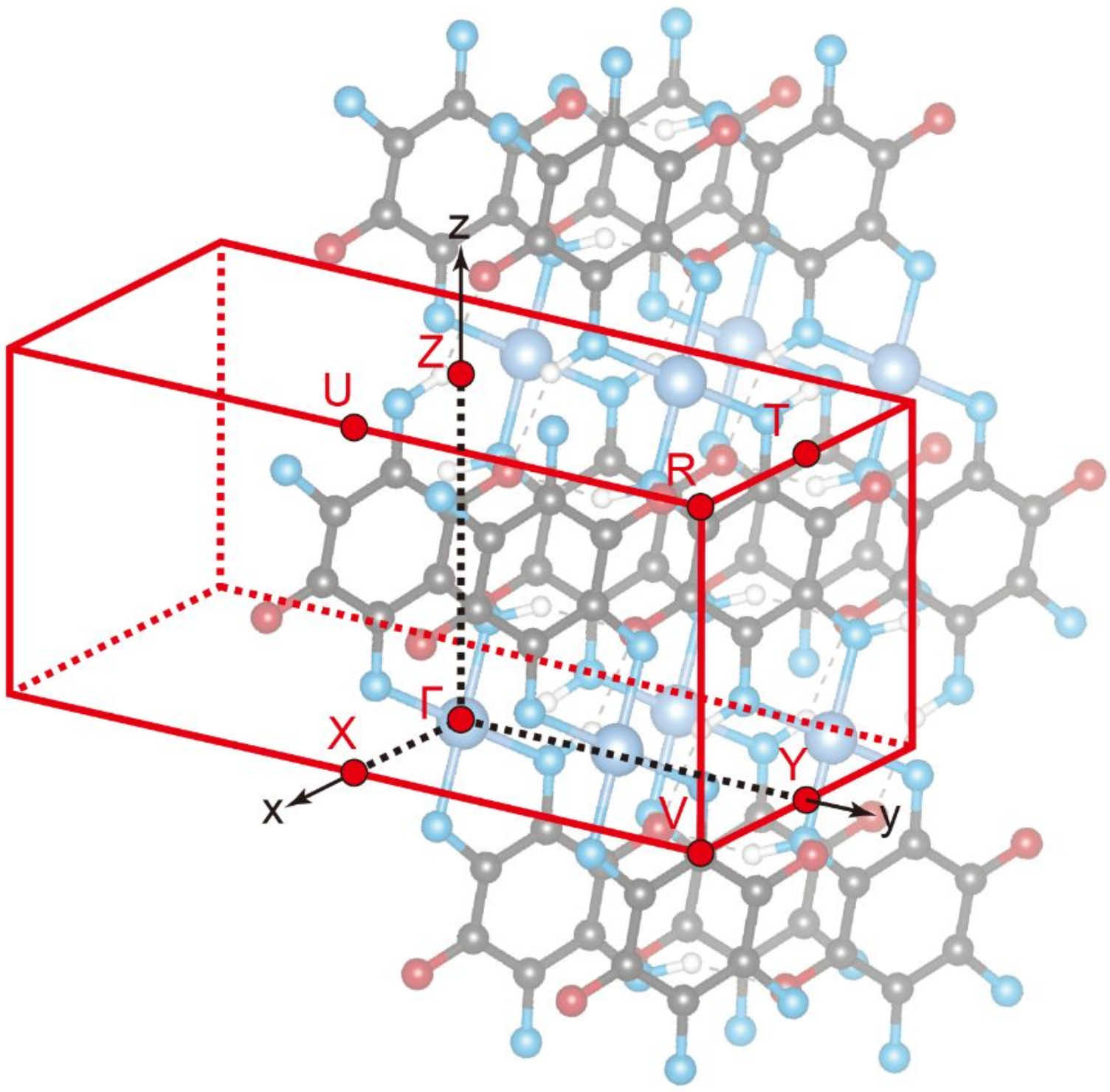

Figure S28. Brillouin zone of the Cu-TABQ structure.

The vibrational spectra of Cu-TABQ and Ni-TABQ were calculated using the finite differences method, a plane wave cutoff energy of 520 eV and an electronic convergence threshold of 10^{-8} eV. Vibrational frequencies and IR intensities were extracted from the diagonalized dynamical matrix and the Born effective charge tensors were computed via density functional perturbation theory. IR intensities were obtained from the square of the vibrational mode dipole derivative. Spectra were generated by Lorentzian broadening of the resulting intensities, and individual normal modes were visualized by displacing atoms along their eigenvectors to assign each band.

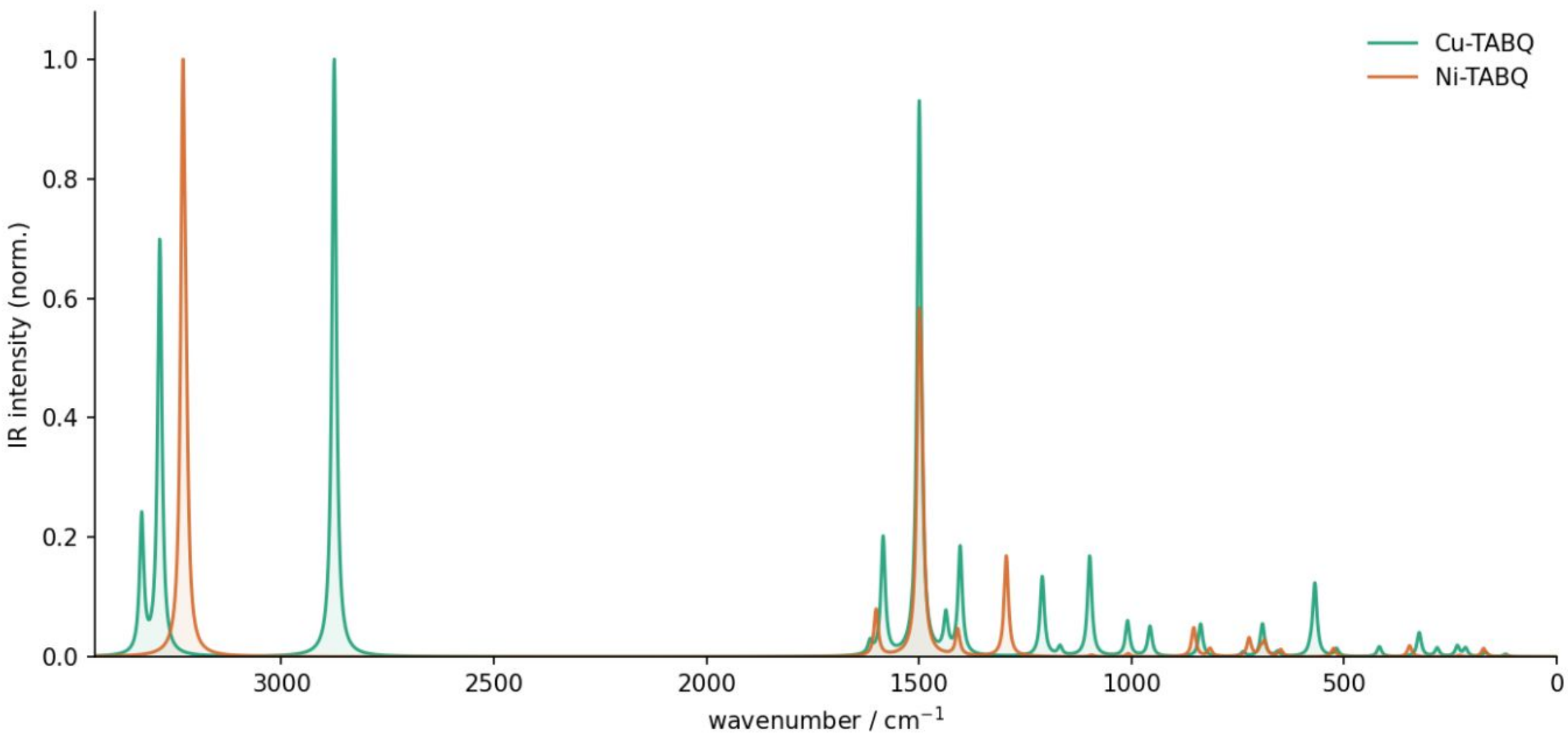

Figure S29. Computed vibrational spectra of Cu-TABQ and Ni-TABQ.

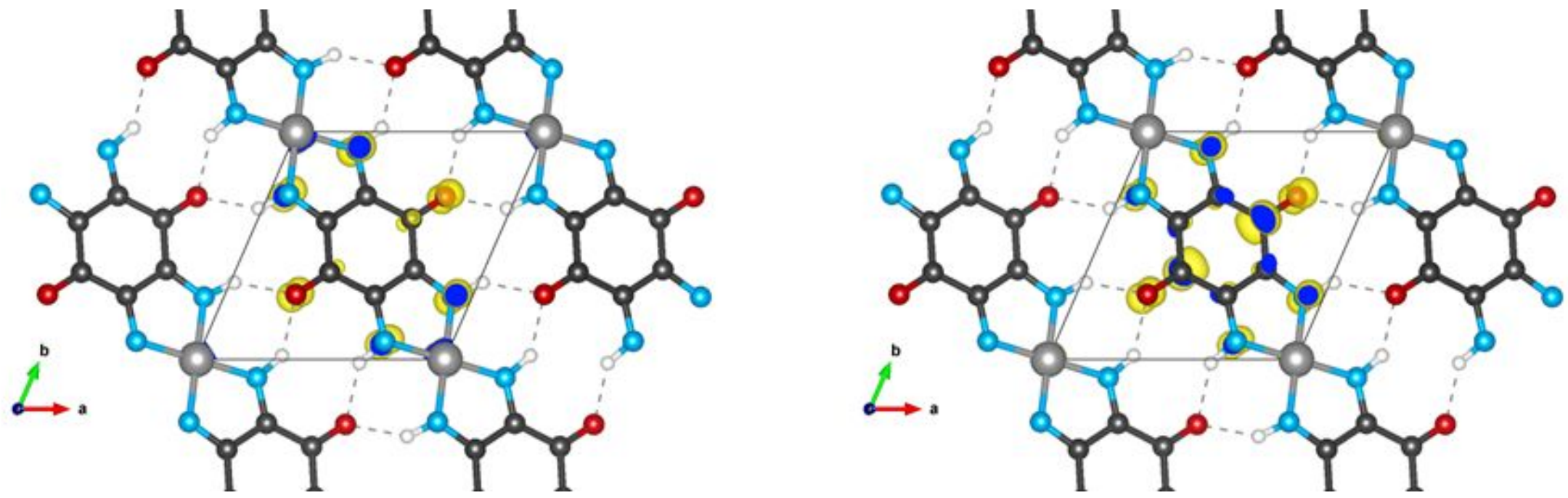

Figure S30. The average charge density for the valence band edge (left) and conduction band edge (right) across all k-points for Ni-TABQ, showing p_z character in the valence band and π^* character in the conduction band.

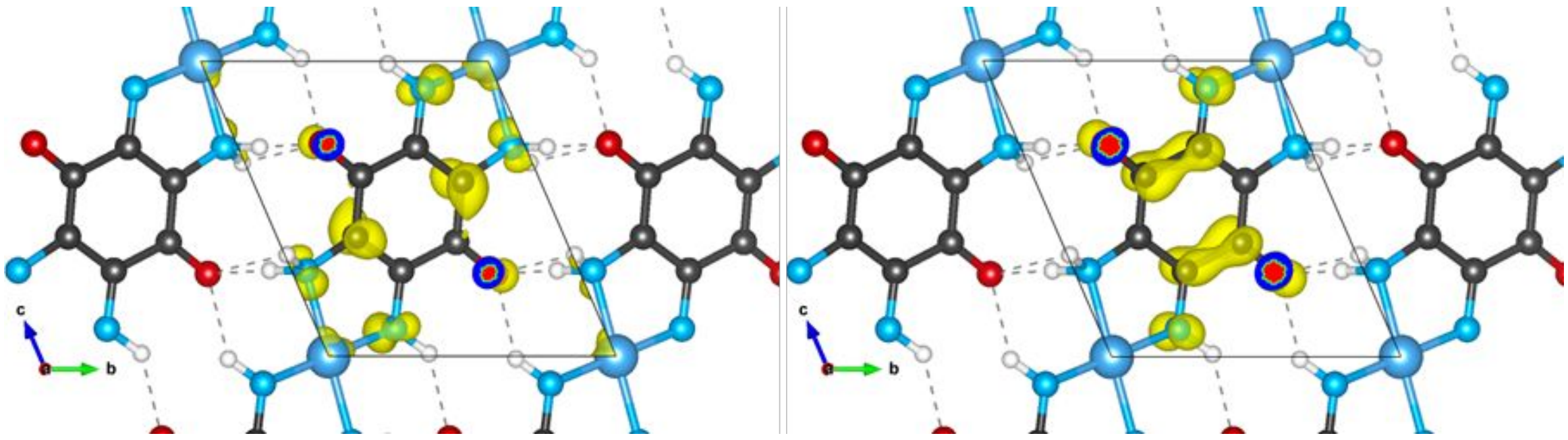

Figure S31. The average charge density for the valence band edge (left) and conduction band edge (right) across all k-points for Cu-TABQ, showing p_z character in the valence band and π^* character in the conduction band.

To investigate the charge transport mechanism in these materials, a single-point hybrid band structure calculation was performed using the Cu-TABQ structure but with Cu replaced by Ni. The band structure obtained was qualitatively identical to the parent Cu-TABQ band structure, indicating that the metal is secondary to the ligand in driving the electronic properties of the framework.

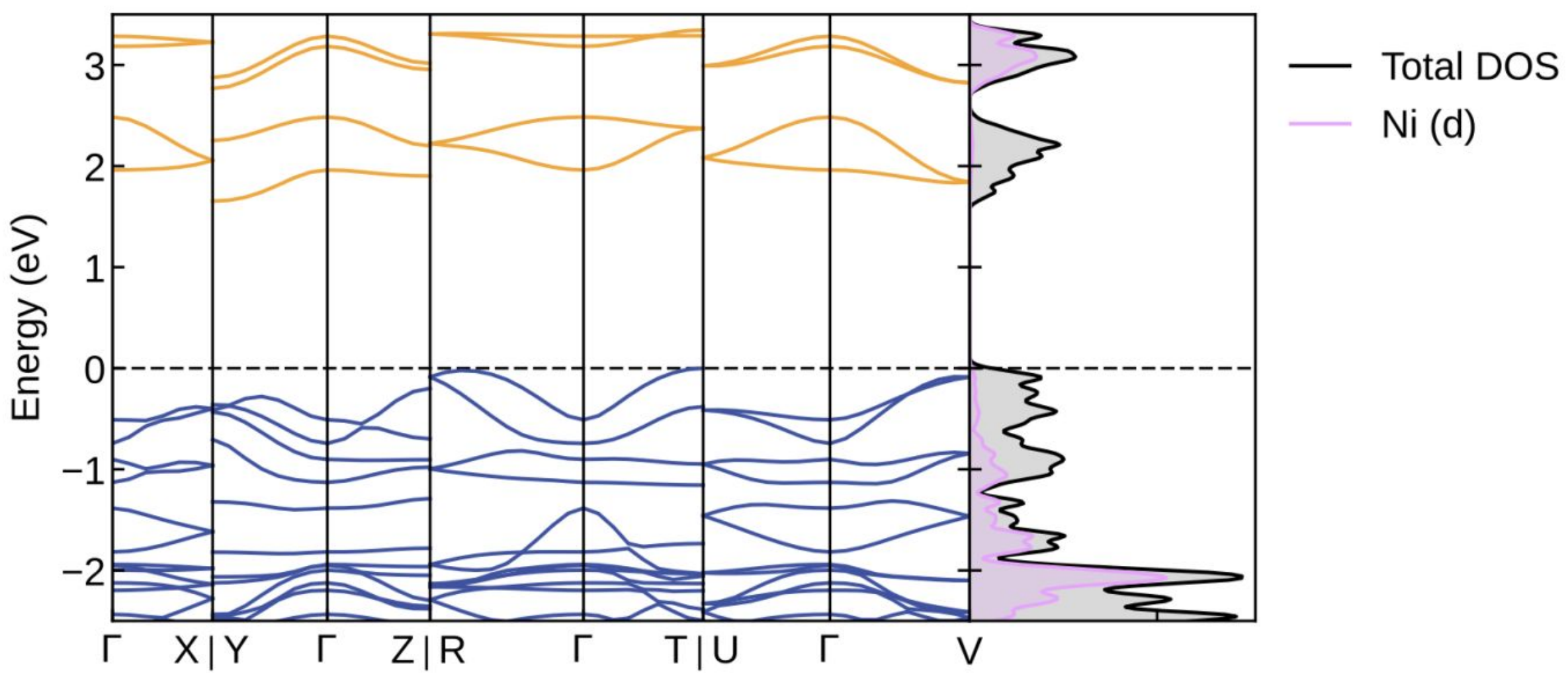

Figure S32. Single-point hybrid-DFT band structure and projected density of states for the distorted Cu-TABQ structure with Cu replaced by Ni, while retaining the protonated Cu-TABQ geometry without further structural relaxation. The resulting band structure remains qualitatively similar to that of the parent Cu-TABQ model.

The DFT-relaxed structures used in this work are provided below in CIF format to facilitate reproducibility. These structures are computational models and should not be interpreted as experimentally refined crystallographic structures. The lattice parameters and atomic coordinates correspond to the final DFT-relaxed geometries.

DFT-relaxed computed structure of Ni-TABQ.cif

```
#======================================================================
# CRYSTAL DATA
#----------------------------------------------------------------------
data_Ni-TABQ_DFT_relaxed

_chemical_name_common                  'Ni-TABQ computed structure'
_cell_length_a                         7.169050
_cell_length_b                         7.169050
_cell_length_c                         3.767100
_cell_angle_alpha                      83.808807
_cell_angle_beta                       96.191193
_cell_angle_gamma                      115.580482
_cell_volume                           173.208891
_space_group_name_H-M_alt              'P 1'
_space_group_IT_number                 1

loop_
_space_group_symop_operation_xyz
   'x, y, z'

loop_
   _atom_site_label
```

```
_atom_site_occupancy
_atom_site_fract_x
_atom_site_fract_y
_atom_site_fract_z
_atom_site_adp_type
_atom_site_U_iso_or_equiv
_atom_site_type_symbol
C1    1.0    0.391150    0.287057    0.428097    Uiso    ? C
C2    1.0    0.608850    0.712943    0.571903    Uiso    ? C
C3    1.0    0.287057    0.391150    0.571903    Uiso    ? C
C4    1.0    0.712943    0.608850    0.428097    Uiso    ? C
C5    1.0    0.608544    0.391456    0.345162    Uiso    ? C
C6    1.0    0.391456    0.608544    0.654838    Uiso    ? C
N1    1.0    0.269152    0.090626    0.379127    Uiso    ? N
N2    1.0    0.730848    0.909374    0.620873    Uiso    ? N
N3    1.0    0.090626    0.269152    0.620873    Uiso    ? N
N4    1.0    0.909374    0.730848    0.379127    Uiso    ? N
H1    1.0    0.334035    0.008039    0.277851    Uiso    ? H
H2    1.0    0.665965    0.991961    0.722149    Uiso    ? H
H3    1.0    0.008039    0.334035    0.722149    Uiso    ? H
H4    1.0    0.991961    0.665965    0.277851    Uiso    ? H
O1    1.0    0.697470    0.302530    0.211904    Uiso    ? O
O2    1.0    0.302530    0.697470    0.788096    Uiso    ? O
Ni1   1.0    0.000000    0.000000    0.500000    Uiso    ? Ni
```

Cu-TABQ computed structure.cif

```
#======================================================================
# CRYSTAL DATA
#----------------------------------------------------------------------
data_ Cu-TABQ_DFT_relaxed

_chemical_name_common                  'Cu-TABQ computed structure'
_cell_length_a                         6.994560
_cell_length_b                         6.494745
_cell_length_c                         7.338901
_cell_angle_alpha                      112.169662
_cell_angle_beta                       99.851562
_cell_angle_gamma                      94.667030
_cell_volume                           300.274925
_space_group_name_H-M_alt              'P 1'
_space_group_IT_number                 1

loop_
_space_group_symop_operation_xyz
   'x, y, z'

loop_
   _atom_site_label
   _atom_site_occupancy
   _atom_site_fract_x
   _atom_site_fract_y
   _atom_site_fract_z
   _atom_site_adp_type
   _atom_site_U_iso_or_equiv
```

_atom_site_type_symbol

Cu1	1.0	0.249996	0.999997	0.999997	Uiso	? Cu
Cu2	1.0	0.749996	0.999994	0.999994	Uiso	? Cu
O1	1.0	0.046236	0.704024	0.279230	Uiso	? O
O2	1.0	0.546235	0.704021	0.279227	Uiso	? O
O3	1.0	0.453760	0.295966	0.720764	Uiso	? O
O4	1.0	0.953759	0.295963	0.720761	Uiso	? O
N1	1.0	0.192550	0.950567	0.709221	Uiso	? N
N2	1.0	0.692549	0.950565	0.709217	Uiso	? N
N3	1.0	0.307441	0.049413	0.290772	Uiso	? N
N4	1.0	0.807439	0.049409	0.290768	Uiso	? N
N5	1.0	0.136390	0.270111	0.093339	Uiso	? N
N6	1.0	0.636391	0.270110	0.093336	Uiso	? N
N7	1.0	0.363598	0.729878	0.906652	Uiso	? N
N8	1.0	0.863600	0.729876	0.906649	Uiso	? N
C1	1.0	0.182946	0.381613	0.290001	Uiso	? C
C2	1.0	0.682947	0.381611	0.289997	Uiso	? C
C3	1.0	0.317042	0.618380	0.709991	Uiso	? C
C4	1.0	0.817043	0.618379	0.709987	Uiso	? C
C5	1.0	0.219066	0.726047	0.594305	Uiso	? C
C6	1.0	0.719066	0.726045	0.594301	Uiso	? C
C7	1.0	0.280927	0.273942	0.405688	Uiso	? C
C8	1.0	0.780927	0.273940	0.405685	Uiso	? C
C9	1.0	0.143937	0.616316	0.387421	Uiso	? C
C10	1.0	0.643937	0.616313	0.387417	Uiso	? C
C11	1.0	0.356055	0.383683	0.612577	Uiso	? C
C12	1.0	0.856055	0.383680	0.612574	Uiso	? C
H1	1.0	0.061045	0.991336	0.655937	Uiso	? H

H2	1.0	0.561042	0.991329	0.655934	Uiso	? H
H3	1.0	0.438951	0.008664	0.344055	Uiso	? H
H4	1.0	0.938948	0.008657	0.344051	Uiso	? H
H5	1.0	0.072374	0.353034	0.009156	Uiso	? H
H6	1.0	0.572376	0.353032	0.009154	Uiso	? H
H7	1.0	0.427615	0.646956	0.990838	Uiso	? H
H8	1.0	0.927616	0.646954	0.990834	Uiso	? H
H9	1.0	0.700648	0.924793	0.290829	Uiso	? H
H10	1.0	0.200652	0.924795	0.290834	Uiso	? H
H11	1.0	0.299352	0.075200	0.709161	Uiso	? H
H12	1.0	0.799347	0.075198	0.709157	Uiso	? H

References

1. Chen, T.; Dou, J.-H.; Yang, L.; Sun, C.; Oppenheim, J. J.; Li, J.; Dincă, M., Dimensionality Modulates Electrical Conductivity in Compositionally Constant One-, Two-, and Three-Dimensional Frameworks. *J. Am. Chem. Soc.* **2022,** *144* (12), 5583-5593.

2. Ito, S.; White, F. J.; Okunishi, E.; Aoyama, Y.; Yamano, A.; Sato, H.; Ferrara, J. D.; Jasnowski, M.; Meyer, M., Structure determination of small molecule compounds by an electron diffractometer for 3D ED/MicroED. *CrystEngComm* **2021,** *23* (48), 8622-8630.

3. Truong, K.-N.; Ito, S.; Wojciechowski, J. M.; Göb, C. R.; Schürmann, C. J.; Yamano, A.; Del Campo, M.; Okunishi, E.; Aoyama, Y.; Mihira, T.; Hosogi, N.; Benet-Buchholz, J.; Escudero-Adán, E. C.; White, F. J.; Ferrara, J. D.; Bücker, R. Making the Most of 3D Electron Diffraction: Best Practices to Handle a New Tool *Symmetry* [Online], 2023.

4. Rigaku Oxford Diffraction, (2024), CrysAlisPro Software system, version 1.171.44.97, Rigaku Corporation, Wroclaw, Poland.

5. Sheldrick, G., SHELXT - Integrated space-group and crystal-structure determination. *Acta Crystallographica Section A* **2015,** *71* (1), 3-8.

6. Sheldrick, G., Crystal structure refinement with SHELXL. *Acta Crystallographica Section C* **2015,** *71* (1), 3-8.

7. Dolomanov, O. V.; Bourhis, L. J.; Gildea, R. J.; Howard, J. A. K.; Puschmann, H., OLEX2: a complete structure solution, refinement and analysis program. *J. Appl. Crystallogr.* **2009,** *42* (2), 339-341.

8. Fan, K.; Li, J.; Xu, Y.; Fu, C.; Chen, Y.; Zhang, C.; Zhang, G.; Ma, J.; Zhai, T.; Wang, C., Single Crystals of a Highly Conductive Three-Dimensional Conjugated Coordination Polymer. *J. Am. Chem. Soc.* **2023,** *145* (23), 12682-12690.

9. Sato, M.; Takeda, T.; Hoshino, N.; Akutagawa, T., Electronic and crystal structures of 1,2,3-triazole-fused p-benzoquinone derivatives. *CrystEngComm* **2017,** *19* (6), 910-917.

10. Meng, Z.; Jones, C. G.; Farid, S.; Khan, I. U.; Nelson, H. M.; Mirica, K. A., Unraveling the Electrical and Magnetic Properties of Layered Conductive Metal-Organic Framework With Atomic Precision. *Angew. Chem. Int. Ed.* **2022,** *61* (6), e202113569.

11. Sun, L.; Hendon, C. H.; Minier, M. A.; Walsh, A.; Dincă, M., Million-Fold Electrical Conductivity Enhancement in Fe2(DEBDC) versus Mn2(DEBDC) (E = S, O). *J. Am. Chem. Soc.* **2015,** *137* (19), 6164-6167.

12. Balch, A. L.; Holm, R. H., Complete Electron-Transfer Series of the [M-N4] Type. *J. Am. Chem. Soc.* **1966,** *88* (22), 5201-5209.

13. Calderazzo, F.; Marchetti, F.; Pampaloni, G.; Passarelli, V., Co-ordination properties of

1,10-phenanthroline-5,6-dione towards group 4 and 5 metals in low and high oxidation states†. *J. Chem. Soc., Dalton Trans.* **1999,** (24), 4389-4396.

14. Bridges, D. W.; Baur, J. P.; Baur, G. S.; Fassell, W. M., Oxidation of Copper to Cu2 O and CuO (600°–1000°C and 0.026–20.4 atm Oxygen). *J. Electrochem. Soc.* **1956,** *103* (9), 475.

15. Sainju, R.; Rathnayake, D.; Tan, H.; Bollas, G.; Dongare, A. M.; Suib, S. L.; Zhu, Y., In Situ Studies of Single-Nanoparticle-Level Nickel Thermal Oxidation: From Early Oxide Nucleation to Diffusion-Balanced Oxide Thickening. *ACS Nano* **2022,** *16* (4), 6468-6479.

16. Shaik, S. S.; Hiberty, P. C., When does electronic delocalization become a driving force of molecular shape and stability? 1. The aromatic sextet. *J. Am. Chem. Soc.* **1985,** *107* (11), 3089-3095.

17. Geng, J.; Ni, Y.; Zhu, Z.; Wu, Q.; Gao, S.; Hua, W.; Indris, S.; Chen, J.; Li, F., Reversible Metal and Ligand Redox Chemistry in Two-Dimensional Iron–Organic Framework for Sustainable Lithium-Ion Batteries. *J. Am. Chem. Soc.* **2023,** *145* (3), 1564-1571.

18. Shang, S.; Du, C.; Liu, Y.; Liu, M.; Wang, X.; Gao, W.; Zou, Y.; Dong, J.; Liu, Y.; Chen, J., A one-dimensional conductive metal-organic framework with extended π-d conjugated nanoribbon layers. *Nat. Commun.* **2022,** *13* (1), 7599.

19. Hansen, P. E.; Spanget-Larsen, J. NMR and IR Investigations of Strong Intramolecular Hydrogen Bonds *Molecules* [Online], 2017, p. 552.

20. Li, X.; Liu, L.; Schlegel, H. B., On the Physical Origin of Blue-Shifted Hydrogen Bonds. *J. Am. Chem. Soc.* **2002,** *124* (32), 9639-9647.

21. Biscaras, J.; Chen, Z.; Paradisi, A.; Shukla, A., Onset of two-dimensional superconductivity in space charge doped few-layer molybdenum disulfide. *Nat. Commun.* **2015,** *6* (1), 8826.

22. van der Pauw, L. J., A Method of Measuring Specific Resistivity and Hall Effect of Discs of Arbitrary Shape. *Philips Research Reports* **1958,** *13*, 1-9.

23. Kresse, G.; Furthmüller, J., Efficient iterative schemes for ab initio total-energy calculations using a plane-wave basis set. *Physical Review B* **1996,** *54* (16), 11169-11186.

24. Kresse, G.; Furthmüller, J., Efficiency of ab-initio total energy calculations for metals and semiconductors using a plane-wave basis set. *Computational Materials Science* **1996,** *6* (1), 15-50.

25. Dudarev, S. L.; Botton, G. A.; Savrasov, S. Y.; Humphreys, C. J.; Sutton, A. P., Electron-energy-loss spectra and the structural stability of nickel oxide: An LSDA+U study. *Physical Review B* **1998,** *57* (3), 1505-1509.

26. Wang, L.; Maxisch, T.; Ceder, G., Oxidation energies of transition metal oxides within the \$\mathrm{GGA}+\mathrm{U}\$ framework. *Physical Review B* **2006,** *73* (19), 195107.

27. Grimme, S.; Antony, J.; Ehrlich, S.; Krieg, H., A consistent and accurate ab initio

parametrization of density functional dispersion correction (DFT-D) for the 94 elements H-Pu. *J. Chem. Phys.* **2010,** *132* (15).
28. Madsen, G. K. H.; Carrete, J.; Verstraete, M. J., BoltzTraP2, a program for interpolating band structures and calculating semi-classical transport coefficients. *Comput. Phys. Commun.* **2018,** *231*, 140-145.
29. Alex M. Ganose, A. J. J., David O. Scanlon, sumo: Command-line tools for plotting and analysis of periodic ab initio calculations. *Journal of Open Source Software* **2018,** *3* (28), 717.
